# Hypoglycemic Agent Targets and Susceptibility to Lower Respiratory Infection: A Pharmacogenomic Mendelian Randomization Study

**DOI:** 10.3390/genes17101258

**Published:** 2026-10-11

**Authors:** Taikang Yao, Nan Wu, Yanqiu Ma, Zilu Wang, Beibei Liu, Ming Lu, Zhenchao Wu, Ning Shen

**Affiliations:** 1 Department of Pulmonary and Critical Care Medicine, Peking University Third Hospital, No. 49 Huayuan North Road, Haidian District, Beijing 100191, China; 2 Center for Infectious Diseases, Peking University Third Hospital, No. 49 Huayuan North Road, Haidian District, Beijing 100191, China; 3 Department of Respiratory and Critical Care Medicine, Peking University People’s Hospital, No. 11 Xizhimen South Street, Xicheng District, Beijing 100044, China; 4 National Key Laboratory of Vascular Homeostasis and Remodeling, Peking University Health Science Center, No. 38 Xueyuan Road, Haidian District, Beijing 100191, China

**Keywords:** type 2 diabetes, lower respiratory tract infection, pneumonia, *PPARG*, drug-target mendelian randomization, genetic instruments, sepsis

## Abstract

**Background/Objectives**: Patients with type 2 diabetes face heightened risks of lower respiratory tract infections, but whether distinct glucose-lowering drug targets differentially alter pulmonary susceptibility remains unclear. We investigated how genetically proxied perturbations of major antidiabetic targets relate to respiratory infection and sepsis risks. **Methods**: Using two-sample drug-target Mendelian randomization, we examined genetic instruments proxying six antidiabetic pathways: insulin (*INSR*), sulfonylureas (*KCNJ11*/*ABCC8*), thiazolidinediones (*PPARG*), GLP-1 receptor agonists (*GLP1R*), SGLT2 inhibitors (*SLC5A2*), and metformin (*GPD1*, *GPD2*, *PRKAG2*). Associations were calibrated to standardized glycemic decrements and tested across 13 respiratory infection and sepsis GWAS outcomes. Analyses incorporated functional-variant comparisons, a type 2 diabetes positive control, and linkage disequilibrium adjustment. **Results**: Genetically proxied glucose lowering through *PPARG* showed consistent nominal associations with increased respiratory infection risk. Per 0.1 mmol/L lower glucose, the primary proxy and Pro12Ala functional variant were associated with higher odds of influenza and pneumonia (odds ratio [OR] 1.53, 95% CI 1.16–2.02, *p* = 0.003; and OR 1.53, 95% CI 1.13–2.06, *p* = 0.006) and other acute lower respiratory infections (OR 1.78, 95% CI 1.14–2.77, *p* = 0.011; and OR 1.87, 95% CI 1.16–3.01, *p* = 0.010). *PPARG* primary and functional proxies agreed in direction for 10/13 outcomes, whereas potassium-channel proxies agreed for only 3/13. Other targets showed distinct, heterogeneous patterns. Crucially, all signals were nominal and none survived false discovery rate correction (q ≥ 0.05). **Conclusions**: Genetically proxied glucose-lowering pathways exhibit divergent associations with respiratory infection susceptibility. While findings do not support class-wide causal risks, the repeated *PPARG* pattern highlights target-specific pulmonary immune mechanisms warranting functional and clinical investigation.

## 1. Introduction

Lower respiratory tract infections (LRTIs) remain a major global cause of death and include etiologically diverse bacterial, viral, and mycobacterial disease [1]. Diabetes adds a clinically important host dimension: hyperglycemia perturbs innate immune-cell metabolism and airway defense, while pulmonary infection can rapidly couple local inflammation to systemic organ injury [2,3,4]. Recent research by investigators in the present team has linked iron handling in immunocytes to pneumonia and sepsis through genetic analysis, examined sepsis risk in hospital-acquired pneumonia, and characterized difficult-to-treat Klebsiella pneumoniae infection [5,6,7]. These observations motivate target-resolved analysis of infection susceptibility.

Type 2 diabetes affects a large and growing population [8]. In contemporary cohorts, poor glycemic control and obesity track with severe influenza and pneumonia, and experimental high-glucose conditions blunt macrophage NOD1–MAPK responses to Legionella [3,4]. Metabolic and inflammatory signaling also intersect through adipokines and insulin sensitivity [9]. These pathways make glucose-lowering target biology a plausible determinant of pathogen-specific responses. Smoking is a strong independent determinant of LRTI and sepsis and may alter the phenotype distribution in which genetic target effects are observed [10].

Clinical studies report different infection outcomes across glucose-lowering treatments. Recent active-comparator cohorts and randomized-trial syntheses have examined pneumonia and sepsis during SGLT2 inhibitor and GLP-1 receptor agonist use, while metformin cohorts provide separate evidence on pneumonia incidence and survival [11,12,13,14,15,16,17]. Treatment allocation reflects diabetes duration, cardiometabolic disease, renal function, and smoking history. Genetic perturbations in target loci offer a complementary view of pathway biology, provided the exposure allele, outcome phenotype, and pharmacological mechanism are kept distinct.

Drug-target Mendelian randomization (MR) links cis-region variants to a molecular or downstream glycemic exposure and estimates associations with disease outcomes [18,19,20]. Investigators in this team have also applied MR to a respiratory symptom and lung cancer [21]. The approach is especially informative when a functional variant and a near-gene proxy can be compared within the same outcome GWAS. *PPARG* illustrates the central mechanistic question: the Pro12Ala allele has reduced transcriptional activity, whereas thiazolidinediones activate *PPARγ* [22,23]. *INSR* variation likewise indexes receptor-mediated signaling, and *KCNJ11*/*ABCC8* variation indexes an ATP-sensitive potassium-channel pathway. Their genetically proxied effects are therefore reported by target and variant, with treatment studies used for triangulation.

We evaluated genetically proxied *INSR*, *PPARG*, *KCNJ11*/*ABCC8*, *GLP1R*, *SLC5A2*, and putative metformin targets across bacterial, viral, tuberculosis, COVID-19, lower respiratory, and sepsis phenotypes. We combined the original target inventory with accession-matched re-estimation, functional-variant comparisons, and explicit accounting for case numbers. The scientific question is whether infection phenotypes differ across target pathways and whether those differences persist when outcome definitions and genetic proxies are aligned.

## 2. Materials and Methods

### 2.1. Study Design

**Figure 1:**
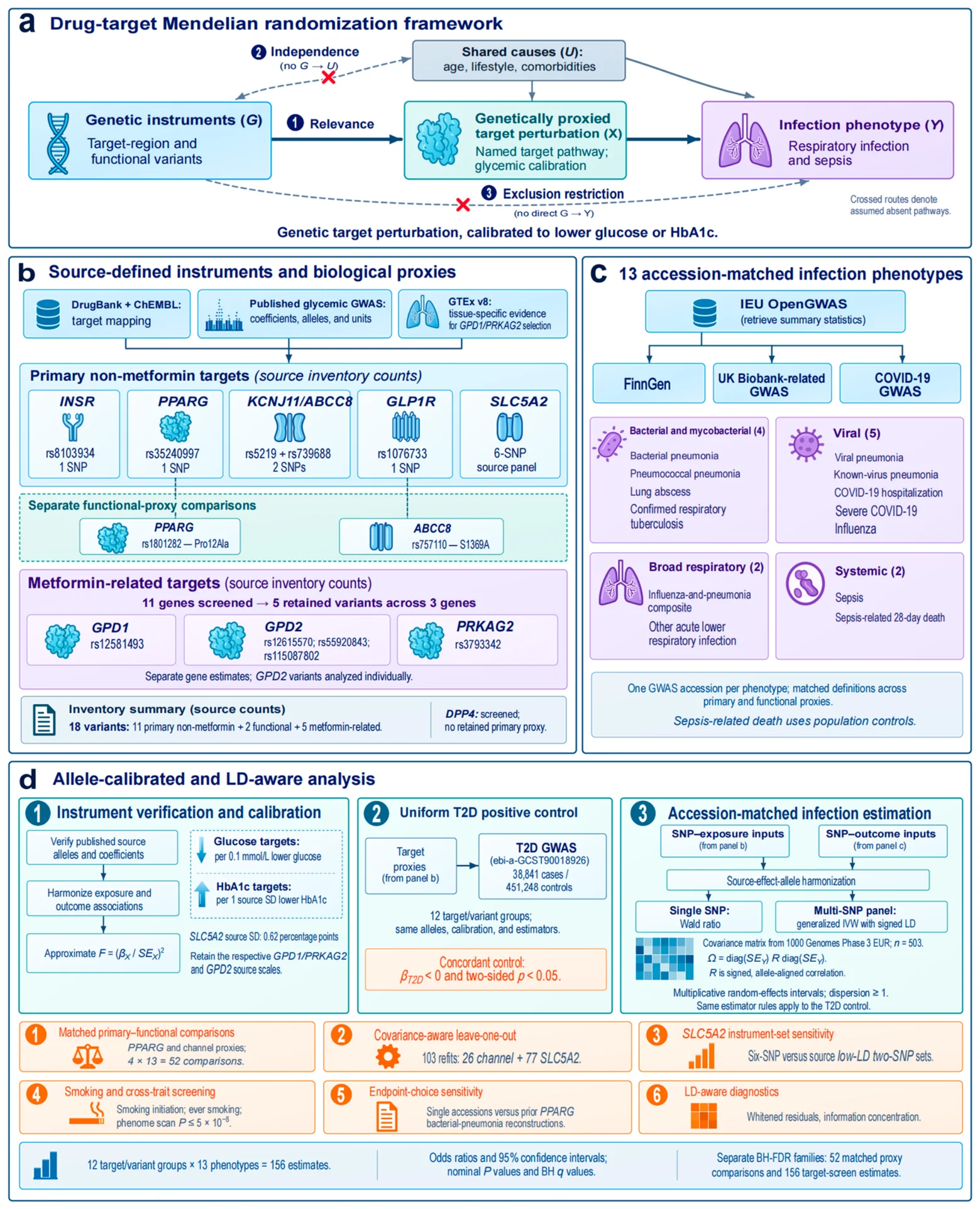
Study design and analytical workflow for drug-target Mendelian randomization. (**a**) Conceptual model linking genetic instruments (G), genetically proxied target perturbation (X), and infection outcomes (Y), with shared causes denoted by U. The three assumptions are instrument relevance, independence from shared causes, and exclusion of pathways from G to Y outside the nominated exposure. Red crosses on dashed connections indicate pathways assumed to be absent. (**b**) Instrument selection using DrugBank, ChEMBL, published glycemic GWAS, and GTEx v8 expression evidence. The inventory contains 18 variants: 11 primary non-metformin variants, two functional comparison variants, and five metformin-related variants across *GPD1*, *GPD2*, and *PRKAG2*. Metformin-related targets are analyzed separately, with individual estimates for the three *GPD2* variants. (**c**) Thirteen respiratory infection and sepsis phenotypes, each represented by one GWAS accession with the same outcome definition across primary and functional proxies. Sepsis-related 28-day death is compared with population controls. (**d**) Source-allele verification, harmonization, calibration to 0.1 mmol/L lower random glucose or one source SD lower HbA1c, and a common T2D positive control (ebi-a-GCST90018926; 38,841 cases and 451,248 controls). Single-variant analyses use Wald ratios; multi-variant analyses use generalized IVW with signed, allele-aligned LD from 503 European participants in 1000 Genomes Phase 3 and multiplicative random-effects intervals. Sensitivity analyses comprise functional-proxy comparisons, 103 covariance-aware leave-one-out refits, alternative *SLC5A2* instrument sets, smoking and cross-trait screening, endpoint comparisons, and LD-adjusted diagnostics. BH-FDR correction is applied separately to the 52 matched proxy comparisons and 156 estimates in the broader target screen. Abbreviations: *ABCC8*, ATP-binding cassette subfamily C member 8; BH, Benjamini–Hochberg; COVID-19, coronavirus disease 2019; diag, diagonal matrix; *DPP4*, dipeptidyl peptidase 4; EUR, European ancestry; F, instrument-strength statistic; FDR, false discovery rate; *GLP1R*, glucagon-like peptide 1 receptor; *GPD1*, glycerol-3-phosphate dehydrogenase 1; *GPD2*, glycerol-3-phosphate dehydrogenase 2; GTEx, Genotype-Tissue Expression; GWAS, genome-wide association study; HbA1c, glycated hemoglobin; IEU, Integrative Epidemiology Unit; *INSR*, insulin receptor; IVW, inverse-variance weighted; *KCNJ11*, potassium inwardly rectifying channel subfamily J member 11; LD, linkage disequilibrium; n, sample size; *PPARG*, peroxisome proliferator-activated receptor gamma; *PRKAG2*, protein kinase AMP-activated non-catalytic subunit gamma 2; Pro12Ala, proline-to-alanine substitution at residue 12; q, BH-adjusted p value; R, signed allele-dosage correlation matrix; S1369A, serine-to-alanine substitution at residue 1369; SD, standard deviation; SE, standard error; *SE_X_* and *SE_Y_*, standard errors of the variant–exposure and variant–outcome coefficients; *SLC5A2*, solute carrier family 5 member 2; SNP, single-nucleotide polymorphism; T2D, type 2 diabetes; UK, United Kingdom; *β*_T2D_, calibrated T2D coefficient; *β*_X_, variant–exposure coefficient; Ω, outcome covariance matrix.

We used two-sample drug-target MR to relate target-associated glycemic variants to respiratory infection and sepsis GWAS phenotypes (Figure 1). The analysis combined instrument characterization, a uniform T2D positive control, and target–infection estimation. Summary associations and study metadata were retrieved through the IEU OpenGWAS [24,25]. Each infection phenotype used one accession, retaining the same definition for primary and functional proxies. Supplementary Tables S1–S3 provide matched proxy results, outcome resources, and the T2D source. Reporting follows STROBE-MR [26].

### 2.2. Genetic Instruments for Glucose-Lowering Targets

We mapped glucose-lowering pathways to their encoding genes with DrugBank and ChEMBL and retained the primary and functional variants in Table 1 [19,27,28,29,30]. Source coefficients, effect alleles, tissue context, exposure units, and approximate F statistics are collected in Supplementary Table S4. *INSR*, *PPARG*, *KCNJ11*/*ABCC8*, and *GLP1R* glucose associations originated in a UK Biobank analysis of 309,895 White British participants [19]. The *SLC5A2* six-variant panel came from a separate HbA1c analysis of 344,182 participants; its published six-SNP and two-SNP sets used *r^2^* thresholds of 0.8 and 0.1, respectively [31]. Source-specific selection and LD adjustment were retained for each panel.

The primary variants were rs8103934 (*INSR*), rs1076733 (GLP1R), rs35240997 (*PPARG*), rs5219 and rs739688 (*KCNJ11*/*ABCC8*), and six *SLC5A2*-region variants. Functional comparisons used *PPARG* rs1801282 (Pro12Ala) and *ABCC8* rs757110 (S1369A) as separate proxies [22,23,32,33]. The source effect alleles were T for rs5219 and rs739688 and C for rs757110. Qin et al. also reported non-glycemic associations for selected variants [19]. These source details guided allele harmonization and the cross-trait sensitivity analyses.

*PPARG* Pro12Ala reduces receptor transcriptional activity, while thiazolidinediones activate *PPARγ* [22,23]. *ABCC8* S1369A changes ATP-sensitive potassium-channel sulfonylurea sensitivity [32,33]. We compared each functional variant with its primary target panel within the same outcome GWAS. This comparison tests consistency across biologically informative perturbations; the exposure throughout is the named genetic target pathway.

Metformin mechanisms include *AMPK*-dependent signaling, *PEN2*-linked lysosomal signaling, and mitochondrial glycerophosphate metabolism [34,35,36]. Metformin also increases the signaling metabolite Lac-Phe through mitochondrial effects in intestinal cells, linking it to appetite and energy balance [37]. The original 11-gene screen retained *GPD1* rs12581493, *PRKAG2* rs3793342, and three *GPD2* variants (Supplementary Table S5). *GPD1* and *PRKAG2* were selected with tissue-specific GTEx v8 expression evidence and calibrated with HbA1c associations in 344,182 participants [29,38,39]. *GPD2* calibration used a separate HbA1c GWAS of 389,889 participants [40]. We retained separate gene and variant estimates to preserve these biological distinctions.

### 2.3. Outcome GWAS and Phenotype Definitions

Thirteen accession-matched phenotypes were retained: bacterial pneumonia (7987 cases/188,868 controls), pneumococcal pneumonia (648/188,868), lung abscess (170/218,125), confirmed respiratory tuberculosis (247/217,599), viral pneumonia (700/188,868), known-virus pneumonia (763/212,707), COVID-19 hospitalization (9986/1,877,672), severe COVID-19 (5101/1,383,241), influenza (4262/188,868), influenza-and-pneumonia composite (29,924/188,868), other acute lower respiratory infection (10,103/208,689), sepsis (11,643/474,841), and sepsis-related 28-day death (1896/484,588) (Supplementary Table S2) [41,42]. Values are cases/controls in the named GWAS, not treated-patient counts. The death phenotype uses population controls and represents a population-based death endpoint.

### 2.4. Diabetes Positive Control

The biological positive control used the European T2D dataset ebi-a-GCST90018926 from Sakaue et al., comprising 38,841 cases and 451,248 controls [43]. The exact retained variants were carried into this single accession with the same source alleles, glycemic direction, and estimator used for infection outcomes. A concordant nominal control was defined consistently as a negative T2D coefficient per lower glycemic exposure with two-sided *p* < 0.05. We evaluated 12 target or variant groups, including individual *GPD2* variants. This control describes glycemic relevance on the specified source scale.

### 2.5. Statistical Analysis

Approximate instrument strength was calculated as *F* = (*β_X_*/*SE_X_*)^2^. MR interpretation assumes that the variants predict the specified exposure, are independent of shared causes of exposure and infection, and influence infection through the nominated target pathway. Exposure and outcome coefficients were aligned to the published effect allele before estimating a Wald ratio for one SNP or generalized IVW for a multi-SNP panel. For the latter, the outcome covariance matrix was Ω = diag(*SE_Y_*) R diag(*SE_Y_*), where R is the signed allele-dosage correlation matrix. The slope was *(βXᵀΩ^−1^β_Y_)/(βXᵀΩ^−1^β_X_)*. Its standard error used multiplicative random effects, with dispersion bounded below by one. We reconstructed R from complete genotypes of 503 European participants in 1000 Genomes Phase 3, accessed through Ensembl [44]. The channel pair had *r^2^* = 0.000427; the six *SLC5A2* variants required explicit LD adjustment.

A glucose-decreasing contrast of 0.1 mmol/L was expressed as −0.1 times the native outcome-to-glucose slope, with its standard error multiplied by 0.1. HbA1c target estimates used one source-standard-deviation decrease; Dai et al. describe the *SLC5A2* source SD as 0.62 percentage points [31]. *GPD1*/*PRKAG2* and *GPD2* retain their respective source HbA1c scales. We calculated two-sided Wald *p* values and 95% intervals after calibration. Gene-specific metformin estimates preserve their separate molecular and tissue contexts.

Benjamini–Hochberg q values were calculated for the 52 matched *PPARG*/channel proxy comparisons and, separately, the broader 156-estimate target screen. Leave-one-out analyses refitted the LD-adjusted model after removing each variant and its covariance row and column. These analyses generated 26 channel and 77 *SLC5A2* fits. We also compared the six-SNP and published low-LD two-SNP *SLC5A2* sets. Supplementary Table S6 records the diagnostic methods appropriate to each panel. Variant screening queried smoking initiation (ieu-b-4877; GSCAN) and ever smoking (ukb-b-20261), and the four principal/functional *PPARG* and channel variants were screened across OpenGWAS traits at *p* ≤ 5 × 10^−8^ [45].

Endpoint-choice sensitivity compared the calibrated single-accession results with the two *PPARG* bacterial-pneumonia reconstructions combining related endpoint releases. The present re-estimation additionally checked source alleles, applied the specified glycemic decrement, and incorporated the reconstructed reference LD matrices. Variant identity, outcome accession, source coefficients, and fitted estimates accompany the Supplement.

The analysis used R 4.5.3, TwoSampleMR 0.7.9, MRPRESSO 1.0, mr.raps 0.4.3, and ieugwasr 1.1.0. The allele-calibrated and LD-adjusted calculations used Python 3.12.14 and NumPy 2.5.3. The analysis specifies its 1000 Genomes reference, covariance model, source-specific selection rules, and retained variants. Associations are reported as nominal unless a q value is given.

## 3. Results

### 3.1. Target Instruments Represent Distinct Glycemic Pathways

**Figure 2:**
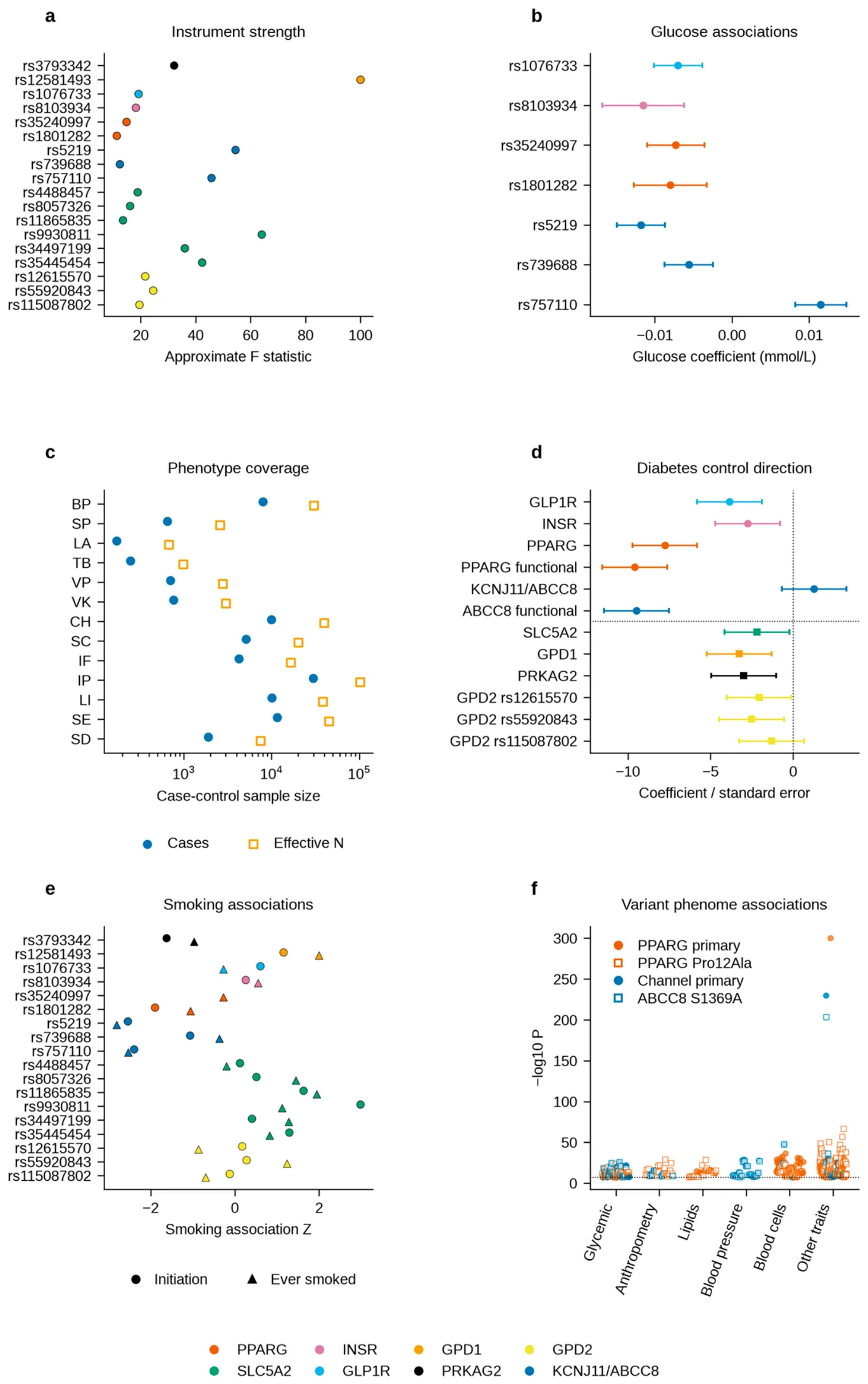
Instrument characteristics, outcome sample sizes, and cross-trait associations. (**a**) Approximate *F* statistics for all 18 variants, calculated as the squared ratio of the source exposure coefficient to its standard error. (**b**) Random-glucose association coefficients and 95% CIs for seven variants, aligned to the source effect allele. (**c**) Case counts (filled circles) and effective case–control sample sizes (open squares) for the 13 outcomes, shown on a logarithmic axis; effective sample size is 4/(1/*n_cases_* + 1/*n_controls_*). (**d**) T2D positive-control coefficients divided by their standard errors for 12 target or variant groups, with horizontal intervals of ±1.96 around each standardized coefficient. Circles denote estimates calibrated to 0.1 mmol/L lower random glucose; squares denote estimates calibrated to one source SD lower HbA1c. The vertical dotted line marks zero, and the horizontal dotted line separates the two calibration groups. (**e**) Allele-aligned Z statistics for 35 smoking associations: 17 smoking-initiation records (circles) and 18 ever-smoking records (triangles). (**f**) Cross-trait associations for *PPARG* rs35240997, *PPARG* rs1801282 (Pro12Ala), *KCNJ11* rs5219, and *ABCC8* rs757110 (S1369A), comprising 475 records with *p* ≤ 5 × 10^−8^. Filled circles denote primary proxies and open squares denote functional proxies; vermilion identifies *PPARG* and blue identifies *KCNJ11*/*ABCC8*. The vertical axis shows −log_10_(p), and the dotted horizontal line marks *p* = 5 × 10^−8^. Values returned as *p* = 0 are plotted using a floor of 10^−300^; separate studies of the same trait remain separate records. Colors in panels (**a**,**b**,**d**,**e**) identify target groups as indicated in the figure. Abbreviations: *ABCC8*, ATP-binding cassette subfamily C member 8; BP, bacterial pneumonia; CH, COVID-19 hospitalization; CI, confidence interval; COVID-19, coronavirus disease 2019; *GLP1R*, glucagon-like peptide 1 receptor; *GPD1*, glycerol-3-phosphate dehydrogenase 1; *GPD2*, glycerol-3-phosphate dehydrogenase 2; HbA1c, glycated hemoglobin; IF, influenza of all types; *INSR*, insulin receptor; IP, influenza and pneumonia composite; *KCNJ11*, potassium inwardly rectifying channel subfamily J member 11; LA, lung abscess; LI, other acute lower respiratory infections; N, sample size; *n_cases_*, case count; *n_controls_*, control count; *PPARG*, peroxisome proliferator-activated receptor gamma; *PRKAG2*, protein kinase *AMP*-activated non-catalytic subunit gamma 2; Pro12Ala, proline-to-alanine substitution at residue 12; S1369A, serine-to-alanine substitution at residue 1369; SC, severe COVID-19; SD, standard deviation (exposure unit) or sepsis-related 28-day death (phenotype code); SE, sepsis (phenotype code); *SLC5A2*, solute carrier family 5 member 2; SP, pneumococcal pneumonia; T2D, type 2 diabetes; TB, confirmed respiratory tuberculosis; VK, pneumonia due to a known virus excluding influenza; VP, viral pneumonia; Z, association coefficient divided by its standard error.

The source inventory contained one primary variant each for *GLP1R*, *INSR*, and *PPARG*; two for *KCNJ11*/*ABCC8*; six for *SLC5A2*; and five retained variants across *GPD1*, *GPD2*, and *PRKAG2*. The *PPARG* and *KCNJ11*/*ABCC8* functional variants were analyzed separately. Instrument-level alleles and F statistics are provided in Supplementary Table S4 (Figure 2). The *PPARG* primary and Pro12Ala *F* statistics were approximately 14.76 and 11.11; the two *KCNJ11*/*ABCC8* primary *F* statistics were 54.39 and 12.25. These denominators help explain the broad intervals when an outcome has few cases.

The metformin screen retained five variants across *GPD1*, *GPD2*, and *PRKAG2* (Supplementary Table S5). Their source HbA1c GWAS and tissue-selection contexts define distinct gene-level perturbations. *GPD2* expression-selection labels were preserved, and its three retained variants were displayed separately on their source HbA1c scale. The analysis therefore provides a target-resolved comparison of the molecular pathways associated with glucose regulation.

### 3.2. Target Perturbations Map Phenotype-Specific Infection Associations

**Figure 3:**
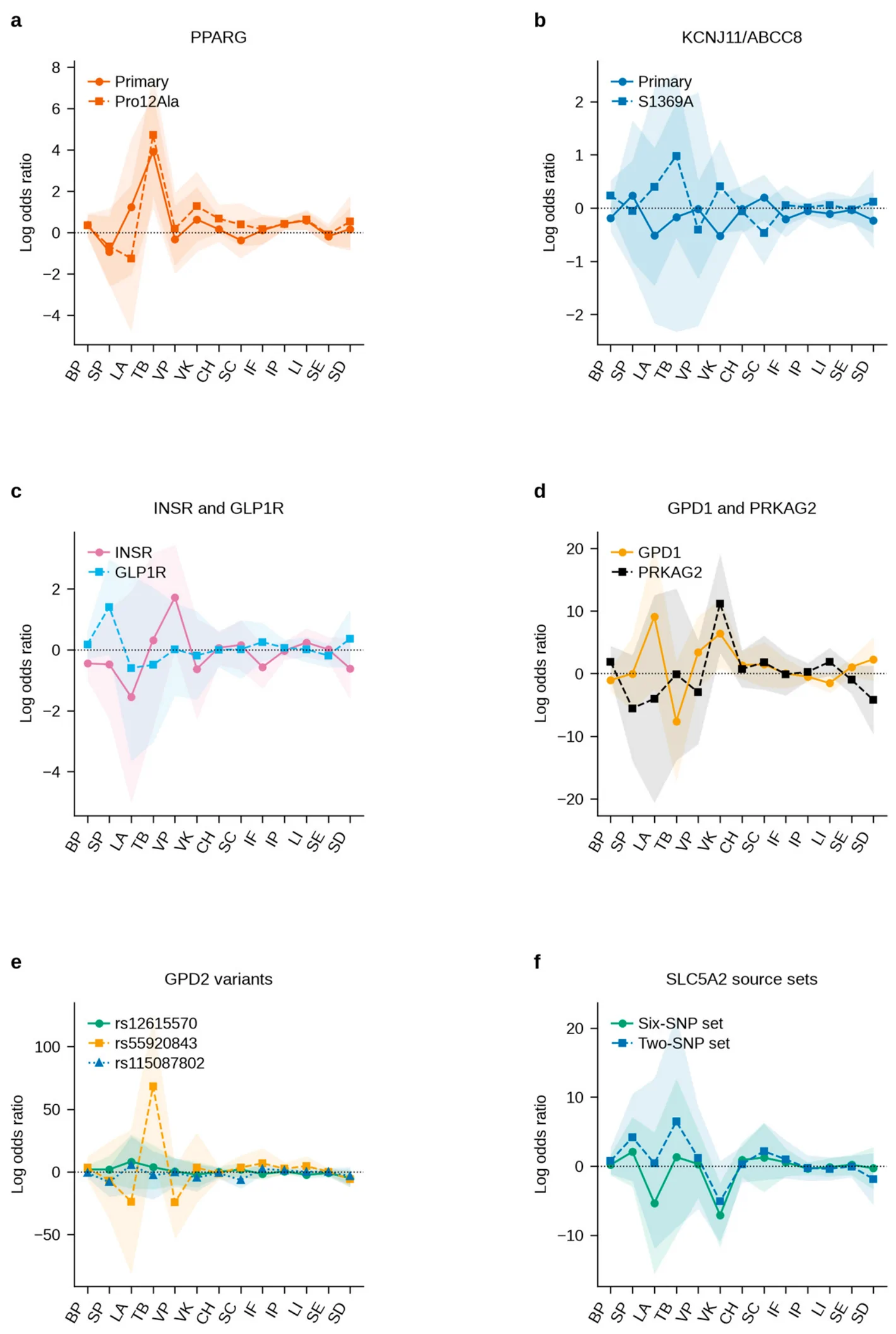
Associations of glucose-lowering genetic proxies with infection phenotypes. (**a**) *PPARG* primary rs35240997 and functional rs1801282 (Pro12Ala) proxies. (**b**) The primary *KCNJ11*/*ABCC8* panel (rs5219 and rs739688) and functional *ABCC8* rs757110 (S1369A) proxy. In panels (**a**,**b**), circles with solid lines denote primary proxies and squares with dashed lines denote functional proxies. (**c**) *INSR* rs8103934 (pink circles) and *GLP1R* rs1076733 (light-blue squares). (**d**) *GPD1* rs12581493 (orange circles) and *PRKAG2* rs3793342 (black squares), analyzed separately. (**e**) Individual *GPD2* variants rs12615570 (green circles), rs55920843 (orange squares), and rs115087802 (blue triangles). (**f**) *SLC5A2* six-variant (green circles) and published low-LD two-variant (blue squares) panels. Points represent calibrated log ORs across 13 outcome accessions, and shaded ribbons indicate 95% CIs. Connecting lines aid comparison across categorical outcomes; dotted horizontal lines mark zero. Panels (**a**–**c**) use 0.1 mmol/L lower random glucose, and panels (**d**–**f**) use one source SD lower HbA1c, retaining each target’s source calibration. Single-variant estimates use Wald ratios. Multi-variant estimates use generalized IVW with signed, allele-aligned LD from 503 European participants in 1000 Genomes Phase 3 and multiplicative random-effects intervals. The *SLC5A2* low-LD panel comprises rs9930811 and rs35445454. For COVID-19 hospitalization, the larger panel contains five available variants and the low-LD panel contains only rs9930811. Abbreviations: *ABCC8*, ATP-binding cassette subfamily C member 8; BP, bacterial pneumonia; CH, COVID-19 hospitalization; CI, confidence interval; COVID-19, coronavirus disease 2019; *GLP1R*, glucagon-like peptide 1 receptor; *GPD1*, glycerol-3-phosphate dehydrogenase 1; *GPD2*, glycerol-3-phosphate dehydrogenase 2; HbA1c, glycated hemoglobin; IF, influenza of all types; *INSR*, insulin receptor; IP, influenza and pneumonia composite; IVW, inverse-variance weighted; *KCNJ11*, potassium inwardly rectifying channel subfamily J member 11; LA, lung abscess; LD, linkage disequilibrium; LI, other acute lower respiratory infections; OR, odds ratio; *PPARG*, peroxisome proliferator-activated receptor gamma; *PRKAG2*, protein kinase *AMP*-activated non-catalytic subunit gamma 2; Pro12Ala, proline-to-alanine substitution at residue 12; S1369A, serine-to-alanine substitution at residue 1369; SC, severe COVID-19; SD, standard deviation (exposure unit) or sepsis-related 28-day death (phenotype code); SE, sepsis (phenotype code); *SLC5A2*, solute carrier family 5 member 2; SNP, single-nucleotide polymorphism; SP, pneumococcal pneumonia; TB, confirmed respiratory tuberculosis; VK, pneumonia due to a known virus excluding influenza; VP, viral pneumonia.

Figure 3 displays calibrated target trajectories across the same 13 outcome definitions. Each panel retains its target-specific exposure unit, so direction and phenotype pattern can be read alongside uncertainty. Twelve target or variant groups produced 156 estimates. Their distribution shows that respiratory infection associations differ across receptor, channel, transporter, and metformin-related pathways. The functional-proxy comparisons below identify the most repeated pattern.

### 3.3. A Common Diabetes GWAS Defines the Positive-Control Comparison

The common-accession T2D results are shown in Supplementary Figure S1 and Supplementary Table S7. Ten of 12 target or variant groups met the uniformly applied negative-direction and nominal-P criterion. Per 0.1 mmol/L lower glucose, *PPARG* primary and Pro12Ala ORs were 0.368 and 0.265, respectively. The LD-adjusted *SLC5A2* coefficient was −0.635 (SE 0.289; *p* = 0.028) per source HbA1c SD decrease. *GPD1* and *PRKAG2* were supported using their actual retained single variants. The two-SNP *KCNJ11*/*ABCC8* panel had heterogeneous SNP directions and a positive pooled coefficient (0.388; SE 0.307; *p* = 0.206), while *ABCC8* S1369A had a negative control coefficient. *GPD2* rs115087802 had a negative but imprecise coefficient (*p* = 0.192). These contrasts distinguish the genetic perturbations within each pathway.

### 3.4. Target Perturbations and Infection Phenotypes

Supplementary Table S1 reports all 52 accession-matched *PPARG* and channel proxy estimates with their exposure unit, confidence interval, nominal *p* value, and q values. The complete 156-estimate target screen is supplied with the harmonized association inputs.

#### 3.4.1. PPARG-Mediated Perturbation

Per 0.1 mmol/L lower glucose, the *PPARG* primary proxy was associated with the influenza-and-pneumonia composite (finn-b-J10_INFLUPNEU; 29,924 cases): OR 1.53 (95% CI 1.16–2.02; *p* = 0.0028). Pro12Ala showed a closely aligned estimate, OR 1.53 (1.13–2.06; *p* = 0.0056). For other acute lower respiratory infection (finn-b-J10_LOWERINF; 10,103 cases), the corresponding estimates were OR 1.78 (1.14–2.77; *p* = 0.0109) and OR 1.87 (1.16–3.01; *p* = 0.0105). Together, these two diagnostic groups show a repeated nominal PPARG-associated respiratory pattern.

Both PPARG variants also pointed toward higher odds of confirmed respiratory tuberculosis, based on 247 cases: primary OR 49.95 (95% CI 3.25–768.33; *p* = 0.0050) and Pro12Ala OR 111.61 (5.79–2152.96; *p* = 0.0018). The small case count and glycemic denominator make this a directionally informative but imprecise estimate. Experimental studies connect *PPAR*γ activity with macrophage responses during tuberculosis and viral lung injury [46,47].

For the single bacterial-pneumonia accession finn-b-J10_PNEUMOBACT, *PPARG* primary and functional estimates were OR 1.44 (95% CI 0.87–2.39; *p* = 0.159) and OR 1.41 (0.82–2.44; *p* = 0.214). Their similar point directions extend the respiratory pattern, while the intervals include unity. The comparison with related-endpoint combinations shows how phenotype definition changes precision.

The matched *PPARG* primary estimates were OR 0.83 (95% CI 0.54–1.28; *p* = 0.394) for sepsis occurrence and OR 1.18 (0.41–3.38; *p* = 0.752) for sepsis-related 28-day death. Both are population-control contrasts with intervals spanning unity. Their different case definitions separate susceptibility from the population probability of sepsis-related death.

#### 3.4.2. *KCNJ11*/*ABCC8* Channel Perturbation

The LD-adjusted two-SNP *KCNJ11*/*ABCC8* bacterial-pneumonia estimate was OR 0.83 (95% CI 0.64–1.08; *p* = 0.1570). The functional *ABCC8* S1369A estimate pointed in the other direction, OR 1.27 (95% CI 0.95–1.69; *p* = 0.1042). Source-allele alignment therefore identifies different genetic channel perturbations within the same infection phenotype.

For severe COVID-19 (ebi-a-GCST011075), the primary channel estimate was OR 1.22 (95% CI 0.79–1.88; *p* = 0.3640), while the functional estimate was OR 0.63 (95% CI 0.35–1.14; *p* = 0.1251). These population-control GWAS combine susceptibility and progression components. The genotype-specific directions are interpreted together with their broad intervals and differing T2D control patterns.

Figure 4 compares primary and functional proxy directions and displays LD-adjusted sensitivity structure. *PPARG* proxies agreed in direction for 10/13 matched phenotypes; channel proxies agreed for 3/13. The six nominal *PPARG* associations involved confirmed tuberculosis, influenza and pneumonia, and other acute lower respiratory infection, with q values of 0.072–0.095 across 52 tests. None of the 156 broader-screen estimates had q < 0.05. Supplementary Figures S2–S14 display all four proxy estimates within each matched phenotype.

Supplementary Figures S15–S20 show calibrated proxy contrasts, relations between case count and precision, and sensitivity to endpoint-release choice. These comparisons retain the *PPARG* direction for the influenza-and-pneumonia composite and distinguish agreement within *PPARG* from the channel-variant contrast.

#### 3.4.3. INSR-Mediated Insulin Signaling

The *INSR* rs8103934 bacterial-pneumonia estimate was OR 0.64 (95% CI 0.37–1.09; *p* = 0.0986) per 0.1 mmol/L lower glucose. The point direction motivates investigation of receptor-mediated host responses, with the interval showing the precision available. This exposure concerns INSR signaling and is biologically distinct from exogenous insulin administration.

#### 3.4.4. Metformin-Related Molecular Targets

*GPD1*, *GPD2*, and *PRKAG2* were evaluated separately using their published glycemic coefficients and tissue-selection contexts. The matched *INSR*, *GPD1*, and *PRKAG2* trajectories appear in Supplementary Figures S21–S23. Experimental models and clinical cohorts provide complementary evidence on metformin-linked macrophage, lung inflammatory, pneumonia, and septic-shock outcomes [12,48,49,50,51]. The present gene-level estimates define specific molecular comparisons within that broader biology.

#### 3.4.5. Other Target Perturbations

The *GLP1R* and LD-adjusted *SLC5A2* trajectories are presented in Supplementary Figures S24 and S25. *SLC5A2* had a nominal association with known-virus pneumonia (coefficient −7.11; SE 2.37; *p* = 0.0027 per source HbA1c SD decrease), with a wide interval on the odds scale and q = 0.145 across the broader screen. Its common-GWAS diabetes control supported the intended glycemic direction. Contemporary SGLT2 inhibitor cohorts and trial syntheses provide an external clinical comparison [13,14,15,17].

Supplementary Figure S26 shows variant-level T2D control associations using the same source alleles as the infection models. The aligned *PPARG* proxies both pointed toward lower T2D odds, while the two primary channel variants had different directions. This variant-level view explains the control pattern without averaging biologically different perturbations.

The LD-adjusted sensitivity analyses generated 103 leave-one-out fits, including 77 *SLC5A2* fits across 13 phenotypes. Supplementary Figures S27–S39 show association fits, individual-SNP estimates, funnel distributions, whitened residual diagnostics, covariance-aware leave-one-out estimates, and information concentration for each *SLC5A2* endpoint. Twelve outcomes retained six variants; COVID-19 hospitalization retained five. The case count, source unit, and actual instrument count are reported for each comparison.

The genome-wide-significant cross-trait screen returned 475 association records for the four named *PPARG* and channel variants, spanning glycemic, blood-cell, lipid, anthropometric, and other phenotypes (Supplementary Figure S40). Smoking initiation and ever-smoking queries resolved 35 of 36 planned variant–trait pairs; six had nominal *p* < 0.05 and none had BH q < 0.05 (Supplementary Figure S41). These screens characterize shared genetic associations and identify specific pleiotropy questions.

The three *GPD2* variants are displayed individually across all 13 infection accessions in Supplementary Figure S42. Supplementary Figure S43 compares the six-SNP and published two-SNP *SLC5A2* sets, each fitted with its own LD submatrix. Together, these analyses examine instrument membership while retaining the source target and glycemic contrast.

## 4. Discussion

The clearest infection pattern in our matched analysis involved *PPARG* perturbation. The near-gene and functional variants showed aligned nominal associations with the influenza-and-pneumonia composite and other acute lower respiratory infection, and both pointed in the same direction for confirmed respiratory tuberculosis. Comparing the two proxies within the same outcome GWAS connects this repeated pattern to a defined target pathway. Separate analyses of channel, receptor, and metformin-related targets reveal how infection associations vary across the biological pathways involved in glucose regulation.

Hyperglycemia affects pulmonary immune metabolism, pathogen handling, and the transition from local inflammation to systemic infection [2,3,4,52]. Autophagy and iron-dependent lipid damage further link antimicrobial defense to lung and systemic tissue injury [53]. The present team’s earlier genetic analysis connected immunocyte iron metabolism to pneumonia and sepsis, while its hospital-acquired-pneumonia work identified clinical predictors of sepsis [5,6]. These complementary lines suggest that infection susceptibility in diabetes reflects both host metabolic state and pathogen context. Our target-specific comparison adds genetic evidence to this framework.

*PPARG* biology connects receptor activity with cell type and pathogen context. Pioglitazone increased proinflammatory responses in bronchial epithelial cells during experimental Pseudomonas pneumonia, whereas macrophage *PPAR*γ activity has been associated with control of influenza-induced lung inflammation [46,54]. Tuberculosis studies also implicate PPARγ in macrophage–pathogen interactions [47]. Pro12Ala reduces receptor transcriptional activity, while thiazolidinediones activate *PPAR*γ. The aligned near-gene and functional-variant estimates motivate experiments that distinguish these perturbation directions across airway epithelial cells and macrophages.

The *PPARG* findings also identify a useful phenotype pattern. The influenza-and-pneumonia composite and other acute lower respiratory infection capture different parts of the respiratory disease spectrum, yet the two proxies point in the same direction in both. Confirmed tuberculosis adds a mycobacterial phenotype to this pattern. This comparison focuses follow-up on the relationship between *PPARG* signaling and pulmonary host defense, with pathogen-specific phenotypes helping to identify the relevant cellular responses.

Sepsis occurrence and sepsis-related 28-day death describe two population-based phenotypes. Their different case definitions help explain why a target can show different point directions across these GWAS. Mortality conditional on a sepsis diagnosis is a separate case-only contrast. Current sepsis immunology emphasizes changing mixtures of neutrophil, macrophage, and pyroptotic responses, and proteomic studies identify further target diversity [52,55,56]. Together, these observations motivate evaluation of target signaling across infection stages using outcome definitions that match each clinical question.

The channel findings illustrate genetic heterogeneity within a therapeutic pathway. The primary *KCNJ11*/*ABCC8* panel and functional *ABCC8* variant agreed in three of 13 phenotype directions and differed in their diabetes controls. Their opposing point directions for bacterial pneumonia and severe COVID-19 identify a variant-specific question about channel signaling. INSR adds a separate receptor-mediated comparison. These results link respiratory phenotypes to defined genetic perturbations and complement clinical treatment studies.

Metformin’s *GPD1*, *GPD2*, and *PRKAG2* axes involve cytosolic glycerol metabolism, mitochondrial redox biology, and AMPK signaling [34,35,36]. Metformin also increases the signaling metabolite Lac-Phe through mitochondrial effects in intestinal cells, linking the drug to appetite and energy-balance pathways [37]. Keeping the three genetic targets separate preserves their molecular and tissue contexts and supports direct examination of each infection trajectory. Experimental models have identified macrophage antimicrobial and lung inflammatory responses to metformin, and clinical cohorts have reported pneumonia and septic-shock outcomes among metformin users [12,16,48,49,50,51]. Future studies can test each molecular axis using tissue-specific expression and infection-specific colocalization.

Smoking-related pathways merit consideration in target-region MR. The variant screen recovered six nominal smoking-trait associations among 35 resolved records; the minimum BH q value was 0.086. These results identify candidate behavioral or pleiotropic links for further study. The broader cross-trait screen also connected target variants with blood-cell, lipid, and anthropometric traits. Smoking-stratified GWAS and multivariable analyses can test whether those pathways contribute to target–infection associations [10,20,57].

### Interpretation and Future Work

This study brings together a curated target inventory, functional-variant comparisons, a T2D control recheck, and infection phenotypes matched to specific GWAS accessions. The comparison identifies a repeated *PPARG*-associated pattern across related lower respiratory phenotypes and preserves the biological identity of channel, receptor, and metformin-related pathways. Naming the exact genetic proxy and outcome accession makes the target-specific findings directly comparable with future molecular and clinical studies.

The statistical interpretation rests on exposure calibration and outcome precision. Applying the specified 0.1 mmol/L glucose decrement yields modest *PPARG* odds ratios for the two larger respiratory phenotypes, while tuberculosis remains imprecise because of its 247 cases and small genetic glucose coefficients. Winner’s curse and exposure-association error can affect Wald ratios despite approximate F statistics above 10. The highlighted associations are nominal; q values summarize the multiple-testing context. Single-SNP comparisons and the smoking/cross-trait screens assess different aspects of proxy validity, and the smoking screen estimates variant associations and preserves the distinction from a smoking-adjusted infection coefficient. Potential overlap between UK Biobank-derived exposures and some outcome resources could correlate association errors; participant overlap could not be quantified from summary metadata. With weaker instruments, this correlation can draw estimates toward confounded observational associations. The functional comparison uses the same source populations and is biological corroboration within the source data.

The *PPARG* findings prioritize tissue-resolved genetic and functional studies of pulmonary host defense. Independent-ancestry GWAS, infection-specific colocalization, and allele-specific assays can test whether receptor transcription and downstream immune metabolism account for the repeated respiratory pattern. Separate *GPD1*, *GPD2*, and *PRKAG2* analyses can identify which components of metformin-related biology contribute to host responses. Clinical cohorts with smoking, glycemic history, and medication timing will provide a complementary test of how these pathways operate during treatment.

## 5. Conclusions

Genetically proxied glucose-lowering targets show distinct respiratory infection association patterns. The clearest repeated nominal pattern, which did not meet the FDR threshold, involved *PPARG* rs35240997 and Pro12Ala for influenza and pneumonia and other acute lower respiratory infection, with additional imprecise directionality in confirmed tuberculosis. Source-allele calibration distinguishes the channel variants, and LD-adjusted analyses describe *SLC5A2* sensitivity across phenotypes. Separate metformin-related gene estimates preserve their molecular biology. These results focus further investigation on *PPARG*-related pulmonary host defense and provide defined genetic comparisons for clinical and functional studies.

## Figures and Tables

**Figure 4:**
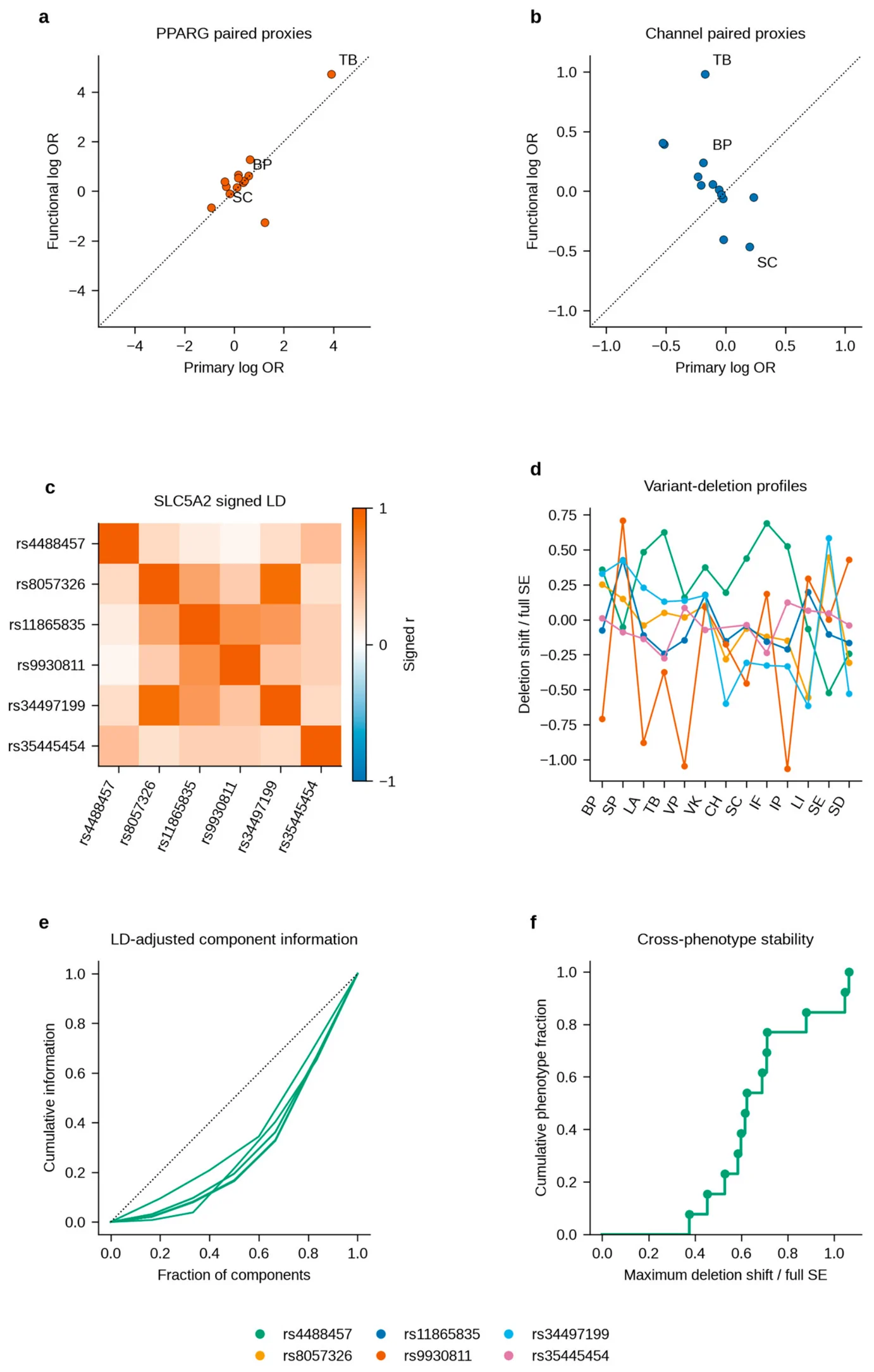
Agreement between genetic proxies and *SLC5A2* sensitivity analyses. (**a**) *PPARG* primary rs35240997 versus functional rs1801282 (Pro12Ala) estimates. (**b**) Primary *KCNJ11*/*ABCC8* rs5219–rs739688 estimates versus functional *ABCC8* rs757110 (S1369A) estimates. Each point represents one of 13 matched outcome accessions; axes show log ORs per 0.1 mmol/L lower random glucose. Dotted diagonals indicate equal coefficients, with directional agreement for 10/13 *PPARG* outcomes and 3/13 channel outcomes. (**c**) Signed, effect-allele-aligned LD correlations among the six *SLC5A2* variants, reconstructed from 503 European participants in 1000 Genomes Phase 3; the color scale gives the correlation coefficient r. (**d**) Changes in *SLC5A2* estimates after omitting each indicated variant, divided by the complete-panel standard error. Each deletion refits generalized IVW using the retained variants and corresponding LD submatrix; colors identify the omitted variants. (**e**) Cumulative information curves for all 13 outcomes after Cholesky whitening using the outcome covariance matrix. Squared transformed exposure coefficients are normalized to sum to one, sorted in ascending order, and accumulated; the dotted diagonal represents equal information across components. These components are linear combinations of variants in the fixed source order. (**f**) Empirical cumulative distribution of the largest absolute standardized deletion shift for each outcome. *SLC5A2* estimates are calibrated to one source SD lower HbA1c. COVID-19 hospitalization includes five available variants; the other outcomes include six. Abbreviations: *ABCC8*, ATP-binding cassette subfamily C member 8; BP, bacterial pneumonia; CH, COVID-19 hospitalization; COVID-19, coronavirus disease 2019; HbA1c, glycated hemoglobin; IF, influenza of all types; IP, influenza and pneumonia composite; IVW, inverse-variance weighted; *KCNJ11*, potassium inwardly rectifying channel subfamily J member 11; LA, lung abscess; LD, linkage disequilibrium; LI, other acute lower respiratory infections; OR, odds ratio; *PPARG*, peroxisome proliferator-activated receptor gamma; Pro12Ala, proline-to-alanine substitution at residue 12; S1369A, serine-to-alanine substitution at residue 1369; SC, severe COVID-19; SD, standard deviation (exposure unit) or sepsis-related 28-day death (phenotype code); SE, standard error (statistical quantity) or sepsis (phenotype code); *SLC5A2*, solute carrier family 5 member 2; SP, pneumococcal pneumonia; TB, confirmed respiratory tuberculosis; VK, pneumonia due to a known virus excluding influenza; VP, viral pneumonia.

**Table 1:** Genetic instruments for glucose-lowering target perturbation.

Pathway Motivating Target Selection	Proxy Target	Encoding Gene (s)	Retained Instrument (s)
Insulin/insulin analogs	Insulin receptor	*INSR*	rs8103934 (1 SNP)
Sulfonylureas	ATP-sensitive K+ channel	*KCNJ11*, *ABCC8*	rs5219 + rs739688 (2 SNPs); functional sensitivity variant rs757110
Thiazolidinediones	PPAR-gamma	*PPARG*	rs35240997 (1 SNP); functional sensitivity variant rs1801282
GLP-1 receptor agonists	GLP-1 receptor	*GLP1R*	rs1076733 (1 SNP)
SGLT2 inhibitors	Sodium-glucose cotransporter 2	*SLC5A2*	6 SNPs
Metformin	Putative targets retained after screening	*GPD1*, *GPD2*, *PRKAG2*	rs12581493; rs12615570, rs55920843, rs115087802; rs3793342 (5 SNPs total retained from 11 genes screened)
DPP-4 inhibitors	Dipeptidyl peptidase 4	*DPP4*	Secondary cis-variant panel screened; no validated primary proxy taken forward

The table identifies molecular targets and the exact variants retained for the primary and functional analyses. Estimates describe allele-associated target perturbations on the specified glycemic scale. Medication classes indicate the pathway that motivated instrument selection.

## Data Availability

Frozen summary-association inputs, calibrated estimates, reference LD matrices, and analysis and plotting scripts accompany the Supplementary Materials. FinnGen and IEU OpenGWAS provide the underlying outcome resources, with specific accessions reported in the Supplement. The reference LD analysis used publicly available 1000 Genomes Phase 3 genotypes through Ensembl. This study generated no new participant observations.

## Supplementary Materials

The following supporting information can be downloaded at: https://www.mdpi.com/article/10.3390/genes17101258/s1, Figure S1: Type 2 diabetes positive-control associations for 12 target or variant groups; Figure S2: Associations of *PPARG* and *KCNJ11*/*ABCC8* proxies with bacterial pneumonia; Figure S3: Associations of *PPARG* and *KCNJ11*/*ABCC8* proxies with pneumococcal pneumonia; Figure S4: Associations of *PPARG* and *KCNJ11*/*ABCC8* proxies with lung abscess; Figure S5: Associations of *PPARG* and *KCNJ11*/*ABCC8* proxies with confirmed respiratory tuberculosis; Figure S6: Associations of *PPARG* and *KCNJ11*/*ABCC8* proxies with viral pneumonia; Figure S7: Associations of *PPARG* and *KCNJ11*/*ABCC8* proxies with viral pneumonia due to a known virus excluding influenza; Figure S8: Associations of *PPARG* and *KCNJ11*/*ABCC8* proxies with COVID-19 hospitalization; Figure S9: Associations of *PPARG* and *KCNJ11*/*ABCC8* proxies with severe COVID-19; Figure S10: Associations of *PPARG* and *KCNJ11*/*ABCC8* proxies with influenza; Figure S11: Associations of *PPARG* and *KCNJ11*/*ABCC8* proxies with influenza and pneumonia composite; Figure S12: Associations of *PPARG* and *KCNJ11*/*ABCC8* proxies with other acute lower respiratory infections; Figure S13: Associations of *PPARG* and *KCNJ11*/*ABCC8* proxies with sepsis; Figure S14: Associations of *PPARG* and *KCNJ11*/*ABCC8* proxies with sepsis-related 28-day death; Figure S15: Paired *PPARG* proxy estimates across 13 infection and sepsis phenotypes; Figure S16: Paired *KCNJ11*/*ABCC8* proxy estimates across 13 infection and sepsis phenotypes; Figure S17: Outcome case counts and standard errors of *PPARG* proxy estimates; Figure S18: Outcome case counts and standard errors of *KCNJ11*/*ABCC8* proxy estimates; Figure S19: Sensitivity of *PPARG* bacterial pneumonia estimates to endpoint selection; Figure S20: Directional agreement between primary and functional proxies across matched outcomes; Figure S21: Associations of the *INSR* genetic proxy with infection and sepsis phenotypes; Figure S22: Associations of the *GPD1* genetic proxy with infection and sepsis phenotypes; Figure S23: Associations of the *PRKAG2* genetic proxy with infection and sepsis phenotypes; Figure S24: Associations of the *GLP1R* genetic proxy with infection and sepsis phenotypes; Figure S25: Associations of the *SLC5A2* genetic instrument set with infection and sepsis phenotypes; Figure S26: Variant-level type 2 diabetes positive-control associations; Figure S27: LD-adjusted analyses of *SLC5A2* genetic instruments for bacterial pneumonia; Figure S28: LD-adjusted analyses of *SLC5A2* genetic instruments for pneumococcal pneumonia; Figure S29: LD-adjusted analyses of *SLC5A2* genetic instruments for lung abscess; Figure S30: LD-adjusted analyses of *SLC5A2* genetic instruments for confirmed respiratory tuberculosis; Figure S31: LD-adjusted analyses of *SLC5A2* genetic instruments for viral pneumonia; Figure S32: LD-adjusted analyses of *SLC5A2* genetic instruments for pneumonia due to a known virus excluding influenza; Figure S33: LD-adjusted analyses of *SLC5A2* genetic instruments for COVID-19 hospitalization; Figure S34: LD-adjusted analyses of *SLC5A2* genetic instruments for severe COVID-19; Figure S35: LD-adjusted analyses of *SLC5A2* genetic instruments for influenza of all types; Figure S36: LD-adjusted analyses of *SLC5A2* genetic instruments for influenza and pneumonia composite; Figure S37: LD-adjusted analyses of *SLC5A2* genetic instruments for other acute lower respiratory infections; Figure S38: LD-adjusted analyses of *SLC5A2* genetic instruments for sepsis; Figure S39: LD-adjusted analyses of *SLC5A2* genetic instruments for sepsis-related 28-day death; Figure S40: Phenome-wide associations of primary and functional *PPARG* and potassium-channel proxies; Figure S41: Associations of retained genetic instruments with smoking phenotypes; Figure S42: Individual *GPD2* variant associations with infection and sepsis phenotypes; Figure S43: Comparison of *SLC5A2* estimates from the six-SNP source set and the low-LD source subset; Table S1: Associations of primary and functional *PPARG* and *KCNJ11*/*ABCC8* proxies with 13 matched infection outcomes; Table S2: GWAS resource inventory and metadata for the matched infection outcomes; Table S3: GWAS resources considered for the type 2 diabetes positive control; Table S4: Genetic instrument metadata and approximate instrument strength; Table S5: Screening and retention of genetic instruments for putative metformin-related targets; Table S6: Estimators and sensitivity analyses by instrument panel; Table S7: Type 2 diabetes positive-control estimates for retained target proxies in a common GWAS.

## List of Abbreviations

ABCC8	ATP-binding cassette subfamily C member 8
AMPK	AMP-activated protein kinase
CI	Confidence interval
cis-eQTL	Cis-expression quantitative trait locus
COVID-19	Coronavirus disease 2019
DPP-4	Dipeptidyl peptidase-4
DNA	Deoxyribonucleic acid
ETFDH	Electron transfer flavoprotein dehydrogenase
FinnGen	Finnish Genomics project
GLP-1	Glucagon-like peptide-1
GLP1R	Glucagon-like peptide-1 receptor
GTEx	Genotype-Tissue Expression
GWAS	Genome-wide association study
HbA1c	Glycated hemoglobin
INSR	Insulin receptor
IV	Instrumental variable
IVW	Inverse-variance weighted
KATP	ATP-sensitive potassium
KCNJ11	Potassium inwardly rectifying channel subfamily J member 11
LD	Linkage disequilibrium
LRTI	Lower respiratory tract infection
LRTIs	Lower respiratory tract infections
MR	Mendelian randomization
MR-Egger	Mendelian randomization Egger regression
MR-PRESSO	Mendelian randomization pleiotropy residual sum and outlier
MR-RAPS	Mendelian randomization robust adjusted profile score
OR	Odds ratio
PEN2	Presenilin enhancer 2
PPARG	Peroxisome proliferator-activated receptor gamma
PRKAA1	Protein kinase AMP-activated catalytic subunit alpha 1
PRKAA2	Protein kinase AMP-activated catalytic subunit alpha 2
PRKAB1	Protein kinase AMP-activated non-catalytic subunit beta 1
PRKAB2	Protein kinase AMP-activated non-catalytic subunit beta 2
PRKAG1	Protein kinase AMP-activated non-catalytic subunit gamma 1
PRKAG2	Protein kinase AMP-activated non-catalytic subunit gamma 2
PRKAG3	Protein kinase AMP-activated non-catalytic subunit gamma 3
PSENEN	Presenilin enhancer, gamma-secretase subunit
SARS-CoV-2	Severe acute respiratory syndrome coronavirus 2
SE	Standard error
SGLT2	Sodium-glucose cotransporter 2
SLC5A2	Solute carrier family 5 member 2
SNP	Single-nucleotide polymorphism
STROBE-MR	Strengthening the Reporting of Observational Studies in Epidemiology Using Mendelian Randomization
T2DM	Type 2 diabetes mellitus
TZD	Thiazolidinedione
UK Biobank	United Kingdom Biobank
WHO	World Health Organization
