# Supplementary material for: Hypoglycemic Agent Targets and Susceptibility to Lower Respiratory Infection: A Pharmacogenomic Mendelian Randomization Study
Source: Genes (Basel). 2026 Oct 11;17(10):1258. doi: 10.3390/genes17101258 (PMC13704145; doi:10.3390/genes17101258)
Supplement: Supplementary file 1 [file genes-17-01258-s001.zip › genes-4584956-supplementary.pdf]

Supplementary Materials

Hypoglycemic Agent Targets and Susceptibility to Lower Respiratory Infection: A Pharmacogenomic Mendelian Randomization Study

Taikang Yao [1,2,†], Nan Wu [3,†], Yanqiu Ma [1,2], Zilu Wang [1,2], Beibei Liu [1,2], Ming Lu [1,2], Zhenchao Wu [1,2,4,*] and Ning Shen [1,2,4,*]

1 Department of Pulmonary and Critical Care Medicine, Peking University Third Hospital, No. 49 Huayuan North Road, Haidian District, Beijing 100191, China

2 Center for Infectious Diseases, Peking University Third Hospital, No. 49 Huayuan North Road, Haidian District, Beijing 100191, China

3 Department of Respiratory and Critical Care Medicine, Peking University People's Hospital, No. 11 Xizhimen South Street, Xicheng District, Beijing 100044, China

4 National Key Laboratory of Vascular Homeostasis and Remodeling, Peking University Health Science Center, No. 38 Xueyuan Road, Haidian District, Beijing 100191, China

* Correspondence: wuzhenchao@pku.edu.cn (Z.W.); puh3shenning@bjmu.edu.cn (N.S.);
Tel.: +86-10-82266655 (N.S.); Fax: +86-10-62017700 (N.S.)

† These authors contributed equally to this work.

Supplementary Figures S1–S43

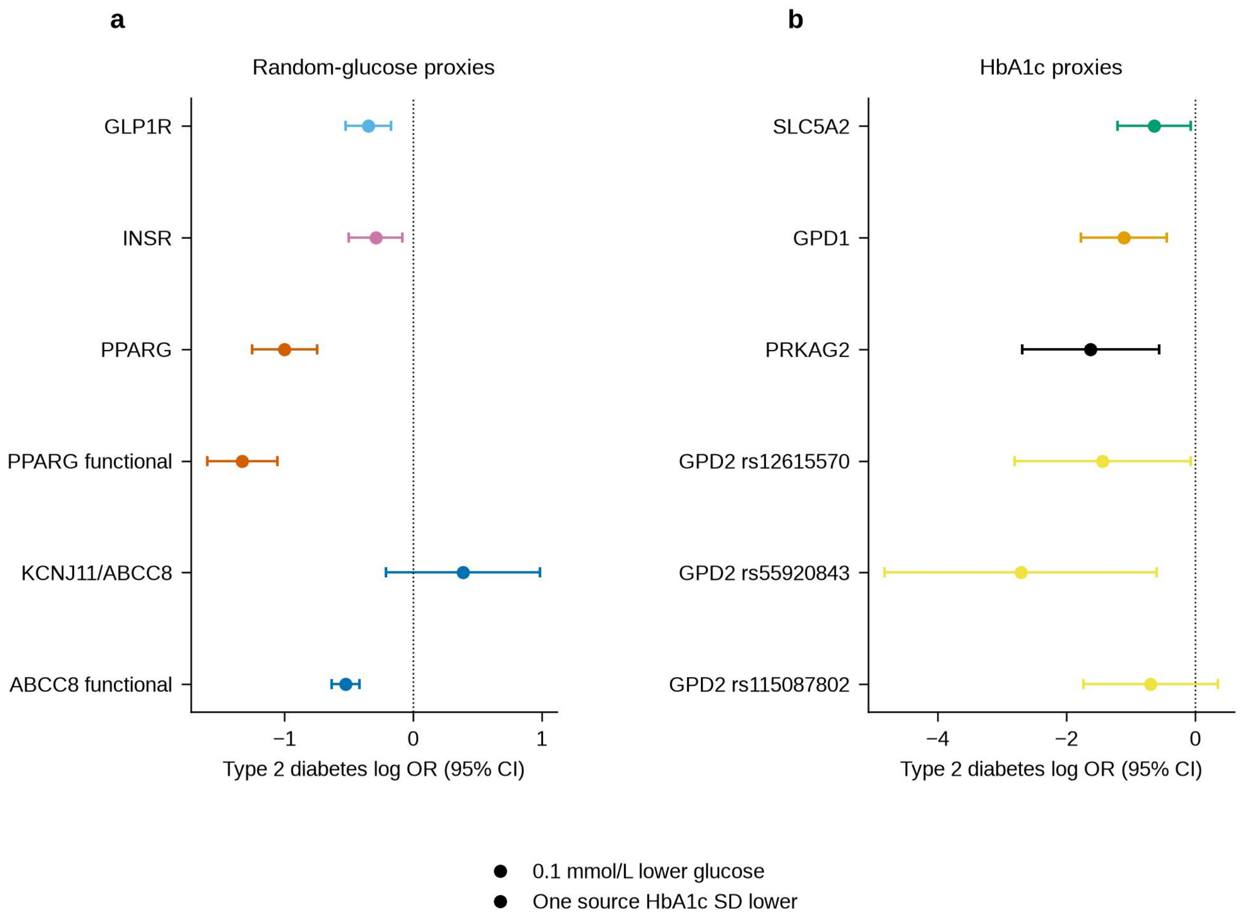

Figure S1. Type 2 diabetes positive-control associations for 12 target or variant groups. T2D associations were estimated using a common GWAS (ebi-a-GCST90018926; 38,841 cases and 451,248 controls). **(a)** Six random-glucose proxy groups, calibrated per 0.1 mmol/L lower random glucose: *GLP1R*, *INSR*, primary and functional *PPARG*, primary *KCNJ11*/*ABCC8*, and functional *ABCC8*. **(b)** Six HbA1c proxy groups, calibrated per one source SD lower HbA1c: *SLC5A2*, *GPD1*, *PRKAG2*, and three individual *GPD2* variants. The functional *PPARG* and *ABCC8* proxies are rs1801282 (Pro12Ala) and rs757110 (S1369A), respectively. Circles show calibrated log ORs, and horizontal bars show 95% CIs. The two-SNP channel and six-SNP *SLC5A2* panels were estimated by generalized IVW using signed, allele-aligned LD from 503 European participants in 1000 Genomes Phase 3, with multiplicative random-effects SEs; single-variant estimates used Wald ratios. Colors identify target groups. The vertical dotted lines mark no association (log OR = 0), and the separate panels retain their source exposure scales.

Abbreviations: *ABCC8*, ATP-binding cassette subfamily C member 8; CI, confidence interval; *GLP1R*, glucagon-like peptide 1 receptor; *GPD1*, glycerol-3-phosphate dehydrogenase 1; *GPD2*, glycerol-3-phosphate dehydrogenase 2; GWAS, genome-wide association study; HbA1c, glycated hemoglobin; *INSR*, insulin receptor; IVW, inverse-variance weighted; *KCNJ11*, potassium inwardly rectifying channel subfamily J member 11; LD, linkage disequilibrium; OR, odds ratio; *PPARG*, peroxisome proliferator-activated receptor gamma; *PRKAG2*, protein kinase AMP-activated non-catalytic subunit gamma 2; Pro12Ala, proline-to-alanine substitution at residue 12; S1369A, serine-to-alanine substitution at residue 1369; SD, standard deviation; SE, standard error; *SLC5A2*, solute carrier family 5 member 2; SNP, single-nucleotide polymorphism; T2D, type 2 diabetes.

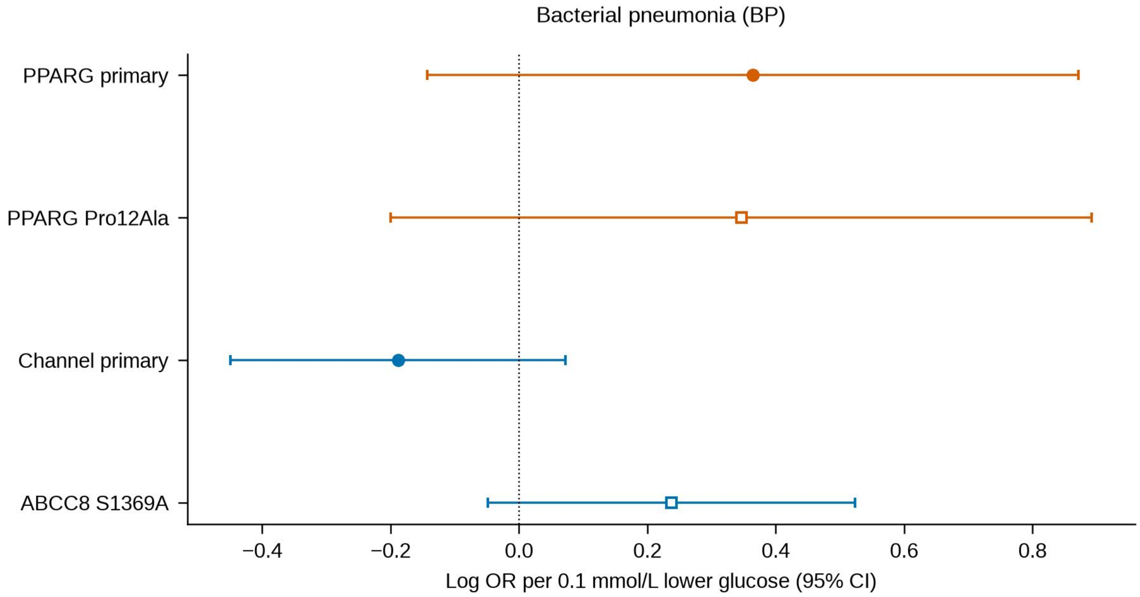

Figure S2. Associations of *PPARG* and *KCNJ11*/*ABCC8* proxies with bacterial pneumonia. The four rows show estimates for finn-b-J10_PNEUMOBACT: *PPARG* rs35240997, *PPARG* rs1801282 (Pro12Ala), the *KCNJ11*/*ABCC8* rs5219–rs739688 panel, and *ABCC8* rs757110 (S1369A). Filled circles indicate primary proxies, open squares indicate functional proxies, vermilion indicates *PPARG*, and blue indicates channel proxies. Points and horizontal bars represent calibrated log ORs and 95% CIs per 0.1 mmol/L lower random glucose. The two-SNP primary channel panel was estimated by generalized IVW using signed, allele-aligned LD from 503 European participants in 1000 Genomes Phase 3 and multiplicative random-effects SEs; single-variant estimates used Wald ratios. The vertical dotted line marks no association (log OR = 0). Two-sided p values and BH-adjusted q values for the 52 matched proxy comparisons are reported in Table S1.

Abbreviations: *ABCC8*, ATP-binding cassette subfamily C member 8; BH, Benjamini–Hochberg; BP, bacterial pneumonia; CI, confidence interval; IVW, inverse-variance weighted; *KCNJ11*, potassium inwardly rectifying channel subfamily J member 11; LD, linkage disequilibrium; OR, odds ratio; *PPARG*, peroxisome proliferator-activated receptor gamma; Pro12Ala, proline-to-alanine substitution at residue 12; S1369A, serine-to-alanine substitution at residue 1369; SE, standard error; SNP, single-nucleotide polymorphism.

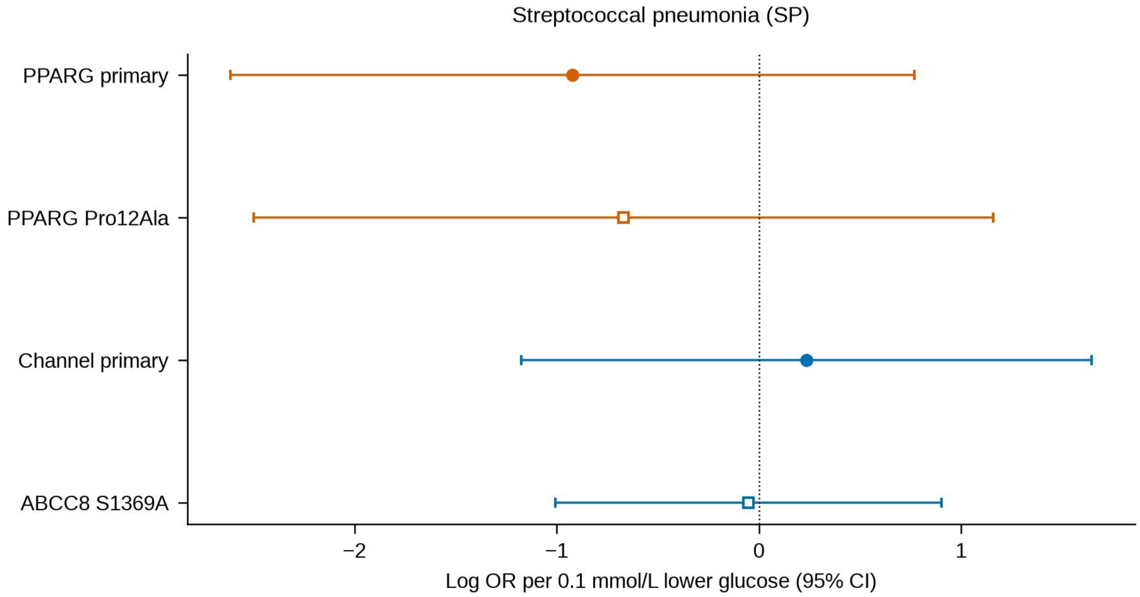

Figure S3. Associations of *PPARG* and *KCNJ11*/*ABCC8* proxies with pneumococcal pneumonia. The four rows show estimates for finn-b-J10_PNEUMOPNEUMO: *PPARG* rs35240997, *PPARG* rs1801282 (Pro12Ala), the *KCNJ11*/*ABCC8* rs5219–rs739688 panel, and *ABCC8* rs757110 (S1369A). Filled circles indicate primary proxies, open squares indicate functional proxies, vermilion indicates *PPARG*, and blue indicates channel proxies. Points and horizontal bars represent calibrated log ORs and 95% CIs per 0.1 mmol/L lower random glucose. The two-SNP primary channel panel was estimated by generalized IVW using signed, allele-aligned LD from 503 European participants in 1000 Genomes Phase 3 and multiplicative random-effects SEs; single-variant estimates used Wald ratios. The vertical dotted line marks no association (log OR = 0). Two-sided p values and BH-adjusted q values for the 52 matched proxy comparisons are reported in Table S1.

Abbreviations: *ABCC8*, ATP-binding cassette subfamily C member 8; BH, Benjamini–Hochberg; CI, confidence interval; IVW, inverse-variance weighted; *KCNJ11*, potassium inwardly rectifying channel subfamily J member 11; LD, linkage disequilibrium; OR, odds ratio; *PPARG*, peroxisome proliferator-activated receptor gamma; Pro12Ala, proline-to-alanine substitution at residue 12; S1369A, serine-to-alanine substitution at residue 1369; SE, standard error; SNP, single-nucleotide polymorphism; SP, pneumococcal pneumonia.

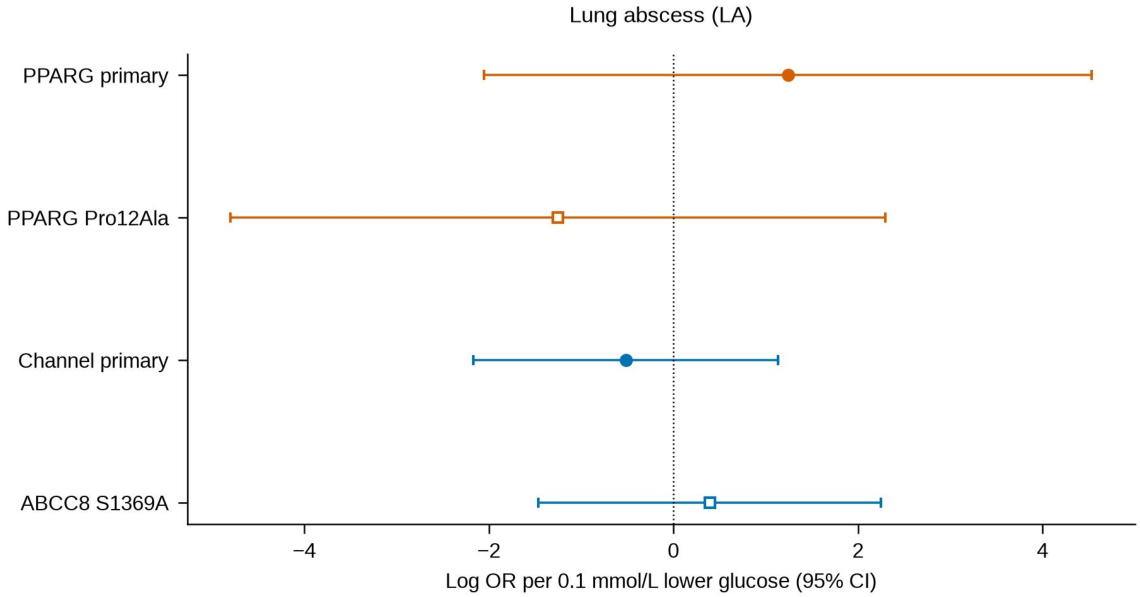

Figure S4. Associations of *PPARG* and *KCNJ11*/*ABCC8* proxies with lung abscess. The four rows show estimates for finn-b-J10_ABSCLUNGPNEUMO: *PPARG* rs35240997, *PPARG* rs1801282 (Pro12Ala), the *KCNJ11*/*ABCC8* rs5219–rs739688 panel, and *ABCC8* rs757110 (S1369A). Filled circles indicate primary proxies, open squares indicate functional proxies, vermilion indicates *PPARG*, and blue indicates channel proxies. Points and horizontal bars represent calibrated log ORs and 95% CIs per 0.1 mmol/L lower random glucose. The two-SNP primary channel panel was estimated by generalized IVW using signed, allele-aligned LD from 503 European participants in 1000 Genomes Phase 3 and multiplicative random-effects SEs; single-variant estimates used Wald ratios. The vertical dotted line marks no association (log OR = 0). Two-sided p values and BH-adjusted q values for the 52 matched proxy comparisons are reported in Table S1.

Abbreviations: *ABCC8*, ATP-binding cassette subfamily C member 8; BH, Benjamini–Hochberg; CI, confidence interval; IVW, inverse-variance weighted; *KCNJ11*, potassium inwardly rectifying channel subfamily J member 11; LA, lung abscess; LD, linkage disequilibrium; OR, odds ratio; *PPARG*, peroxisome proliferator-activated receptor gamma; Pro12Ala, proline-to-alanine substitution at residue 12; S1369A, serine-to-alanine substitution at residue 1369; SE, standard error; SNP, single-nucleotide polymorphism.

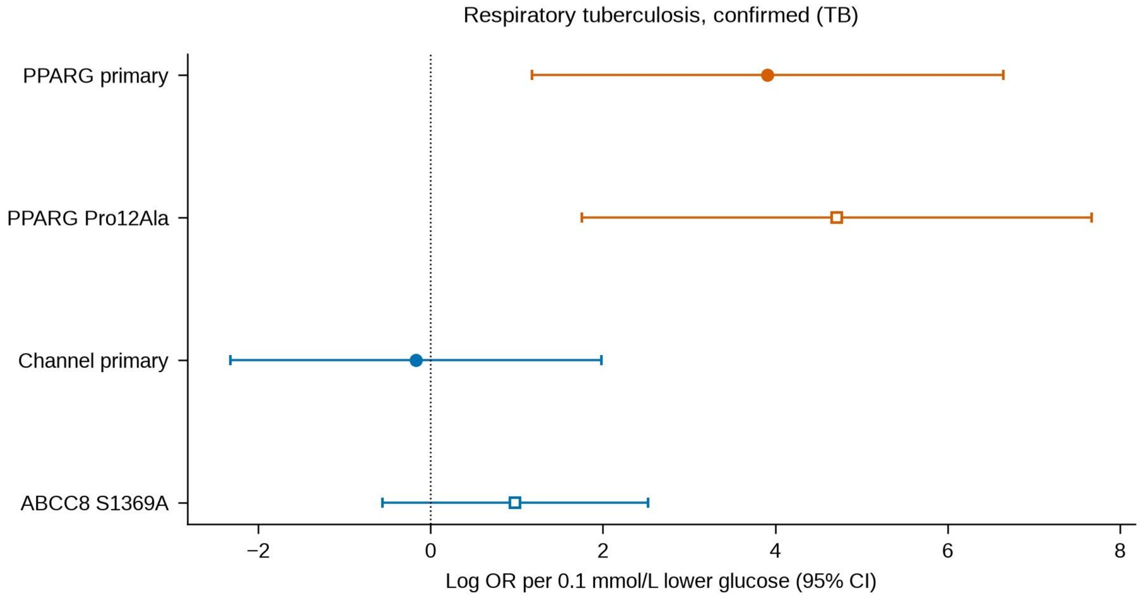

Figure S5. Associations of *PPARG* and *KCNJ11*/*ABCC8* proxies with confirmed respiratory tuberculosis. The four rows show estimates for finn-b-AB1_RESP_TUBERCU_CONF: *PPARG* rs35240997, *PPARG* rs1801282 (Pro12Ala), the *KCNJ11*/*ABCC8* rs5219–rs739688 panel, and *ABCC8* rs757110 (S1369A). Filled circles indicate primary proxies, open squares indicate functional proxies, vermilion indicates *PPARG*, and blue indicates channel proxies. Points and horizontal bars represent calibrated log ORs and 95% CIs per 0.1 mmol/L lower random glucose. The two-SNP primary channel panel was estimated by generalized IVW using signed, allele-aligned LD from 503 European participants in 1000 Genomes Phase 3 and multiplicative random-effects SEs; single-variant estimates used Wald ratios. The vertical dotted line marks no association (log OR = 0). Two-sided p values and BH-adjusted q values for the 52 matched proxy comparisons are reported in Table S1.

Abbreviations: *ABCC8*, ATP-binding cassette subfamily C member 8; BH, Benjamini–Hochberg; CI, confidence interval; IVW, inverse-variance weighted; *KCNJ11*, potassium inwardly rectifying channel subfamily J member 11; LD, linkage disequilibrium; OR, odds ratio; *PPARG*, peroxisome proliferator-activated receptor gamma; Pro12Ala, proline-to-alanine substitution at residue 12; S1369A, serine-to-alanine substitution at residue 1369; SE, standard error; SNP, single-nucleotide polymorphism; TB, confirmed respiratory tuberculosis.

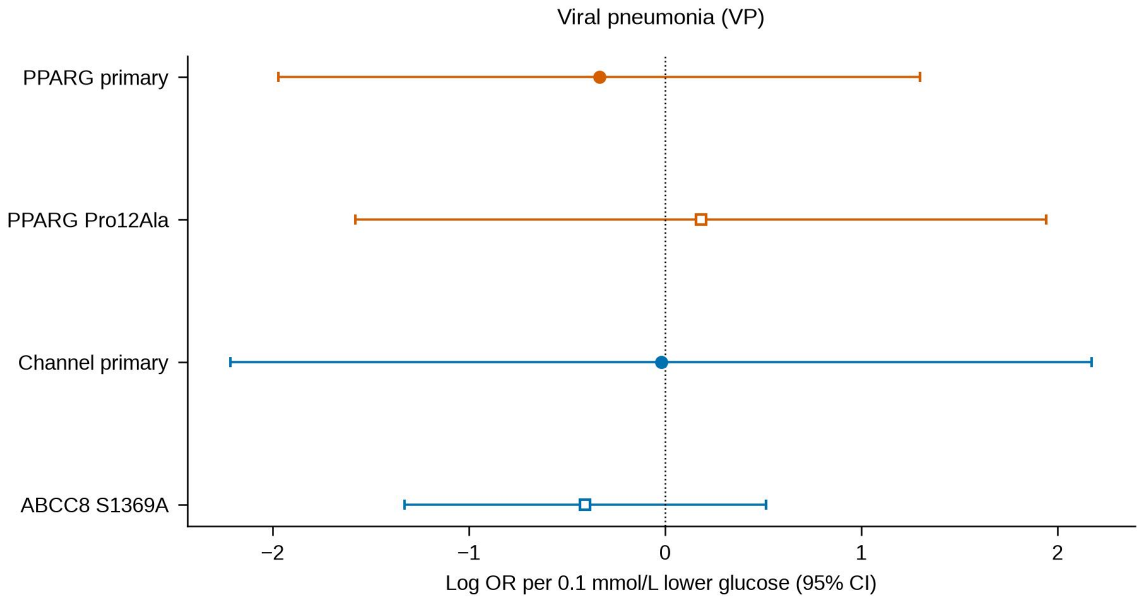

Figure S6. Associations of *PPARG* and *KCNJ11*/*ABCC8* proxies with viral pneumonia. The four rows show estimates for finn-b-J10_VIRALPNEUMO: *PPARG* rs35240997, *PPARG* rs1801282 (Pro12Ala), the *KCNJ11*/*ABCC8* rs5219–rs739688 panel, and *ABCC8* rs757110 (S1369A). Filled circles indicate primary proxies, open squares indicate functional proxies, vermilion indicates *PPARG*, and blue indicates channel proxies. Points and horizontal bars represent calibrated log ORs and 95% CIs per 0.1 mmol/L lower random glucose. The two-SNP primary channel panel was estimated by generalized IVW using signed, allele-aligned LD from 503 European participants in 1000 Genomes Phase 3 and multiplicative random-effects SEs; single-variant estimates used Wald ratios. The vertical dotted line marks no association (log OR = 0). Two-sided p values and BH-adjusted q values for the 52 matched proxy comparisons are reported in Table S1.

Abbreviations: *ABCC8*, ATP-binding cassette subfamily C member 8; BH, Benjamini–Hochberg; CI, confidence interval; IVW, inverse-variance weighted; *KCNJ11*, potassium inwardly rectifying channel subfamily J member 11; LD, linkage disequilibrium; OR, odds ratio; *PPARG*, peroxisome proliferator-activated receptor gamma; Pro12Ala, proline-to-alanine substitution at residue 12; S1369A, serine-to-alanine substitution at residue 1369; SE, standard error; SNP, single-nucleotide polymorphism; VP, viral pneumonia.

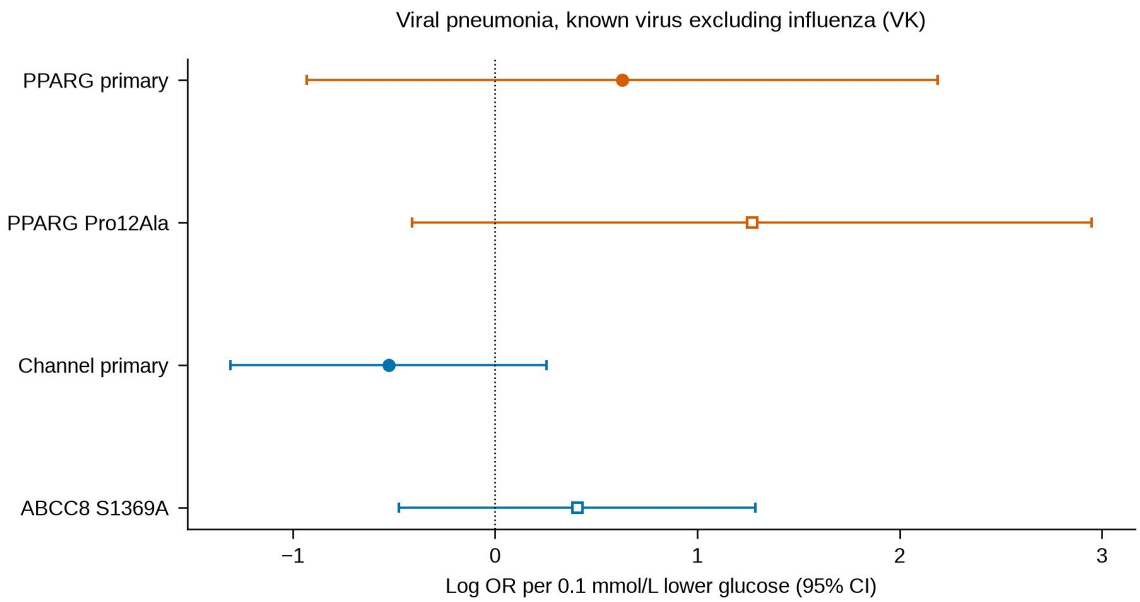

Figure S7. Associations of *PPARG* and *KCNJ11*/*ABCC8* proxies with viral pneumonia due to a known virus excluding influenza. The four rows show estimates for finn-b-VIRALPNEUMOKNOWN: *PPARG* rs35240997, *PPARG* rs1801282 (Pro12Ala), the *KCNJ11*/*ABCC8* rs5219–rs739688 panel, and *ABCC8* rs757110 (S1369A). Filled circles indicate primary proxies, open squares indicate functional proxies, vermilion indicates *PPARG*, and blue indicates channel proxies. Points and horizontal bars represent calibrated log ORs and 95% CIs per 0.1 mmol/L lower random glucose. The two-SNP primary channel panel was estimated by generalized IVW using signed, allele-aligned LD from 503 European participants in 1000 Genomes Phase 3 and multiplicative random-effects SEs; single-variant estimates used Wald ratios. The vertical dotted line marks no association (log OR = 0). Two-sided p values and BH-adjusted q values for the 52 matched proxy comparisons are reported in Table S1.

Abbreviations: *ABCC8*, ATP-binding cassette subfamily C member 8; BH, Benjamini–Hochberg; CI, confidence interval; IVW, inverse-variance weighted; *KCNJ11*, potassium inwardly rectifying channel subfamily J member 11; LD, linkage disequilibrium; OR, odds ratio; *PPARG*, peroxisome proliferator-activated receptor gamma; Pro12Ala, proline-to-alanine substitution at residue 12; S1369A, serine-to-alanine substitution at residue 1369; SE, standard error; SNP, single-nucleotide polymorphism; VK, pneumonia due to a known virus excluding influenza.

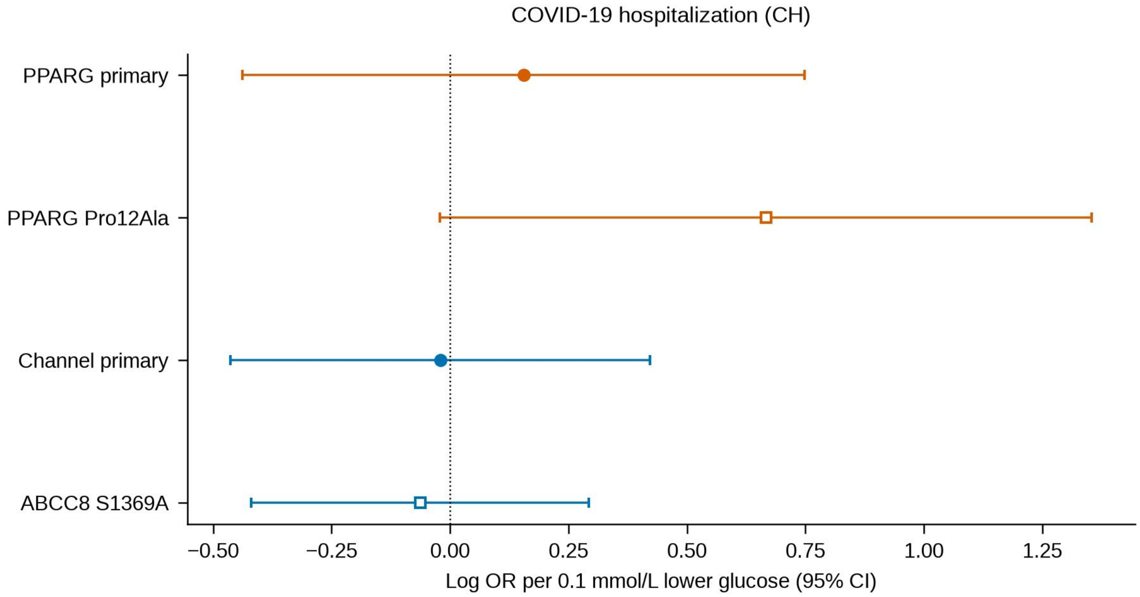

Figure S8. Associations of *PPARG* and *KCNJ11*/*ABCC8* proxies with COVID-19 hospitalization. The four rows show estimates for ebi-a-GCST011081: *PPARG* rs35240997, *PPARG* rs1801282 (Pro12Ala), the *KCNJ11*/*ABCC8* rs5219–rs739688 panel, and *ABCC8* rs757110 (S1369A). Filled circles indicate primary proxies, open squares indicate functional proxies, vermilion indicates *PPARG*, and blue indicates channel proxies. Points and horizontal bars represent calibrated log ORs and 95% CIs per 0.1 mmol/L lower random glucose. The two-SNP primary channel panel was estimated by generalized IVW using signed, allele-aligned LD from 503 European participants in 1000 Genomes Phase 3 and multiplicative random-effects SEs; single-variant estimates used Wald ratios. The vertical dotted line marks no association (log OR = 0). Two-sided p values and BH-adjusted q values for the 52 matched proxy comparisons are reported in Table S1.

Abbreviations: *ABCC8*, ATP-binding cassette subfamily C member 8; BH, Benjamini–Hochberg; CH, COVID-19 hospitalization; CI, confidence interval; COVID-19, coronavirus disease 2019; IVW, inverse-variance weighted; *KCNJ11*, potassium inwardly rectifying channel subfamily J member 11; LD, linkage disequilibrium; OR, odds ratio; *PPARG*, peroxisome proliferator-activated receptor gamma; Pro12Ala, proline-to-alanine substitution at residue 12; S1369A, serine-to-alanine substitution at residue 1369; SE, standard error; SNP, single-nucleotide polymorphism.

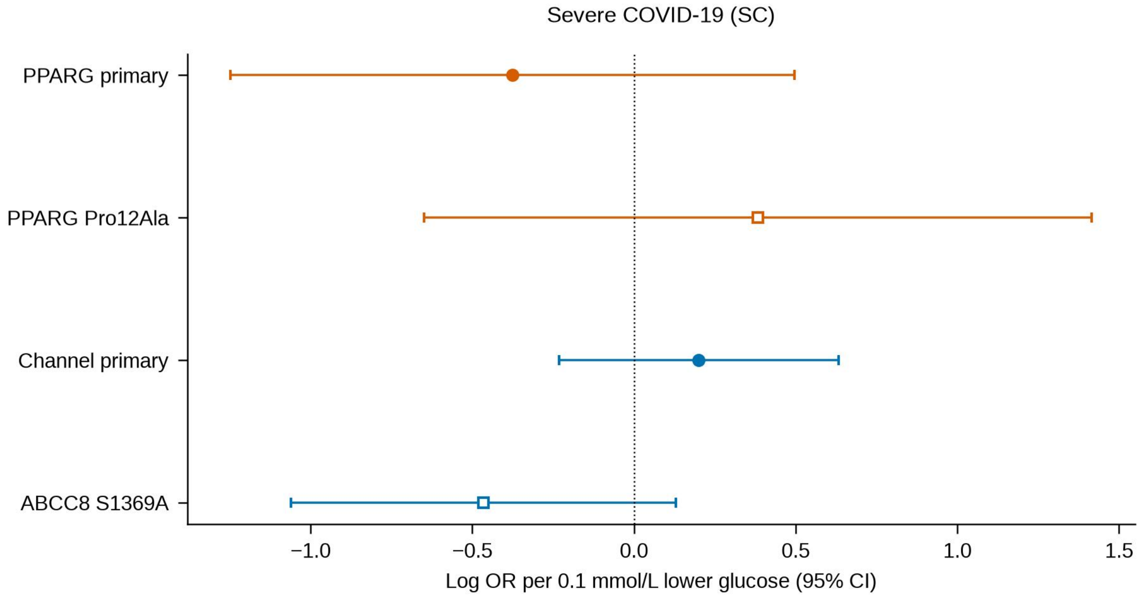

Figure S9. Associations of *PPARG* and *KCNJ11*/*ABCC8* proxies with severe COVID-19. The four rows show estimates for ebi-a-GCST011075: *PPARG* rs35240997, *PPARG* rs1801282 (Pro12Ala), the *KCNJ11*/*ABCC8* rs5219–rs739688 panel, and *ABCC8* rs757110 (S1369A). Filled circles indicate primary proxies, open squares indicate functional proxies, vermilion indicates *PPARG*, and blue indicates channel proxies. Points and horizontal bars represent calibrated log ORs and 95% CIs per 0.1 mmol/L lower random glucose. The two-SNP primary channel panel was estimated by generalized IVW using signed, allele-aligned LD from 503 European participants in 1000 Genomes Phase 3 and multiplicative random-effects SEs; single-variant estimates used Wald ratios. The vertical dotted line marks no association (log OR = 0). Two-sided p values and BH-adjusted q values for the 52 matched proxy comparisons are reported in Table S1.

Abbreviations: *ABCC8*, ATP-binding cassette subfamily C member 8; BH, Benjamini–Hochberg; CI, confidence interval; COVID-19, coronavirus disease 2019; IVW, inverse-variance weighted; *KCNJ11*, potassium inwardly rectifying channel subfamily J member 11; LD, linkage disequilibrium; OR, odds ratio; *PPARG*, peroxisome proliferator-activated receptor gamma; Pro12Ala, proline-to-alanine substitution at residue 12; S1369A, serine-to-alanine substitution at residue 1369; SC, severe COVID-19; SE, standard error; SNP, single-nucleotide polymorphism.

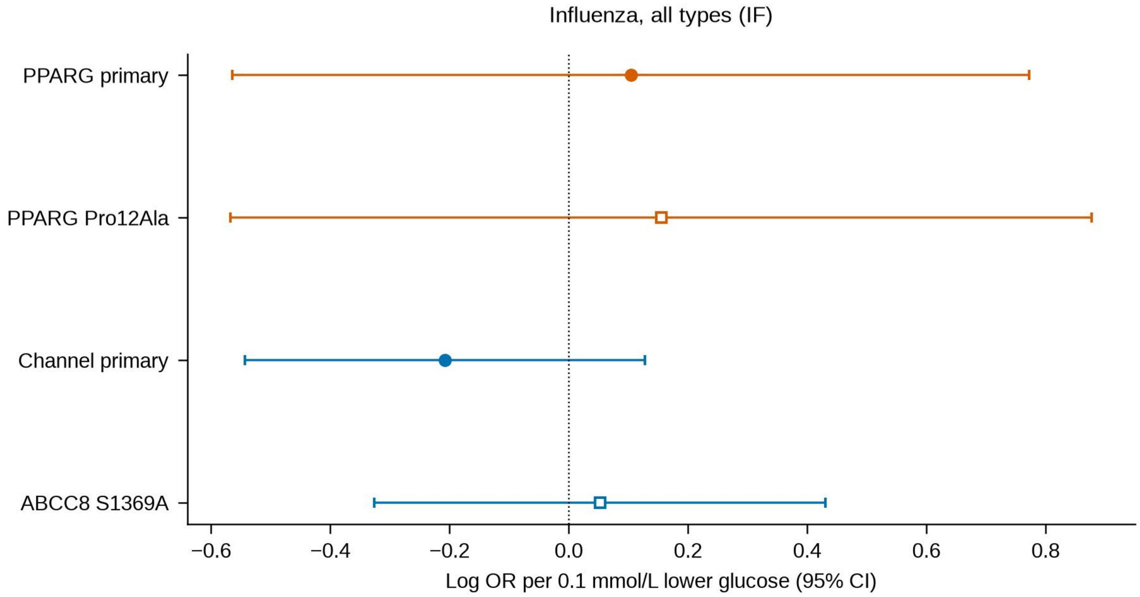

Figure S10. Associations of *PPARG* and *KCNJ11*/*ABCC8* proxies with influenza. The four rows show estimates for finn-b-J10_INFLUENZA: *PPARG* rs35240997, *PPARG* rs1801282 (Pro12Ala), the *KCNJ11*/*ABCC8* rs5219–rs739688 panel, and *ABCC8* rs757110 (S1369A). Filled circles indicate primary proxies, open squares indicate functional proxies, vermilion indicates *PPARG*, and blue indicates channel proxies. Points and horizontal bars represent calibrated log ORs and 95% CIs per 0.1 mmol/L lower random glucose. The two-SNP primary channel panel was estimated by generalized IVW using signed, allele-aligned LD from 503 European participants in 1000 Genomes Phase 3 and multiplicative random-effects SEs; single-variant estimates used Wald ratios. The vertical dotted line marks no association (log OR = 0). Two-sided p values and BH-adjusted q values for the 52 matched proxy comparisons are reported in Table S1.

Abbreviations: *ABCC8*, ATP-binding cassette subfamily C member 8; BH, Benjamini–Hochberg; CI, confidence interval; IF, influenza of all types; IVW, inverse-variance weighted; *KCNJ11*, potassium inwardly rectifying channel subfamily J member 11; LD, linkage disequilibrium; OR, odds ratio; *PPARG*, peroxisome proliferator-activated receptor gamma; Pro12Ala, proline-to-alanine substitution at residue 12; S1369A, serine-to-alanine substitution at residue 1369; SE, standard error; SNP, single-nucleotide polymorphism.

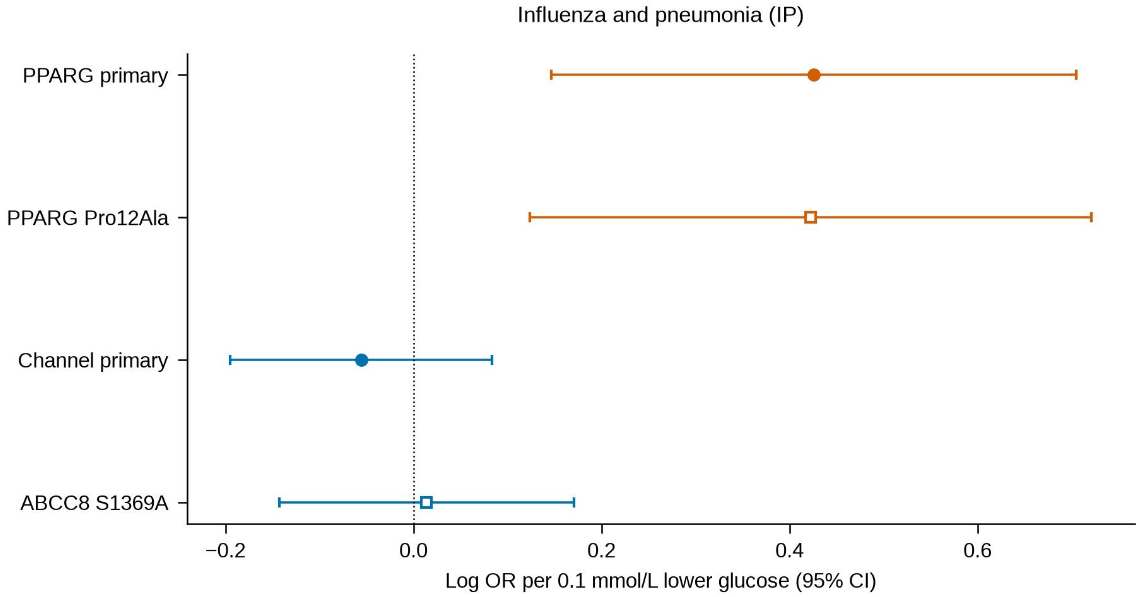

Figure S11. Associations of *PPARG* and *KCNJ11*/*ABCC8* proxies with influenza and pneumonia composite. The four rows show estimates for finn-b-J10_INFLUPNEU: *PPARG* rs35240997, *PPARG* rs1801282 (Pro12Ala), the *KCNJ11*/*ABCC8* rs5219–rs739688 panel, and *ABCC8* rs757110 (S1369A). Filled circles indicate primary proxies, open squares indicate functional proxies, vermilion indicates *PPARG*, and blue indicates channel proxies. Points and horizontal bars represent calibrated log ORs and 95% CIs per 0.1 mmol/L lower random glucose. The two-SNP primary channel panel was estimated by generalized IVW using signed, allele-aligned LD from 503 European participants in 1000 Genomes Phase 3 and multiplicative random-effects SEs; single-variant estimates used Wald ratios. The vertical dotted line marks no association (log OR = 0). Two-sided p values and BH-adjusted q values for the 52 matched proxy comparisons are reported in Table S1.

Abbreviations: *ABCC8*, ATP-binding cassette subfamily C member 8; BH, Benjamini–Hochberg; CI, confidence interval; IP, influenza and pneumonia composite; IVW, inverse-variance weighted; *KCNJ11*, potassium inwardly rectifying channel subfamily J member 11; LD, linkage disequilibrium; OR, odds ratio; *PPARG*, peroxisome proliferator-activated receptor gamma; Pro12Ala, proline-to-alanine substitution at residue 12; S1369A, serine-to-alanine substitution at residue 1369; SE, standard error; SNP, single-nucleotide polymorphism.

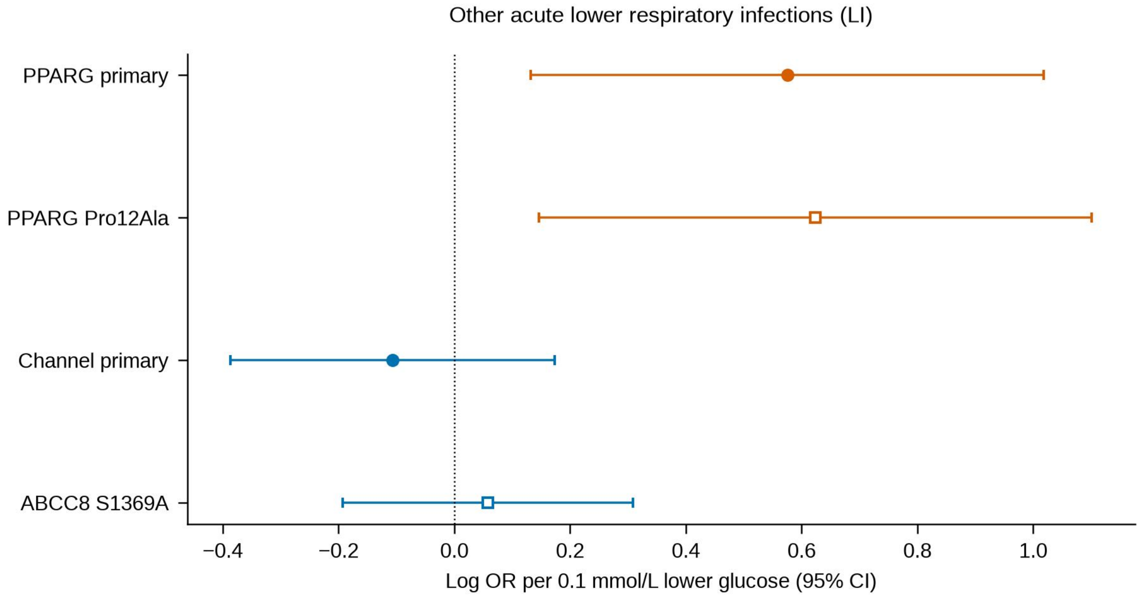

Figure S12. Associations of *PPARG* and *KCNJ11*/*ABCC8* proxies with other acute lower respiratory infections. The four rows show estimates for finn-b-J10_LOWERINF: *PPARG* rs35240997, *PPARG* rs1801282 (Pro12Ala), the *KCNJ11*/*ABCC8* rs5219–rs739688 panel, and *ABCC8* rs757110 (S1369A). Filled circles indicate primary proxies, open squares indicate functional proxies, vermilion indicates *PPARG*, and blue indicates channel proxies. Points and horizontal bars represent calibrated log ORs and 95% CIs per 0.1 mmol/L lower random glucose. The two-SNP primary channel panel was estimated by generalized IVW using signed, allele-aligned LD from 503 European participants in 1000 Genomes Phase 3 and multiplicative random-effects SEs; single-variant estimates used Wald ratios. The vertical dotted line marks no association (log OR = 0). Two-sided p values and BH-adjusted q values for the 52 matched proxy comparisons are reported in Table S1.

Abbreviations: *ABCC8*, ATP-binding cassette subfamily C member 8; BH, Benjamini–Hochberg; CI, confidence interval; IVW, inverse-variance weighted; *KCNJ11*, potassium inwardly rectifying channel subfamily J member 11; LD, linkage disequilibrium; LI, other acute lower respiratory infections; OR, odds ratio; *PPARG*, peroxisome proliferator-activated receptor gamma; Pro12Ala, proline-to-alanine substitution at residue 12; S1369A, serine-to-alanine substitution at residue 1369; SE, standard error; SNP, single-nucleotide polymorphism.

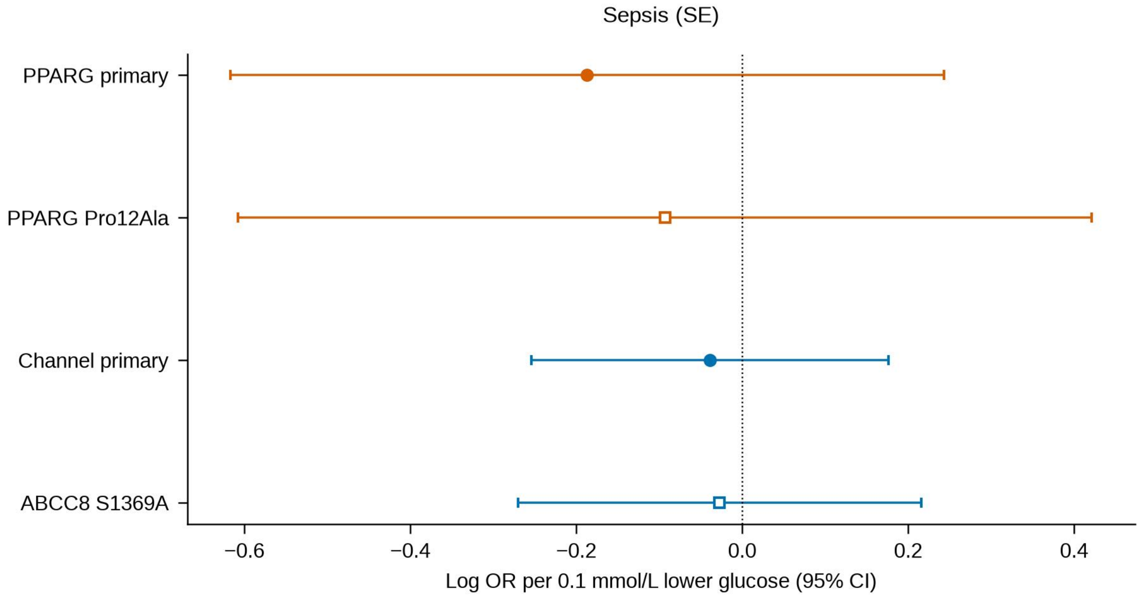

Figure S13. Associations of *PPARG* and *KCNJ11*/*ABCC8* proxies with sepsis. The four rows show estimates for ieu-b-4980: *PPARG* rs35240997, *PPARG* rs1801282 (Pro12Ala), the *KCNJ11*/*ABCC8* rs5219–rs739688 panel, and *ABCC8* rs757110 (S1369A). Filled circles indicate primary proxies, open squares indicate functional proxies, vermilion indicates *PPARG*, and blue indicates channel proxies. Points and horizontal bars represent calibrated log ORs and 95% CIs per 0.1 mmol/L lower random glucose. The two-SNP primary channel panel was estimated by generalized IVW using signed, allele-aligned LD from 503 European participants in 1000 Genomes Phase 3 and multiplicative random-effects SEs; single-variant estimates used Wald ratios. The vertical dotted line marks no association (log OR = 0). Two-sided p values and BH-adjusted q values for the 52 matched proxy comparisons are reported in Table S1.

Abbreviations: *ABCC8*, ATP-binding cassette subfamily C member 8; BH, Benjamini–Hochberg; CI, confidence interval; IVW, inverse-variance weighted; *KCNJ11*, potassium inwardly rectifying channel subfamily J member 11; LD, linkage disequilibrium; OR, odds ratio; *PPARG*, peroxisome proliferator-activated receptor gamma; Pro12Ala, proline-to-alanine substitution at residue 12; S1369A, serine-to-alanine substitution at residue 1369; SE, standard error (statistical quantity) or sepsis (phenotype code); SNP, single-nucleotide polymorphism.

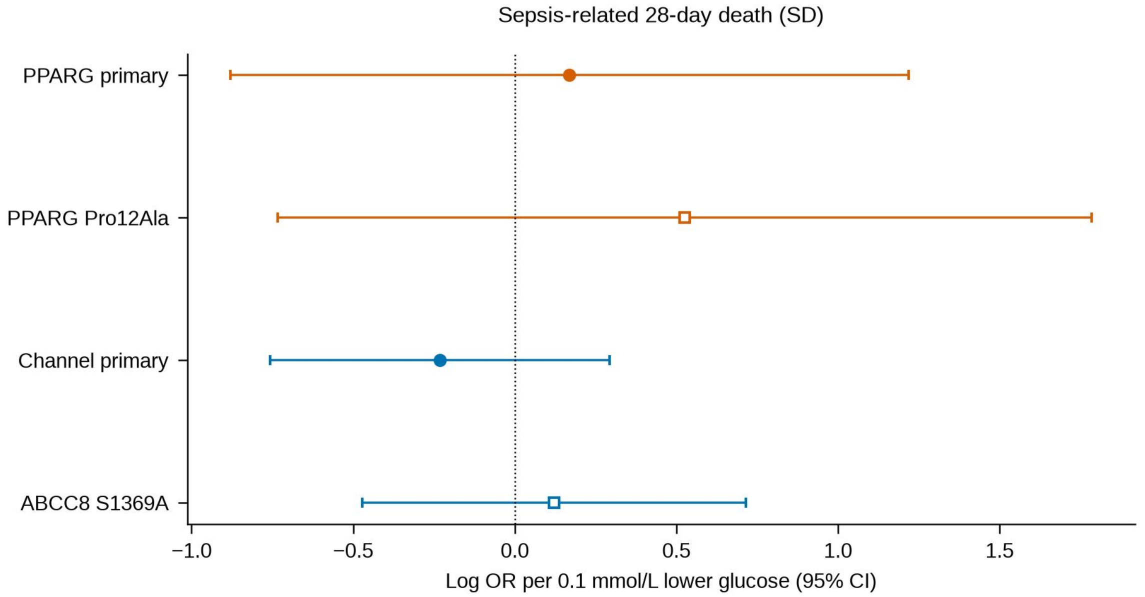

Figure S14. Associations of *PPARG* and *KCNJ11*/*ABCC8* proxies with sepsis-related 28-day death. The four rows show estimates for ieu-b-5086: *PPARG* rs35240997, *PPARG* rs1801282 (Pro12Ala), the *KCNJ11*/*ABCC8* rs5219–rs739688 panel, and *ABCC8* rs757110 (S1369A). Filled circles indicate primary proxies, open squares indicate functional proxies, vermilion indicates *PPARG*, and blue indicates channel proxies. Points and horizontal bars represent calibrated log ORs and 95% CIs per 0.1 mmol/L lower random glucose. The two-SNP primary channel panel was estimated by generalized IVW using signed, allele-aligned LD from 503 European participants in 1000 Genomes Phase 3 and multiplicative random-effects SEs; single-variant estimates used Wald ratios. The vertical dotted line marks no association (log OR = 0). Two-sided p values and BH-adjusted q values for the 52 matched proxy comparisons are reported in Table S1. The sepsis-related death endpoint uses population controls.

Abbreviations: *ABCC8*, ATP-binding cassette subfamily C member 8; BH, Benjamini–Hochberg; CI, confidence interval; IVW, inverse-variance weighted; *KCNJ11*, potassium inwardly rectifying channel subfamily J member 11; LD, linkage disequilibrium; OR, odds ratio; *PPARG*, peroxisome proliferator-activated receptor gamma; Pro12Ala, proline-to-alanine substitution at residue 12; S1369A, serine-to-alanine substitution at residue 1369; SD, sepsis-related 28-day death (phenotype code); SE, standard error; SNP, single-nucleotide polymorphism.

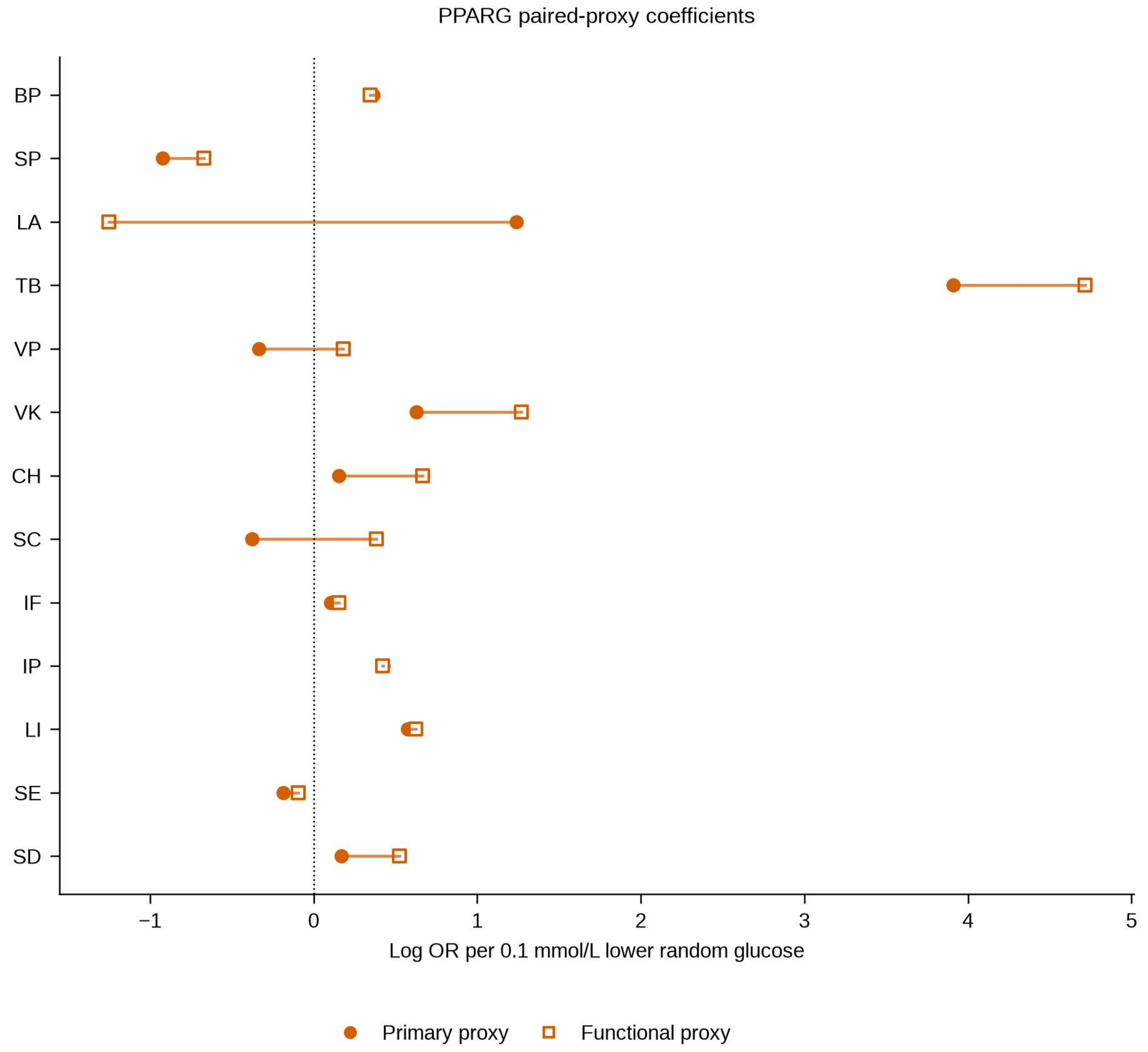

Figure S15. Paired *PPARG* proxy estimates across 13 infection and sepsis phenotypes. For each phenotype, filled vermilion circles show the primary *PPARG* rs35240997 estimate, and open vermilion squares show the functional rs1801282 (Pro12Ala) estimate from the same outcome GWAS accession. Single-variant associations were calculated using Wald ratios and expressed as log ORs per 0.1 mmol/L lower random glucose. Horizontal segments connect the paired point estimates; their 95% CIs are shown in Figures S2–S14. The vertical dotted line marks no association (log OR = 0).

Abbreviations: BP, bacterial pneumonia; CH, COVID-19 hospitalization; CI, confidence interval; COVID-19, coronavirus disease 2019; GWAS, genome-wide association study; IF, influenza of all types; IP, influenza and pneumonia composite; LA, lung abscess; LI, other acute lower respiratory infections; OR, odds ratio; *PPARG*, peroxisome proliferator-activated receptor gamma; Pro12Ala, proline-to-alanine substitution at residue 12; SC, severe COVID-19; SD, sepsis-related 28-day death (phenotype code); SE, sepsis (phenotype code); SP, pneumococcal pneumonia; TB, confirmed respiratory tuberculosis; VK, pneumonia due to a known virus excluding influenza; VP, viral pneumonia.

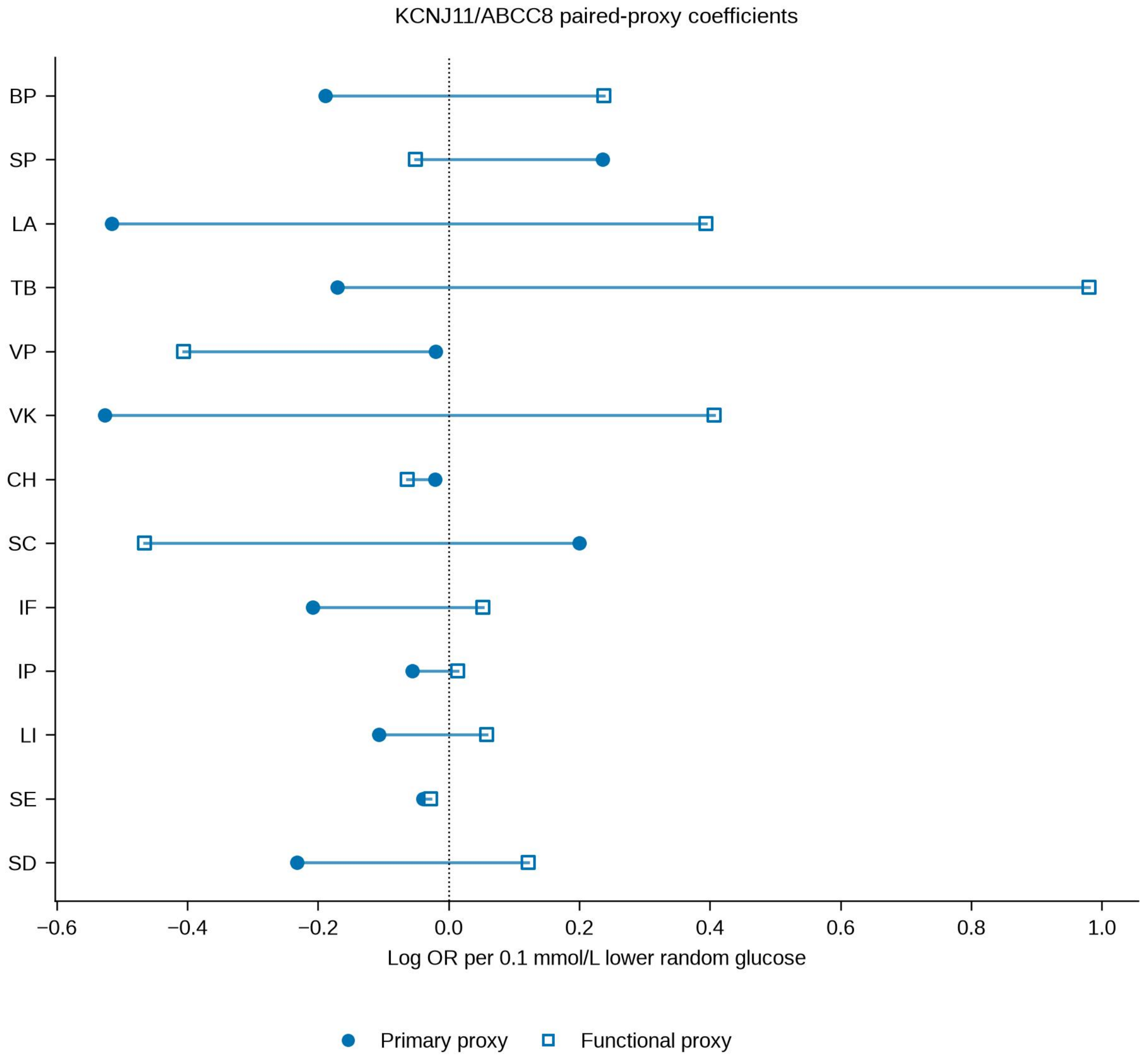

Figure S16. Paired *KCNJ11*/*ABCC8* proxy estimates across 13 infection and sepsis phenotypes. For each phenotype, filled blue circles show the primary *KCNJ11*/*ABCC8* rs5219–rs739688 panel estimate, and open blue squares show the functional *ABCC8* rs757110 (S1369A) estimate from the same outcome GWAS accession. Estimates are expressed as log ORs per 0.1 mmol/L lower random glucose. The primary panel was estimated by generalized IVW using signed, allele-aligned European reference LD; the functional estimate used a single-variant Wald ratio. Horizontal segments connect the paired point estimates; their 95% CIs are shown in Figures S2–S14. The vertical dotted line marks no association (log OR = 0).

Abbreviations: *ABCC8*, ATP-binding cassette subfamily C member 8; BP, bacterial pneumonia; CH, COVID-19 hospitalization; CI, confidence interval; COVID-19, coronavirus disease 2019; GWAS, genome-wide association study; IF, influenza of all types; IP, influenza and pneumonia composite; IVW, inverse-variance weighted; *KCNJ11*, potassium inwardly rectifying channel subfamily J member 11; LA, lung abscess; LD, linkage disequilibrium; LI, other acute lower respiratory infections; OR, odds ratio; S1369A, serine-to-alanine substitution at residue 1369; SC, severe COVID-19; SD, sepsis-related 28-day death (phenotype code); SE, sepsis (phenotype code); SP, pneumococcal pneumonia; TB, confirmed respiratory tuberculosis; VK, pneumonia due to a known virus excluding influenza; VP, viral pneumonia.

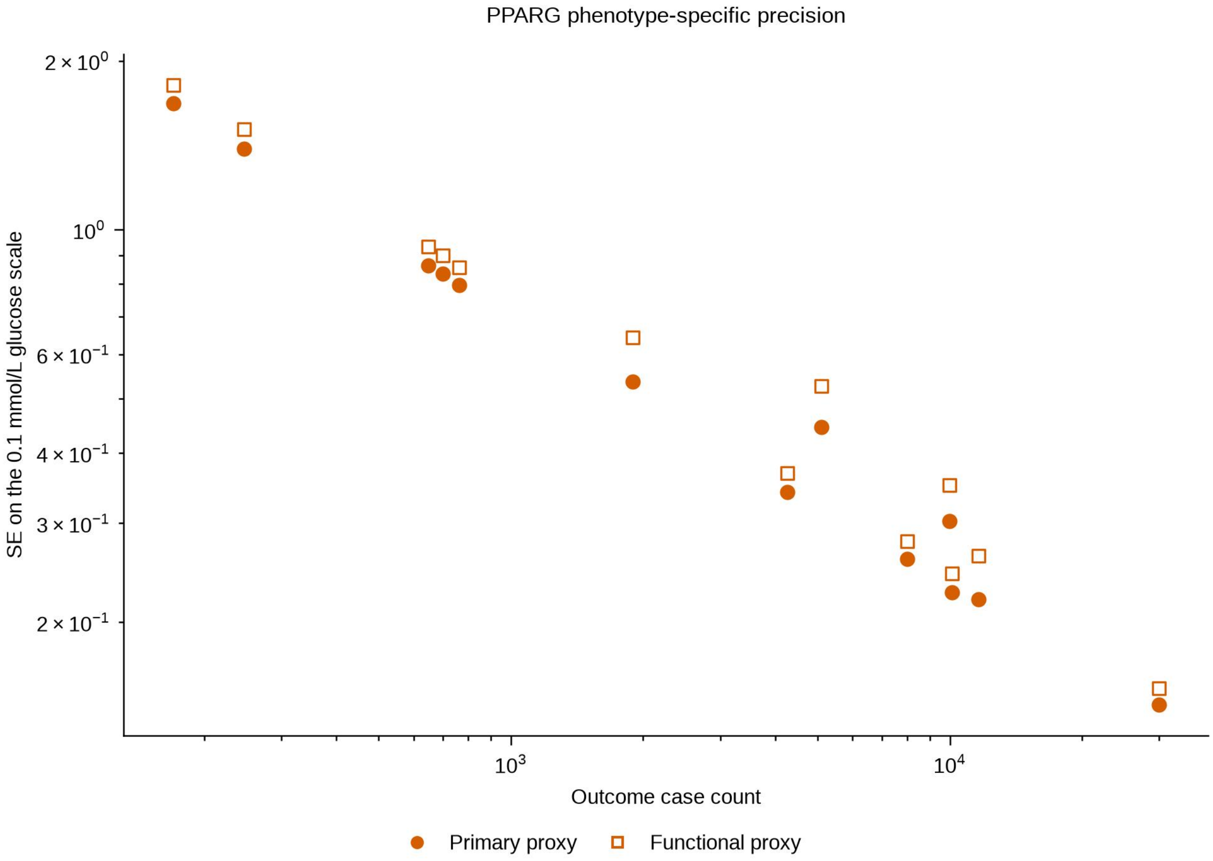

Figure S17. Outcome case counts and standard errors of *PPARG* proxy estimates. Each of the 13 infection and sepsis outcome GWAS contributes one point for the primary *PPARG* rs35240997 proxy (filled vermilion circle) and one for the functional rs1801282 (Pro12Ala) proxy (open vermilion square). Points are plotted by the outcome case count and the SE of the corresponding single-variant Wald estimate. SEs are expressed on the log OR scale per 0.1 mmol/L lower random glucose. Both axes use logarithmic scales.

Abbreviations: GWAS, genome-wide association study; OR, odds ratio; *PPARG*, peroxisome proliferator-activated receptor gamma; Pro12Ala, proline-to-alanine substitution at residue 12; SE, standard error.

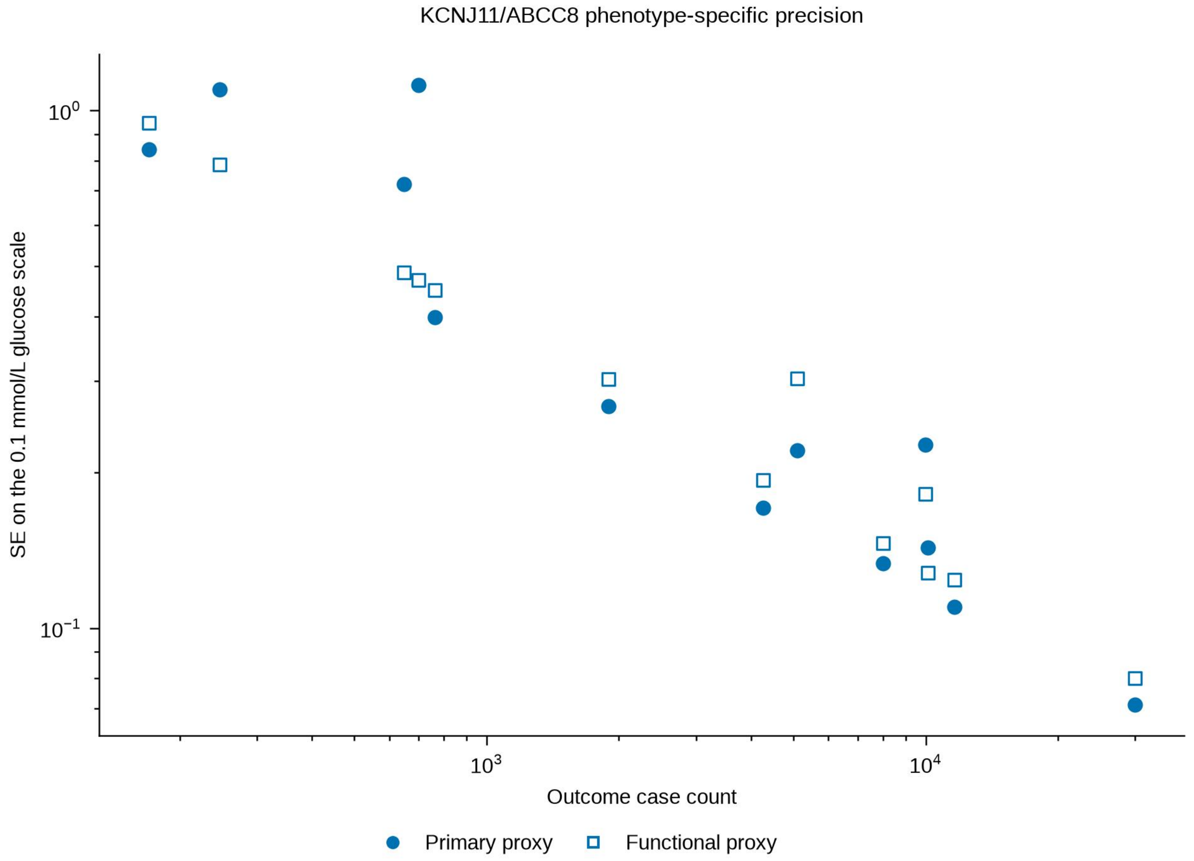

Figure S18. Outcome case counts and standard errors of *KCNJ11*/*ABCC8* proxy estimates. Each of the 13 infection and sepsis outcome GWAS contributes one point for the primary *KCNJ11*/*ABCC8* rs5219–rs739688 panel (filled blue circle) and one for the functional *ABCC8* rs757110 (S1369A) proxy (open blue square). Points are plotted by the outcome case count and the SE of the corresponding log OR per 0.1 mmol/L lower random glucose. Primary-panel SEs were obtained from generalized IVW using signed, allele-aligned European reference LD and multiplicative random-effects dispersion; the functional estimates used single-variant Wald ratios. Both axes use logarithmic scales.

Abbreviations: *ABCC8*, ATP-binding cassette subfamily C member 8; GWAS, genome-wide association study; IVW, inverse-variance weighted; *KCNJ11*, potassium inwardly rectifying channel subfamily J member 11; LD, linkage disequilibrium; OR, odds ratio; S1369A, serine-to-alanine substitution at residue 1369; SE, standard error.

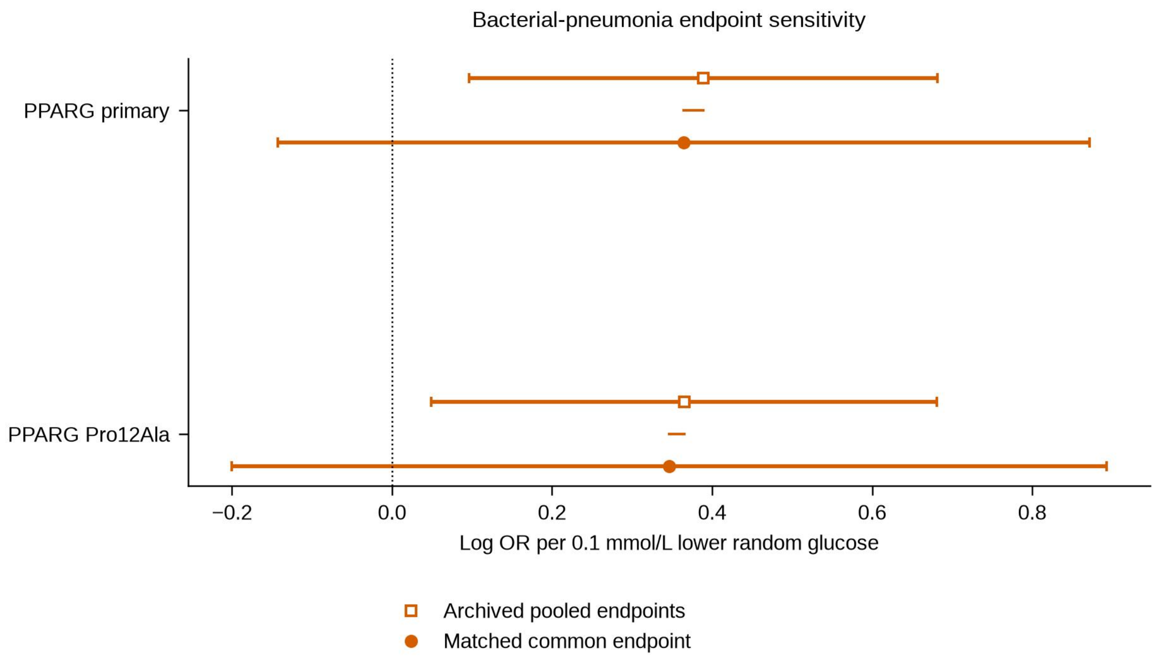

Figure S19. Sensitivity of *PPARG* bacterial pneumonia estimates to endpoint selection. The two rows compare the primary *PPARG* rs35240997 proxy and the functional rs1801282 (Pro12Ala) proxy. Open squares show archived estimates pooling three related bacterial pneumonia accessions (finn-b-J10_PNEUMOBACT, finn-b-J10_PNEUMOBACTEROTH, and finn-b-PNEUMOBACTKNOWN), and filled circles show re-estimated associations for the common endpoint finn-b-J10_PNEUMOBACT. Points and horizontal bars represent log ORs and 95% CIs per 0.1 mmol/L lower random glucose. Archived coefficients and SEs were multiplied by 0.1 to place them on this scale. Short horizontal segments between each pair span the two point estimates. The vertical dotted line marks no association (log OR = 0).

Abbreviations: CI, confidence interval; OR, odds ratio; *PPARG*, peroxisome proliferator-activated receptor gamma; Pro12Ala, proline-to-alanine substitution at residue 12; SE, standard error.

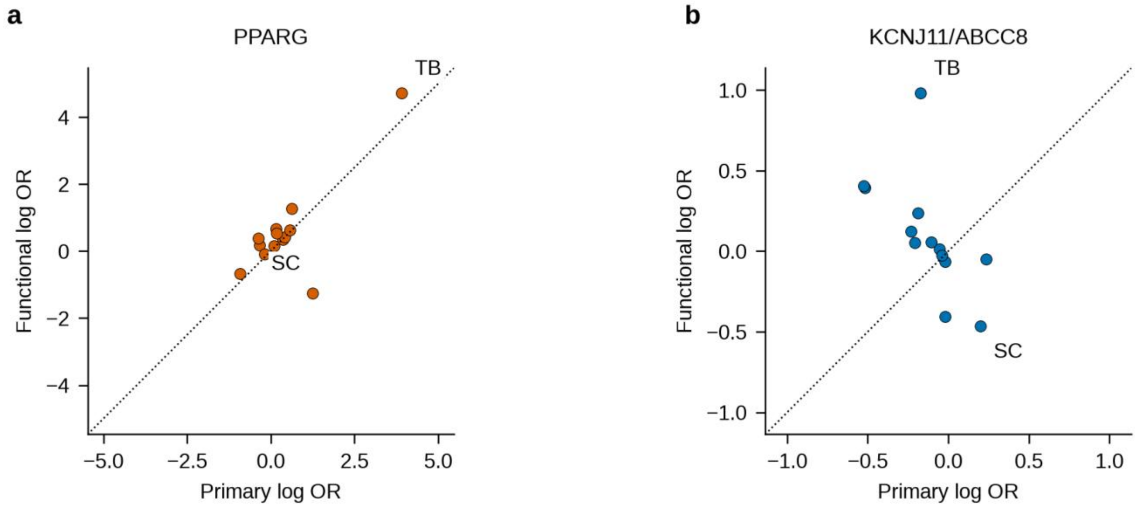

Figure S20. Directional agreement between primary and functional proxies across matched outcomes. **(a)** *PPARG* rs35240997 (primary) versus rs1801282 (Pro12Ala; functional), shown in vermilion. **(b)** The *KCNJ11*/*ABCC8* rs5219–rs739688 panel (primary) versus *ABCC8* rs757110 (S1369A; functional), shown in blue. Each point represents one of 13 infection and sepsis phenotypes, with the primary estimate on the horizontal axis and the functional estimate on the vertical axis. Matched estimates use the same outcome GWAS accession and are expressed as log ORs per 0.1 mmol/L lower random glucose. Dotted diagonal lines indicate equal coefficients. The primary channel panel was estimated by generalized IVW using signed, allele-aligned European reference LD; single-variant estimates used Wald ratios. The paired coefficients have the same sign for 10 of 13 *PPARG* comparisons and 3 of 13 channel comparisons. TB and SC identify the labeled phenotypes; corresponding 95% CIs are shown in Figures S2–S14.

Abbreviations: *ABCC8*, ATP-binding cassette subfamily C member 8; CI, confidence interval; COVID-19, coronavirus disease 2019; GWAS, genome-wide association study; IVW, inverse-variance weighted; *KCNJ11*, potassium inwardly rectifying channel subfamily J member 11; LD, linkage disequilibrium; OR, odds ratio; *PPARG*, peroxisome proliferator-activated receptor gamma; Pro12Ala, proline-to-alanine substitution at residue 12; S1369A, serine-to-alanine substitution at residue 1369; SC, severe COVID-19; TB, confirmed respiratory tuberculosis.

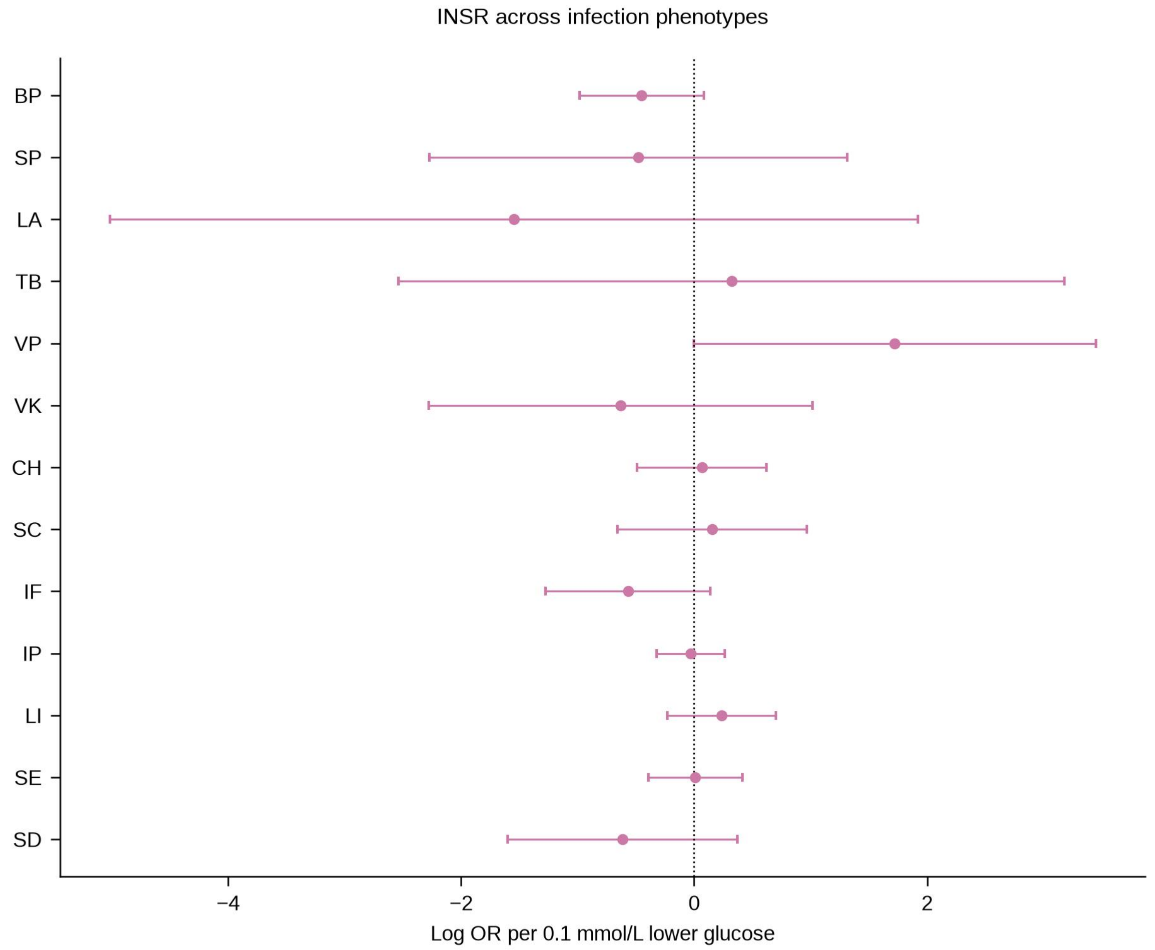

Figure S21. Associations of the *INSR* genetic proxy with infection and sepsis phenotypes. Pink circles show single-variant Wald-ratio estimates for the *INSR* proxy rs8103934 across the 13 matched infection and sepsis outcomes. Estimates are log ORs per 0.1 mmol/L decrease in random glucose, after alignment to the exposure effect allele. Horizontal bars indicate 95% CIs, and the dotted vertical line marks the null value of zero.

Abbreviations: BP, bacterial pneumonia; CH, COVID-19 hospitalization; CI, confidence interval; COVID-19, coronavirus disease 2019; IF, influenza of all types; *INSR*, insulin receptor; IP, influenza and pneumonia composite; LA, lung abscess; LI, other acute lower respiratory infections; OR, odds ratio; SC, severe COVID-19; SD, sepsis-related 28-day death (phenotype code); SE, sepsis (phenotype code); SP, pneumococcal pneumonia; TB, confirmed respiratory tuberculosis; VK, pneumonia due to a known virus excluding influenza; VP, viral pneumonia.

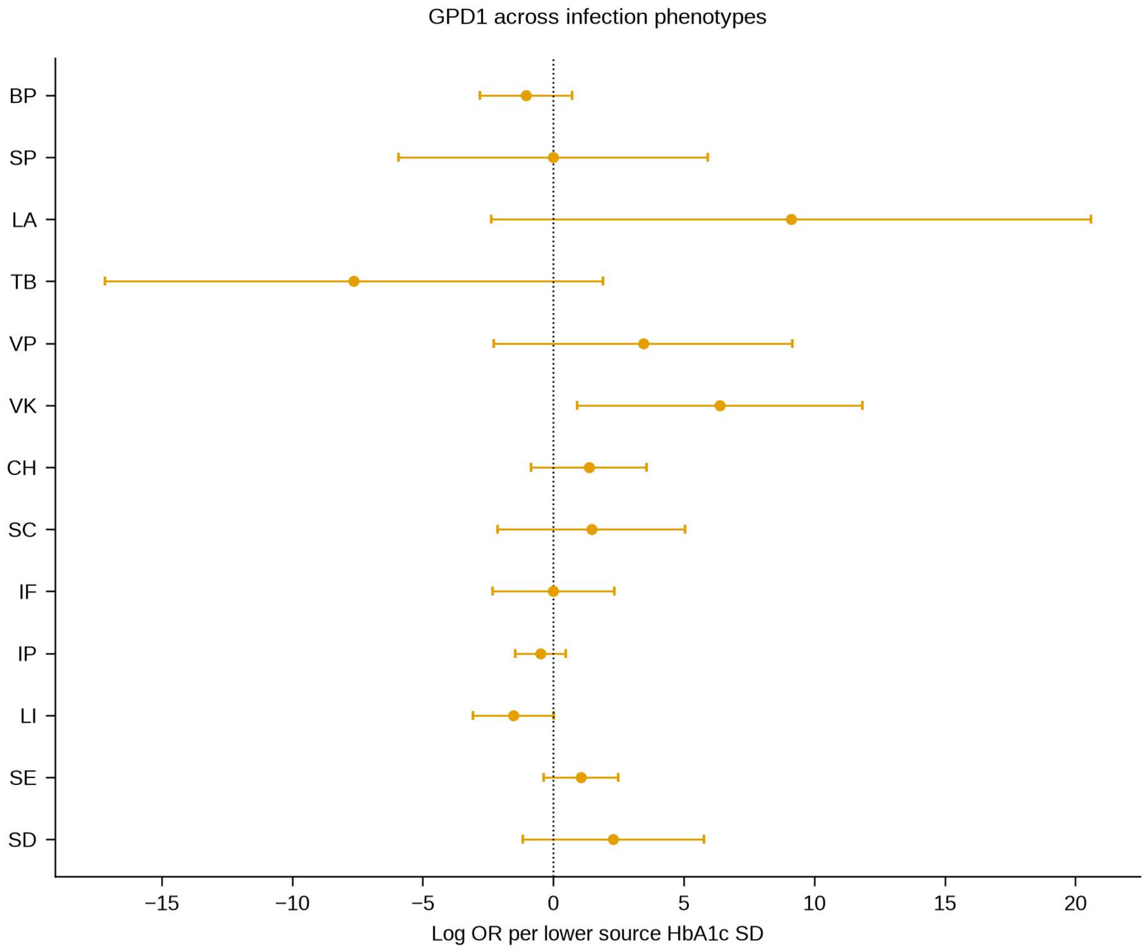

Figure S22. Associations of the *GPD1* genetic proxy with infection and sepsis phenotypes. Orange circles show single-variant Wald-ratio estimates for the *GPD1* proxy rs12581493 across the 13 matched infection and sepsis outcomes. Estimates are log ORs per one source SD lower HbA1c, after alignment to the exposure effect allele. Horizontal bars indicate 95% CIs, and the dotted vertical line marks the null value of zero.

Abbreviations: BP, bacterial pneumonia; CH, COVID-19 hospitalization; CI, confidence interval; COVID-19, coronavirus disease 2019; *GPD1*, glycerol-3-phosphate dehydrogenase 1; HbA1c, glycated hemoglobin; IF, influenza of all types; IP, influenza and pneumonia composite; LA, lung abscess; LI, other acute lower respiratory infections; OR, odds ratio; SC, severe COVID-19; SD, standard deviation (exposure unit) or sepsis-related 28-day death (phenotype code); SE, sepsis (phenotype code); SP, pneumococcal pneumonia; TB, confirmed respiratory tuberculosis; VK, pneumonia due to a known virus excluding influenza; VP, viral pneumonia.

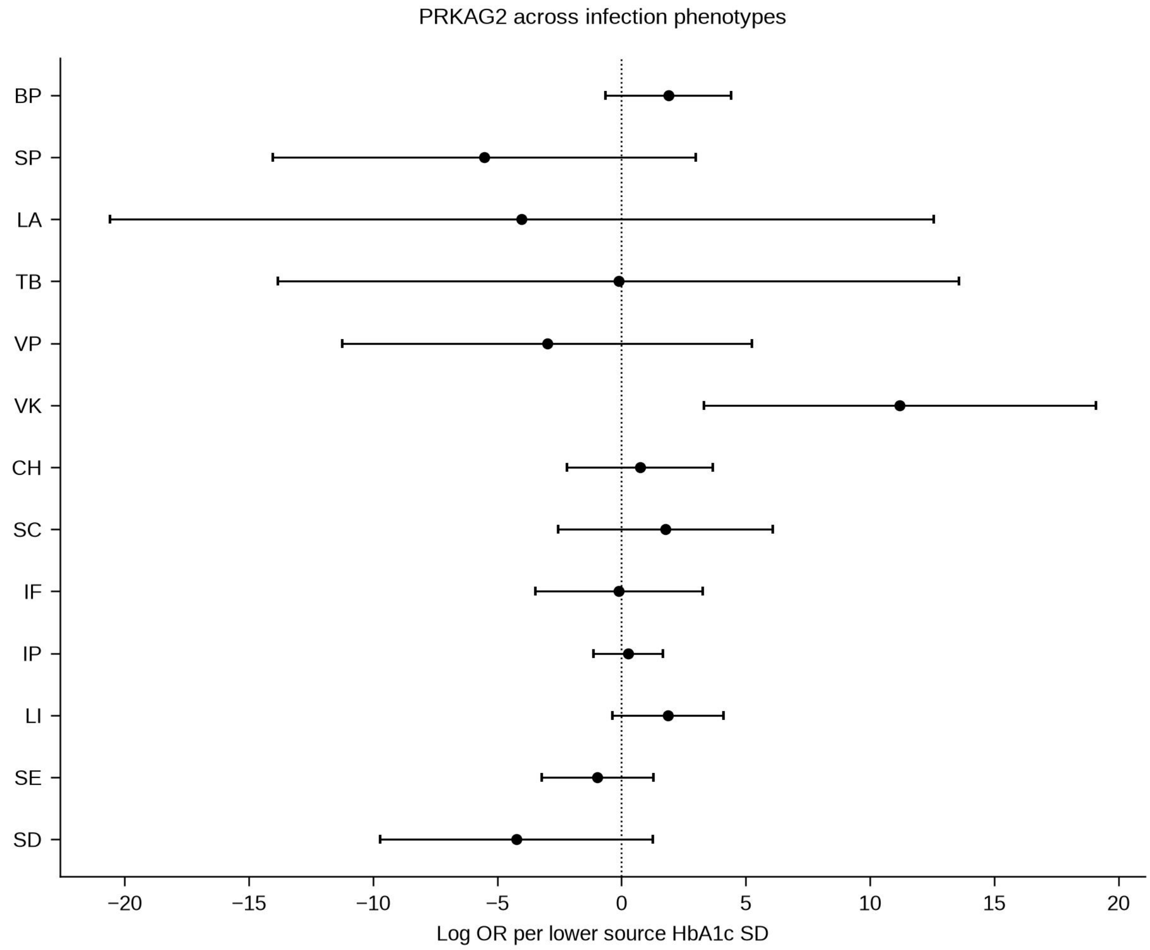

Figure S23. Associations of the *PRKAG2* genetic proxy with infection and sepsis phenotypes. Black circles show single-variant Wald-ratio estimates for the *PRKAG2* proxy rs3793342 across the 13 matched infection and sepsis outcomes. Estimates are log ORs per one source SD lower HbA1c, after alignment to the exposure effect allele. Horizontal bars indicate 95% CIs, and the dotted vertical line marks the null value of zero.

Abbreviations: BP, bacterial pneumonia; CH, COVID-19 hospitalization; CI, confidence interval; COVID-19, coronavirus disease 2019; HbA1c, glycated hemoglobin; IF, influenza of all types; IP, influenza and pneumonia composite; LA, lung abscess; LI, other acute lower respiratory infections; OR, odds ratio; *PRKAG2*, protein kinase AMP-activated non-catalytic subunit gamma 2; SC, severe COVID-19; SD, standard deviation (exposure unit) or sepsis-related 28-day death (phenotype code); SE, sepsis (phenotype code); SP, pneumococcal pneumonia; TB, confirmed respiratory tuberculosis; VK, pneumonia due to a known virus excluding influenza; VP, viral pneumonia.

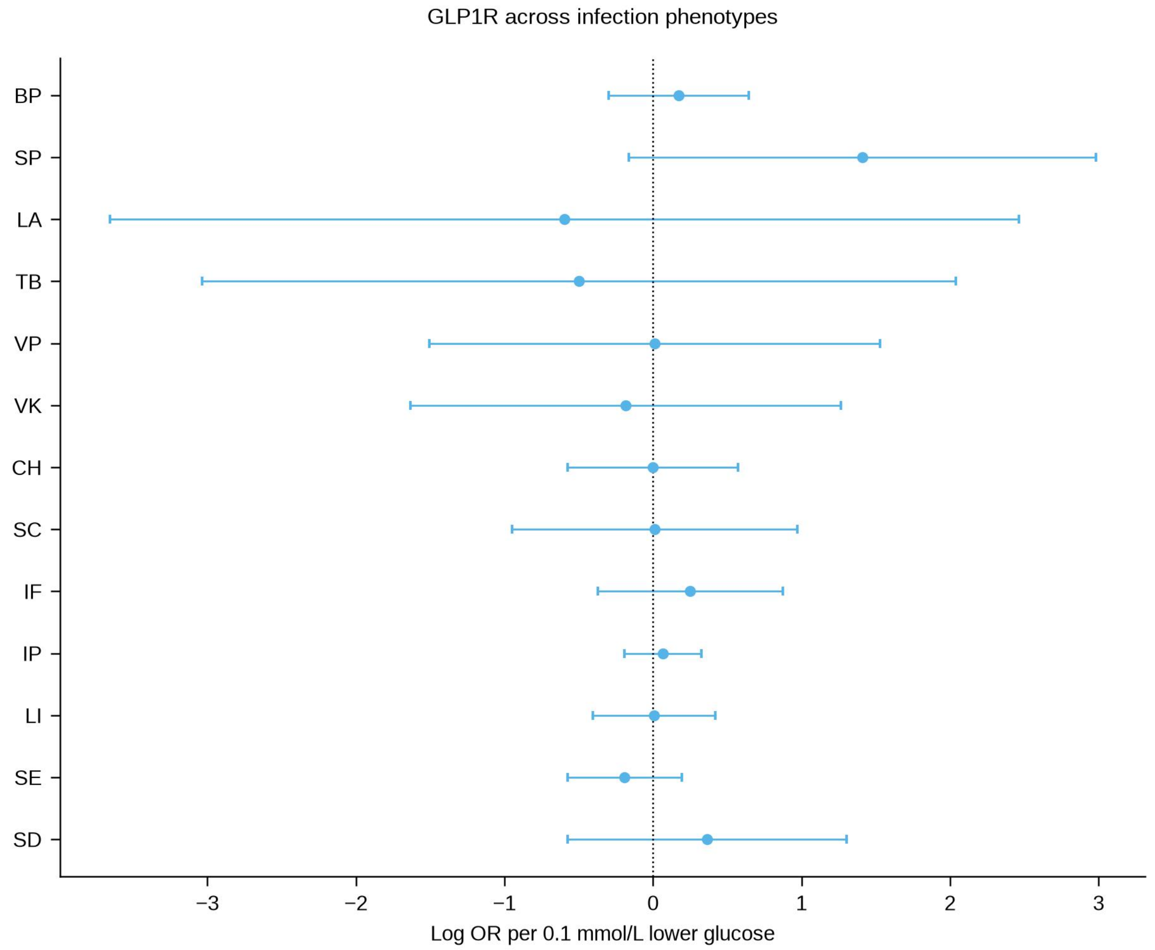

Figure S24. Associations of the *GLP1R* genetic proxy with infection and sepsis phenotypes. Light-blue circles show single-variant Wald-ratio estimates for the *GLP1R* proxy rs1076733 across the 13 matched infection and sepsis outcomes. Estimates are log ORs per 0.1 mmol/L decrease in random glucose, after alignment to the exposure effect allele. Horizontal bars indicate 95% CIs, and the dotted vertical line marks the null value of zero.

Abbreviations: BP, bacterial pneumonia; CH, COVID-19 hospitalization; CI, confidence interval; COVID-19, coronavirus disease 2019; *GLP1R*, glucagon-like peptide 1 receptor; IF, influenza of all types; IP, influenza and pneumonia composite; LA, lung abscess; LI, other acute lower respiratory infections; OR, odds ratio; SC, severe COVID-19; SD, sepsis-related 28-day death (phenotype code); SE, sepsis (phenotype code); SP, pneumococcal pneumonia; TB, confirmed respiratory tuberculosis; VK, pneumonia due to a known virus excluding influenza; VP, viral pneumonia.

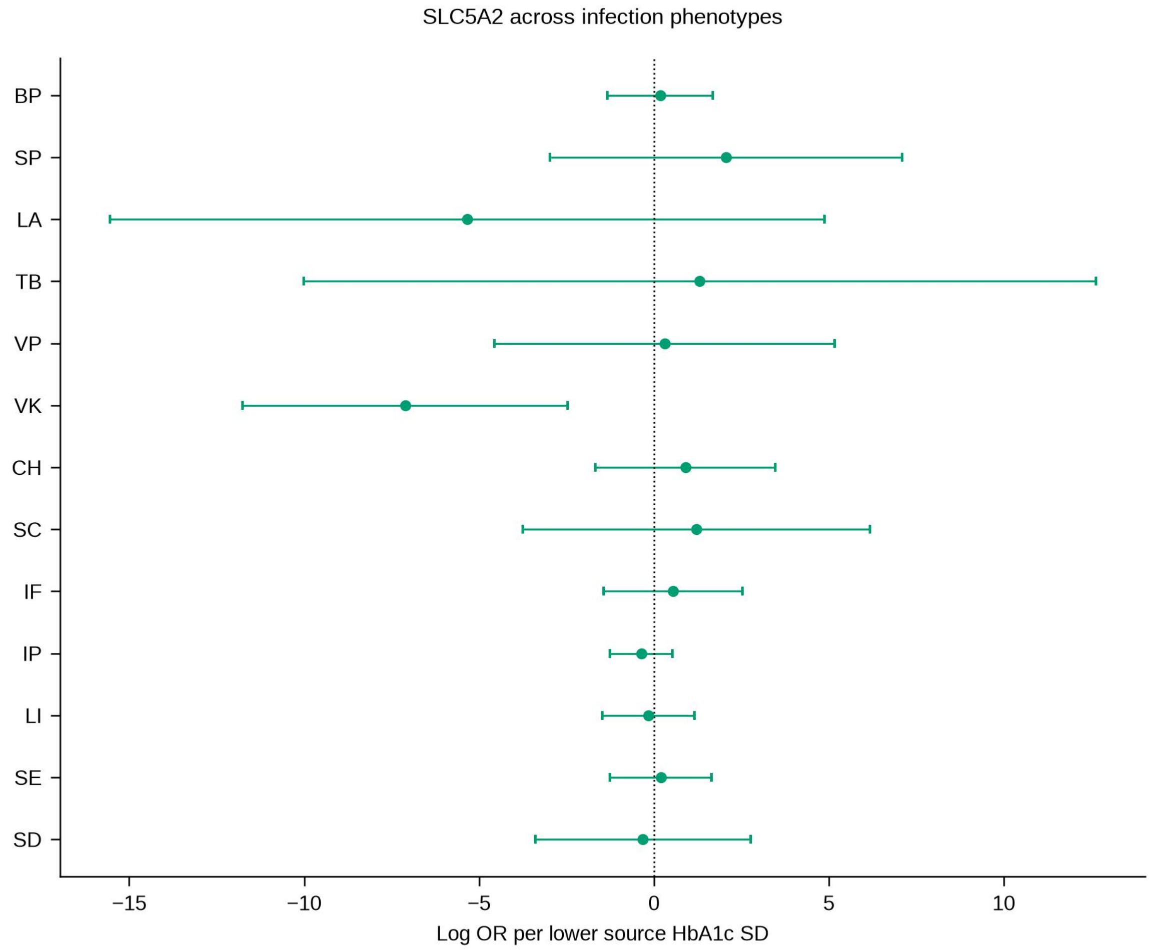

Figure S25. Associations of the *SLC5A2* genetic instrument set with infection and sepsis phenotypes. Green circles show generalized IVW estimates for the *SLC5A2* source instrument set across 13 matched infection and sepsis outcomes. Estimates are log ORs per one source SD lower HbA1c. Six SNPs contribute to each outcome except COVID-19 hospitalization, for which five SNPs are available. Analyses use signed LD aligned to the exposure effect allele and estimated from 503 European participants in the 1000 Genomes Project Phase 3. Horizontal bars indicate 95% CIs calculated using multiplicative random-effects SEs. The dotted vertical line marks the null value of zero.

Abbreviations: BP, bacterial pneumonia; CH, COVID-19 hospitalization; CI, confidence interval; COVID-19, coronavirus disease 2019; HbA1c, glycated hemoglobin; IF, influenza of all types; IP, influenza and pneumonia composite; IVW, inverse-variance weighted; LA, lung abscess; LD, linkage disequilibrium; LI, other acute lower respiratory infections; OR, odds ratio; SC, severe COVID-19; SD, standard deviation (exposure unit) or sepsis-related 28-day death (phenotype code); SE, standard error (statistical quantity) or sepsis (phenotype code); *SLC5A2*, solute carrier family 5 member 2; SNP, single-nucleotide polymorphism; SP, pneumococcal pneumonia; TB, confirmed respiratory tuberculosis; VK, pneumonia due to a known virus excluding influenza; VP, viral pneumonia.

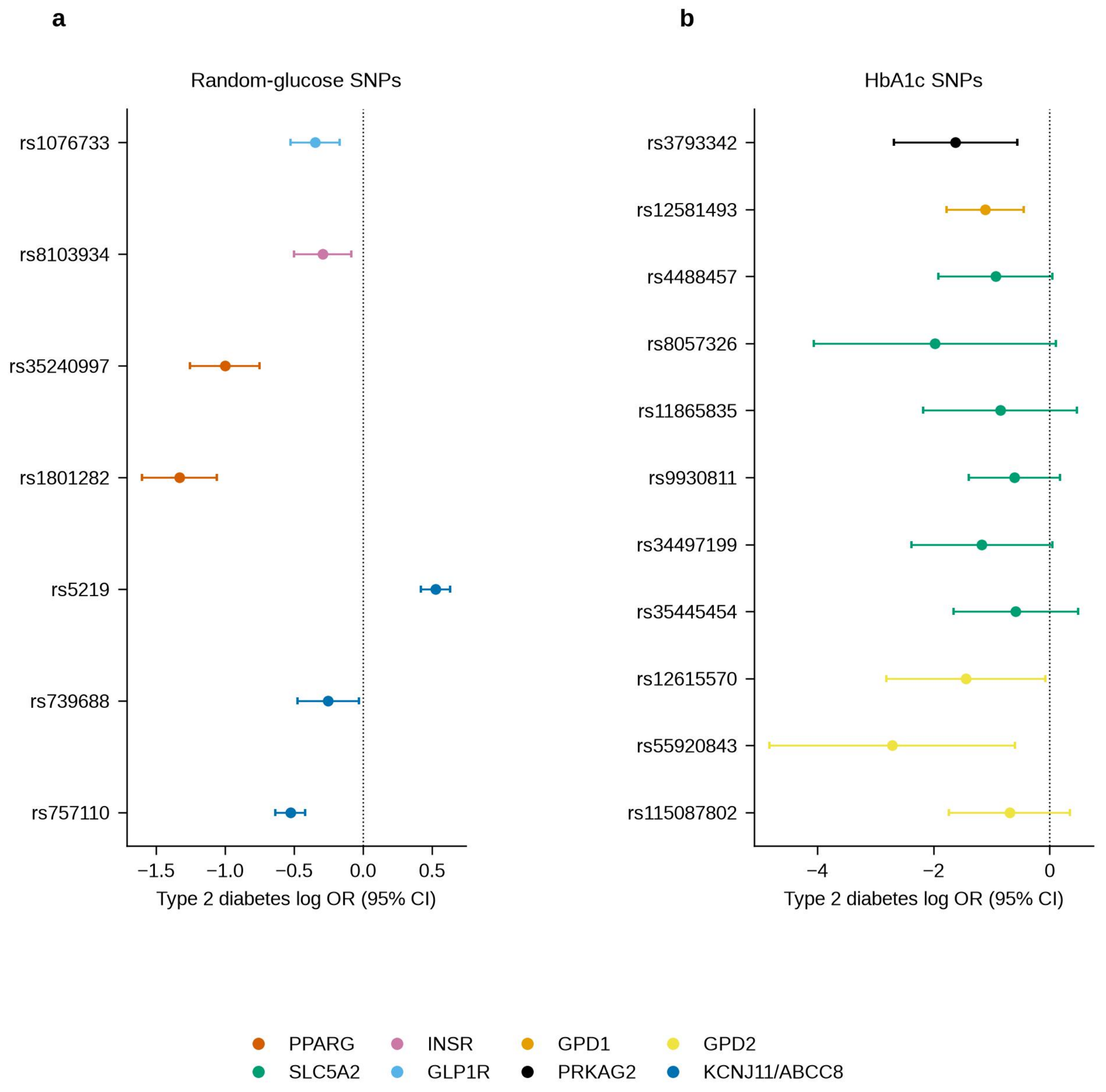

Figure S26. Variant-level type 2 diabetes positive-control associations. **(a)** Seven random-glucose instruments, with estimates expressed per 0.1 mmol/L decrease in random glucose. **(b)** Eleven HbA1c instruments, with estimates expressed per one source SD lower HbA1c. All 18 variants were analyzed against the same type 2 diabetes GWAS accession, ebi-a-GCST90018926, using associations aligned to the exposure effect allele. Circles show individual Wald-ratio log ORs, and horizontal bars show 95% CIs. First-order SEs were calculated as $\frac{SE_Y}{|\beta_X|}$ and multiplied by the reporting-unit factor, where SE_Y is the outcome association SE and β_X is the source exposure association coefficient. Colors identify target genes, as shown in the key. Dotted vertical lines mark zero, and the panels retain separate exposure calibrations and horizontal scales.

Abbreviations: *ABCC8*, ATP-binding cassette subfamily C member 8; CI, confidence interval; *GLP1R*, glucagon-like peptide 1 receptor; *GPD1*, glycerol-3-phosphate dehydrogenase 1; *GPD2*, glycerol-3-phosphate dehydrogenase 2; GWAS, genome-wide association study; HbA1c, glycated hemoglobin; *INSR*, insulin receptor; *KCNJ11*, potassium inwardly rectifying channel subfamily J member 11; OR, odds ratio; *PPARG*, peroxisome proliferator-activated receptor gamma; *PRKAG2*, protein kinase AMP-activated non-catalytic subunit gamma 2; SD, standard deviation; SE, standard error; *SLC5A2*, solute carrier family 5 member 2; SNP, single-nucleotide polymorphism.

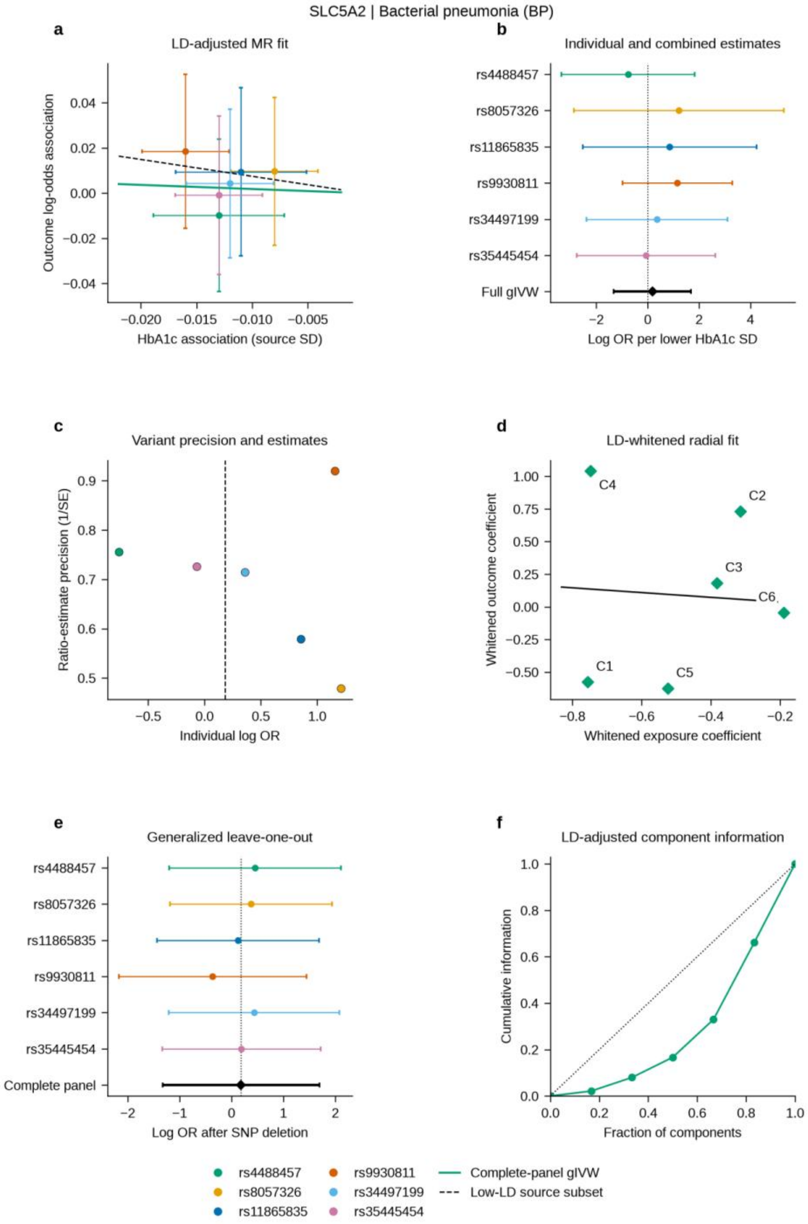

Figure S27. LD-adjusted analyses of *SLC5A2* genetic instruments for bacterial pneumonia. Analyses use six available *SLC5A2* SNPs and outcome accession finn-b-J10_PNEUMOBACT. **(a)** SNP–HbA1c and SNP–outcome associations aligned to the exposure effect allele, with horizontal and vertical 95% CIs. The green solid line shows the complete-panel generalized IVW fit, and the black dashed line shows the corresponding fit for the low-LD source subset rs9930811 and rs35445454. **(b)** Individual Wald-ratio estimates (colored circles) and the complete-panel generalized IVW estimate (black diamond), with 95% CIs; the dotted line marks zero. **(c)** Individual ratio estimates plotted against first-order precision (1/SE); the dashed line marks the complete-panel estimate. **(d)** Cholesky-whitened associations and fitted generalized IVW line; green diamonds labeled C1–C6 denote linear combinations of SNP associations obtained in fixed source order. **(e)** Generalized IVW estimates after omitting each labeled SNP and its covariance row and column, with 95% CIs; the black diamond and dotted line mark the complete-panel estimate. **(f)** Cumulative information from normalized squared whitened exposure coefficients, accumulated from smallest to largest; the effective component count is 3.81, and the diagonal indicates equal information across components. Estimates in (b,c,e) are log ORs per one source SD lower HbA1c. Multi-variant fits use the appropriate signed, effect-allele-aligned LD submatrix from 503 European participants in the 1000 Genomes Project Phase 3. Generalized IVW CIs use multiplicative random-effects SEs with dispersion $max[1, \frac{Q}{n-1}]$, where Q is the LD-adjusted heterogeneity statistic and n is the number of variants in the fitted set. Individual-ratio SEs equal $\frac{SE_Y}{|\beta_X|}$, where SE_Y is the outcome association SE and β_X is the HbA1c association coefficient. Colors in (a–c,e) identify the displayed or omitted SNP.

Abbreviations: BP, bacterial pneumonia; CI, confidence interval; gIVW, generalized inverse-variance weighted; HbA1c, glycated hemoglobin; IVW, inverse-variance weighted; LD, linkage disequilibrium; MR, Mendelian randomization; OR, odds ratio; SD, standard deviation; SE, standard error; *SLC5A2*, solute carrier family 5 member 2; SNP, single-nucleotide polymorphism.

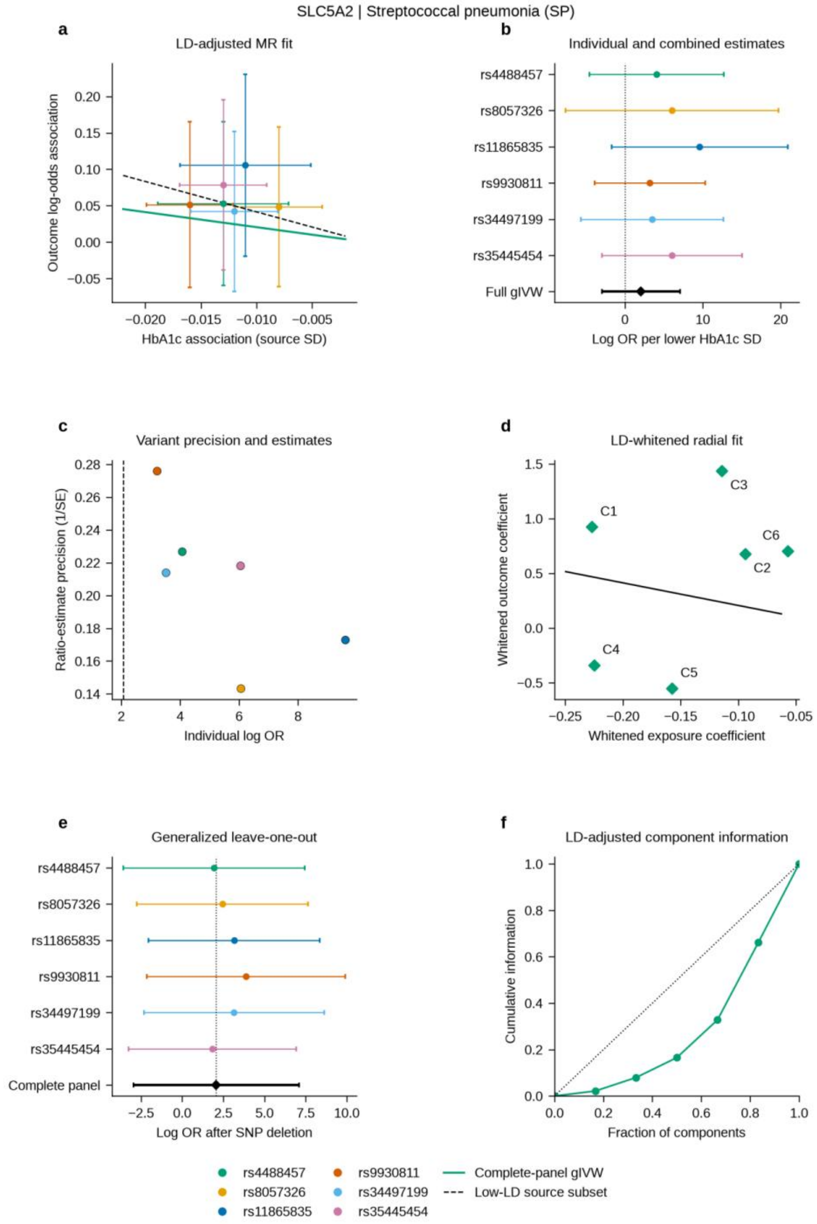

Figure S28. LD-adjusted analyses of *SLC5A2* genetic instruments for pneumococcal pneumonia. Analyses use six available *SLC5A2* SNPs and outcome accession finn-b-J10_PNEUMOPNEUMO. **(a)** SNP–HbA1c and SNP–outcome associations aligned to the exposure effect allele, with horizontal and vertical 95% CIs. The green solid line shows the complete-panel generalized IVW fit, and the black dashed line shows the corresponding fit for the low-LD source subset rs9930811 and rs35445454. **(b)** Individual Wald-ratio estimates (colored circles) and the complete-panel generalized IVW estimate (black diamond), with 95% CIs; the dotted line marks zero. **(c)** Individual ratio estimates plotted against first-order precision (1/SE); the dashed line marks the complete-panel estimate. **(d)** Cholesky-whitened associations and fitted generalized IVW line; green diamonds labeled C1–C6 denote linear combinations of SNP associations obtained in fixed source order. **(e)** Generalized IVW estimates after omitting each labeled SNP and its covariance row and column, with 95% CIs; the black diamond and dotted line mark the complete-panel estimate. **(f)** Cumulative information from normalized squared whitened exposure coefficients, accumulated from smallest to largest; the effective component count is 3.80, and the diagonal indicates equal information across components. Estimates in (b,c,e) are log ORs per one source SD lower HbA1c. Multi-variant fits use the appropriate signed, effect-allele-aligned LD submatrix from 503 European participants in the 1000 Genomes Project Phase 3. Generalized IVW CIs use multiplicative random-effects SEs with dispersion $max[1, \frac{Q}{n-1}]$, where Q is the LD-adjusted heterogeneity statistic and n is the number of variants in the fitted set. Individual-ratio SEs equal $\frac{SE_Y}{|\beta_X|}$, where SE_Y is the outcome association SE and β_X is the HbA1c association coefficient. Colors in (a–c,e) identify the displayed or omitted SNP.

Abbreviations: CI, confidence interval; gIVW, generalized inverse-variance weighted; HbA1c, glycated hemoglobin; IVW, inverse-variance weighted; LD, linkage disequilibrium; MR, Mendelian randomization; OR, odds ratio; SD, standard deviation; SE, standard error; *SLC5A2*, solute carrier family 5 member 2; SNP, single-nucleotide polymorphism; SP, pneumococcal pneumonia.

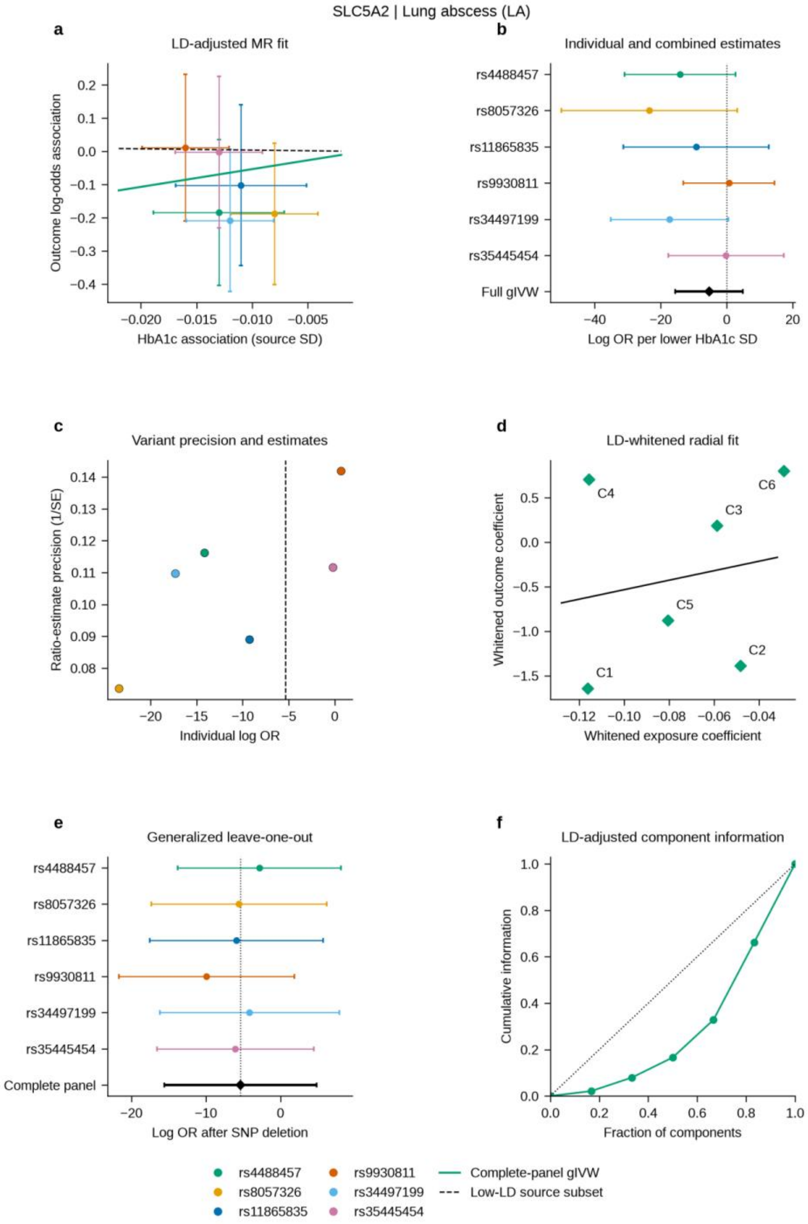

Figure S29. LD-adjusted analyses of *SLC5A2* genetic instruments for lung abscess. Analyses use six available *SLC5A2* SNPs and outcome accession finn-b-J10_ABSCLUNGPNEUMO. **(a)** SNP–HbA1c and SNP–outcome associations aligned to the exposure effect allele, with horizontal and vertical 95% CIs. The green solid line shows the complete-panel generalized IVW fit, and the black dashed line shows the corresponding fit for the low-LD source subset rs9930811 and rs35445454. **(b)** Individual Wald-ratio estimates (colored circles) and the complete-panel generalized IVW estimate (black diamond), with 95% CIs; the dotted line marks zero. **(c)** Individual ratio estimates plotted against first-order precision (1/SE); the dashed line marks the complete-panel estimate. **(d)** Cholesky-whitened associations and fitted generalized IVW line; green diamonds labeled C1–C6 denote linear combinations of SNP associations obtained in fixed source order. **(e)** Generalized IVW estimates after omitting each labeled SNP and its covariance row and column, with 95% CIs; the black diamond and dotted line mark the complete-panel estimate. **(f)** Cumulative information from normalized squared whitened exposure coefficients, accumulated from smallest to largest; the effective component count is 3.80, and the diagonal indicates equal information across components. Estimates in (b,c,e) are log ORs per one source SD lower HbA1c. Multi-variant fits use the appropriate signed, effect-allele-aligned LD submatrix from 503 European participants in the 1000 Genomes Project Phase 3. Generalized IVW CIs use multiplicative random-effects SEs with dispersion $max[1, \frac{Q}{n-1}]$, where Q is the LD-adjusted heterogeneity statistic and n is the number of variants in the fitted set. Individual-ratio SEs equal $\frac{SE_Y}{|\beta_X|}$, where SE_Y is the outcome association SE and β_X is the HbA1c association coefficient. Colors in (a–c,e) identify the displayed or omitted SNP.

Abbreviations: CI, confidence interval; gIVW, generalized inverse-variance weighted; HbA1c, glycated hemoglobin; IVW, inverse-variance weighted; LA, lung abscess; LD, linkage disequilibrium; MR, Mendelian randomization; OR, odds ratio; SD, standard deviation; SE, standard error; *SLC5A2*, solute carrier family 5 member 2; SNP, single-nucleotide polymorphism.

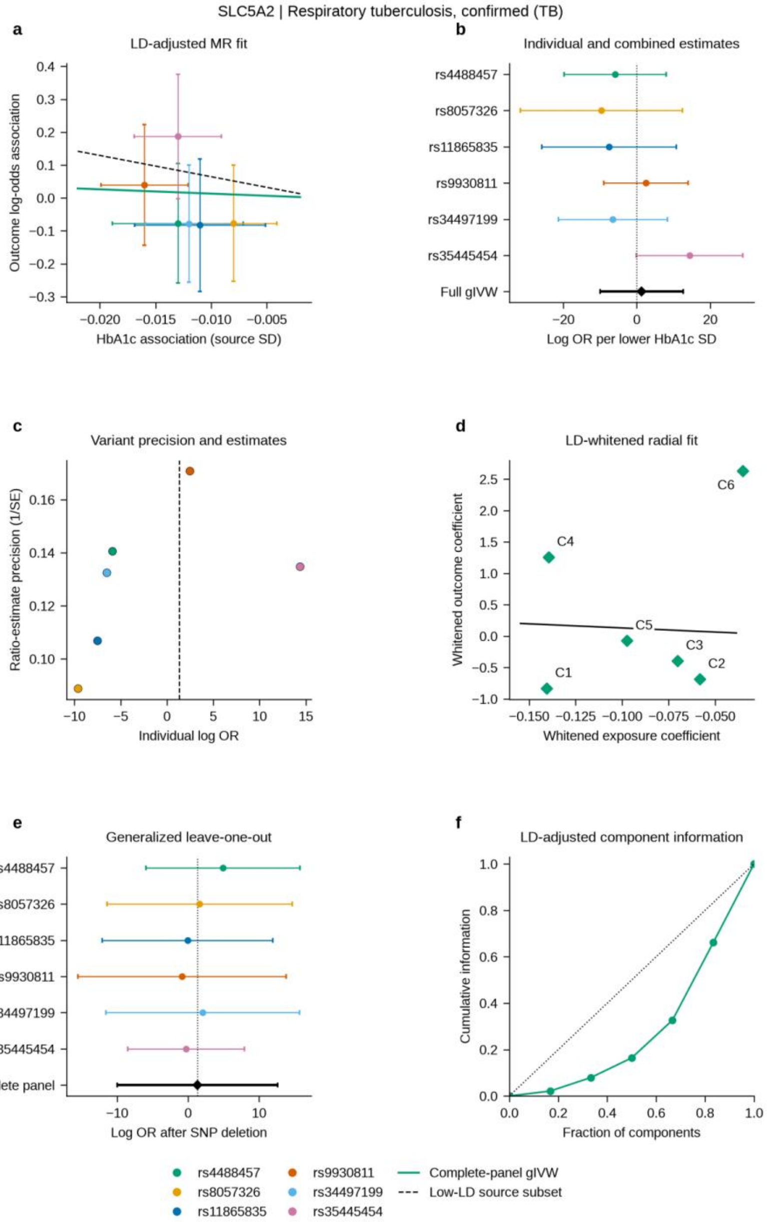

Figure S30. LD-adjusted analyses of *SLC5A2* genetic instruments for confirmed respiratory tuberculosis. Analyses use six available *SLC5A2* SNPs and outcome accession finn-b-AB1_RESP_TUBERCU_CONF. **(a)** SNP–HbA1c and SNP–outcome associations aligned to the exposure effect allele, with horizontal and vertical 95% CIs. The green solid line shows the complete-panel generalized IVW fit, and the black dashed line shows the corresponding fit for the low-LD source subset rs9930811 and rs35445454. **(b)** Individual Wald-ratio estimates (colored circles) and the complete-panel generalized IVW estimate (black diamond), with 95% CIs; the dotted line marks zero. **(c)** Individual ratio estimates plotted against first-order precision (1/SE); the dashed line marks the complete-panel estimate. **(d)** Cholesky-whitened associations and fitted generalized IVW line; green diamonds labeled C1–C6 denote linear combinations of SNP associations obtained in fixed source order. **(e)** Generalized IVW estimates after omitting each labeled SNP and its covariance row and column, with 95% CIs; the black diamond and dotted line mark the complete-panel estimate. **(f)** Cumulative information from normalized squared whitened exposure coefficients, accumulated from smallest to largest; the effective component count is 3.79, and the diagonal indicates equal information across components. Estimates in (b,c,e) are log ORs per one source SD lower HbA1c. Multi-variant fits use the appropriate signed, effect-allele-aligned LD submatrix from 503 European participants in the 1000 Genomes Project Phase 3. Generalized IVW CIs use multiplicative random-effects SEs with dispersion $max[1, \frac{Q}{n-1}]$, where Q is the LD-adjusted heterogeneity statistic and n is the number of variants in the fitted set. Individual-ratio SEs equal $\frac{SE_Y}{|\beta_X|}$, where SE_Y is the outcome association SE and β_X is the HbA1c association coefficient. Colors in (a–c,e) identify the displayed or omitted SNP.

Abbreviations: CI, confidence interval; gIVW, generalized inverse-variance weighted; HbA1c, glycated hemoglobin; IVW, inverse-variance weighted; LD, linkage disequilibrium; MR, Mendelian randomization; OR, odds ratio; SD, standard deviation; SE, standard error; *SLC5A2*, solute carrier family 5 member 2; SNP, single-nucleotide polymorphism; TB, confirmed respiratory tuberculosis.

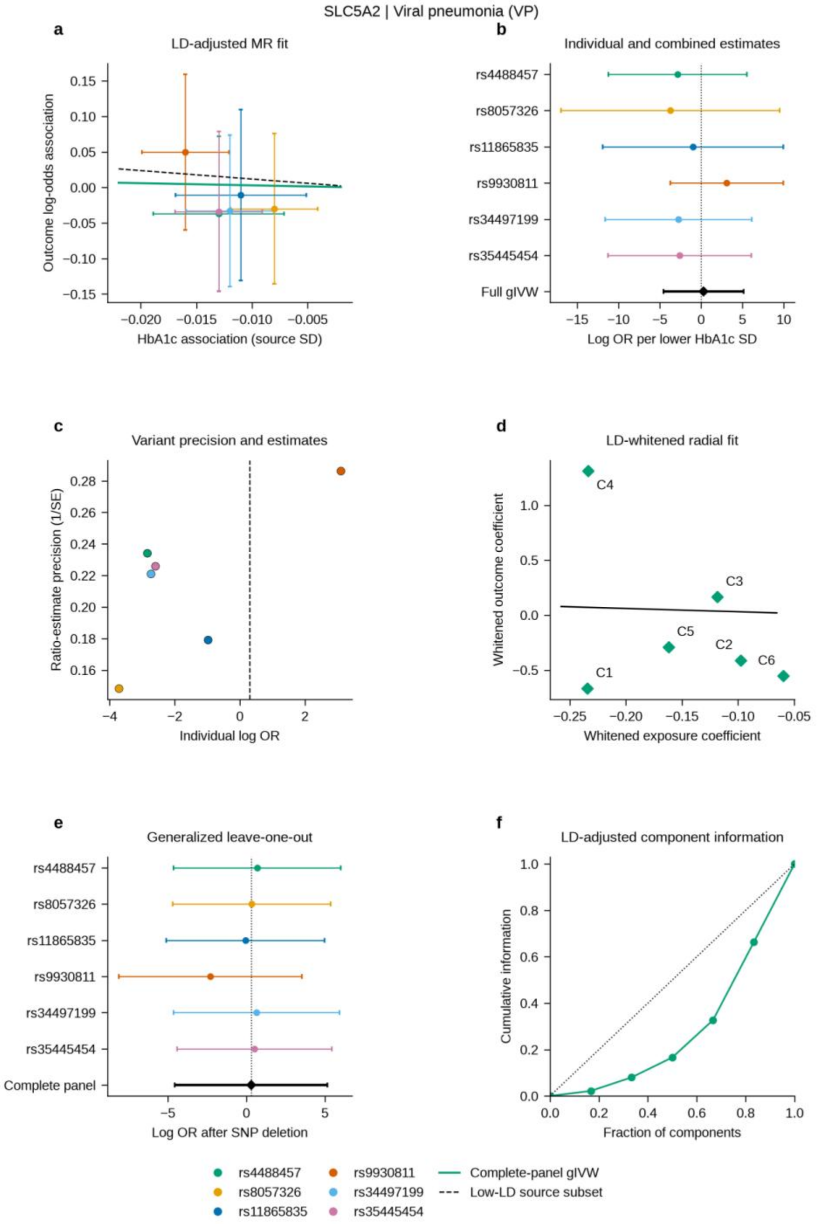

Figure S31. LD-adjusted analyses of *SLC5A2* genetic instruments for viral pneumonia. Analyses use six available *SLC5A2* SNPs and outcome accession finn-b-J10_VIRALPNEUMO. **(a)** SNP–HbA1c and SNP–outcome associations aligned to the exposure effect allele, with horizontal and vertical 95% CIs. The green solid line shows the complete-panel generalized IVW fit, and the black dashed line shows the corresponding fit for the low-LD source subset rs9930811 and rs35445454. **(b)** Individual Wald-ratio estimates (colored circles) and the complete-panel generalized IVW estimate (black diamond), with 95% CIs; the dotted line marks zero. **(c)** Individual ratio estimates plotted against first-order precision (1/SE); the dashed line marks the complete-panel estimate. **(d)** Cholesky-whitened associations and fitted generalized IVW line; green diamonds labeled C1–C6 denote linear combinations of SNP associations obtained in fixed source order. **(e)** Generalized IVW estimates after omitting each labeled SNP and its covariance row and column, with 95% CIs; the black diamond and dotted line mark the complete-panel estimate. **(f)** Cumulative information from normalized squared whitened exposure coefficients, accumulated from smallest to largest; the effective component count is 3.79, and the diagonal indicates equal information across components. Estimates in (b,c,e) are log ORs per one source SD lower HbA1c. Multi-variant fits use the appropriate signed, effect-allele-aligned LD submatrix from 503 European participants in the 1000 Genomes Project Phase 3. Generalized IVW CIs use multiplicative random-effects SEs with dispersion $max[1, \frac{Q}{n-1}]$, where Q is the LD-adjusted heterogeneity statistic and n is the number of variants in the fitted set. Individual-ratio SEs equal $\frac{SE_Y}{|\beta_X|}$, where SE_Y is the outcome association SE and β_X is the HbA1c association coefficient. Colors in (a–c,e) identify the displayed or omitted SNP.

Abbreviations: CI, confidence interval; gIVW, generalized inverse-variance weighted; HbA1c, glycated hemoglobin; IVW, inverse-variance weighted; LD, linkage disequilibrium; MR, Mendelian randomization; OR, odds ratio; SD, standard deviation; SE, standard error; *SLC5A2*, solute carrier family 5 member 2; SNP, single-nucleotide polymorphism; VP, viral pneumonia.

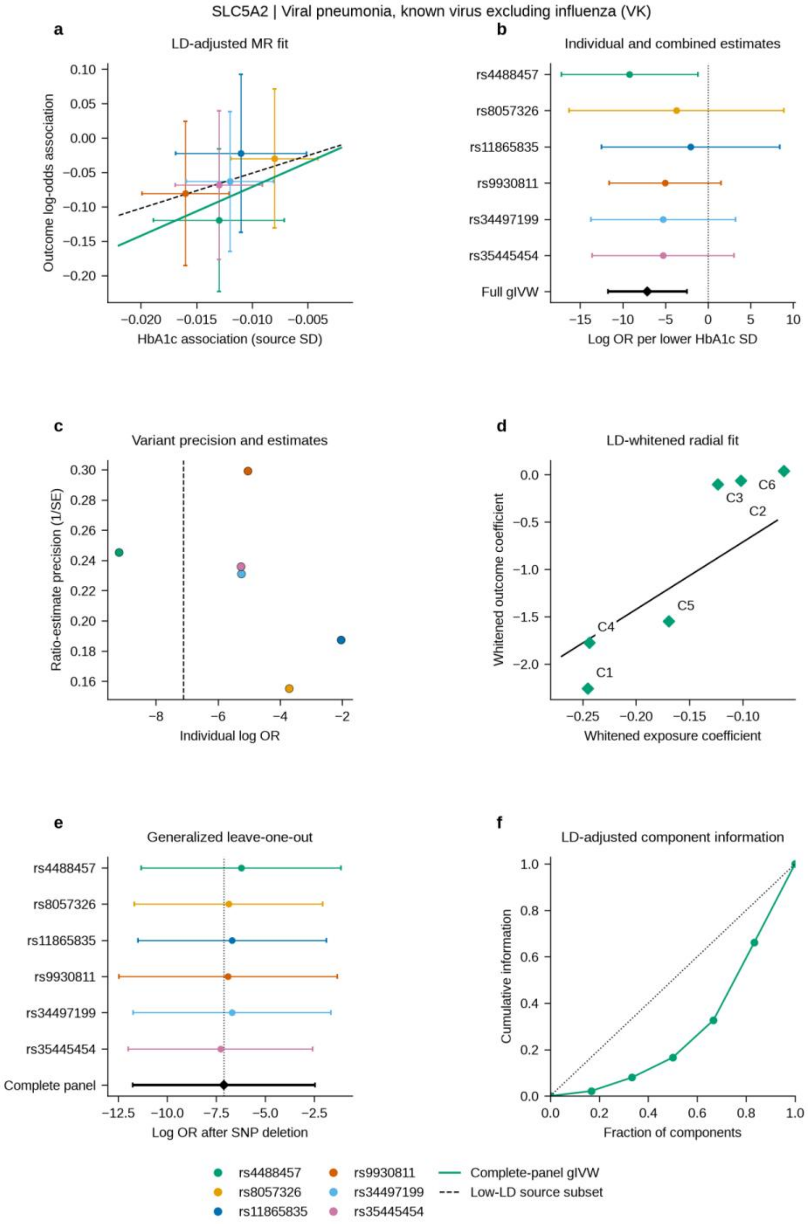

Figure S32. LD-adjusted analyses of *SLC5A2* genetic instruments for pneumonia due to a known virus excluding influenza. Analyses use six available *SLC5A2* SNPs and outcome accession finn-b-VIRALPNEUMOKNOWN. **(a)** SNP–HbA1c and SNP–outcome associations aligned to the exposure effect allele, with horizontal and vertical 95% CIs. The green solid line shows the complete-panel generalized IVW fit, and the black dashed line shows the corresponding fit for the low-LD source subset rs9930811 and rs35445454. **(b)** Individual Wald-ratio estimates (colored circles) and the complete-panel generalized IVW estimate (black diamond), with 95% CIs; the dotted line marks zero. **(c)** Individual ratio estimates plotted against first-order precision (1/SE); the dashed line marks the complete-panel estimate. **(d)** Cholesky-whitened associations and fitted generalized IVW line; green diamonds labeled C1–C6 denote linear combinations of SNP associations obtained in fixed source order. **(e)** Generalized IVW estimates after omitting each labeled SNP and its covariance row and column, with 95% CIs; the black diamond and dotted line mark the complete-panel estimate. **(f)** Cumulative information from normalized squared whitened exposure coefficients, accumulated from smallest to largest; the effective component count is 3.79, and the diagonal indicates equal information across components. Estimates in (b,c,e) are log ORs per one source SD lower HbA1c. Multi-variant fits use the appropriate signed, effect-allele-aligned LD submatrix from 503 European participants in the 1000 Genomes Project Phase 3. Generalized IVW CIs use multiplicative random-effects SEs with dispersion $max[1, \frac{Q}{n-1}]$, where Q is the LD-adjusted heterogeneity statistic and n is the number of variants in the fitted set. Individual-ratio SEs equal $\frac{SE_Y}{|\beta_X|}$, where SE_Y is the outcome association SE and β_X is the HbA1c association coefficient. Colors in (a–c,e) identify the displayed or omitted SNP.

Abbreviations: CI, confidence interval; gIVW, generalized inverse-variance weighted; HbA1c, glycated hemoglobin; IVW, inverse-variance weighted; LD, linkage disequilibrium; MR, Mendelian randomization; OR, odds ratio; SD, standard deviation; SE, standard error; *SLC5A2*, solute carrier family 5 member 2; SNP, single-nucleotide polymorphism; VK, pneumonia due to a known virus excluding influenza.

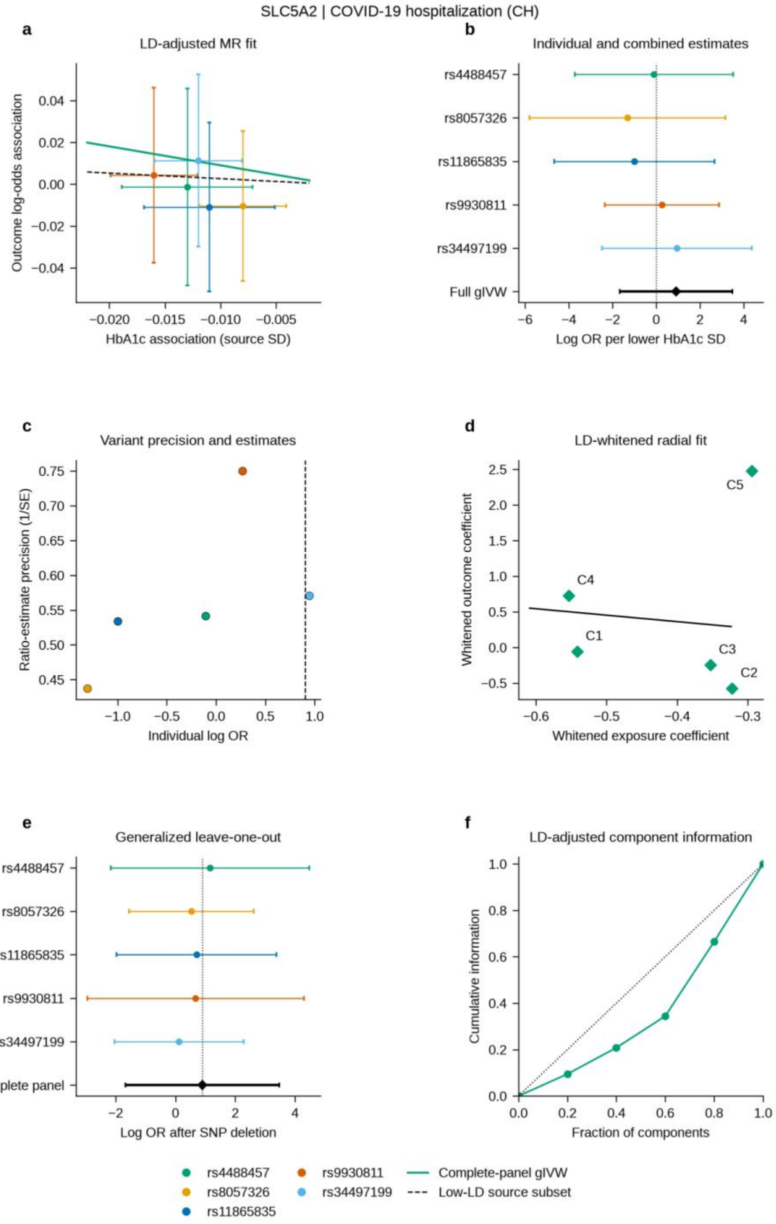

Figure S33. LD-adjusted analyses of *SLC5A2* genetic instruments for COVID-19 hospitalization. Analyses use five available *SLC5A2* SNPs and outcome accession ebi-a-GCST011081. **(a)** SNP–HbA1c and SNP–outcome associations aligned to the exposure effect allele, with horizontal and vertical 95% CIs. The green solid line shows the complete-panel generalized IVW fit, and the black dashed line shows the Wald-ratio fit for the available low-LD source subset, rs9930811; rs35445454 is unavailable for this outcome. **(b)** Individual Wald-ratio estimates (colored circles) and the complete-panel generalized IVW estimate (black diamond), with 95% CIs; the dotted line marks zero. **(c)** Individual ratio estimates plotted against first-order precision (1/SE); the dashed line marks the complete-panel estimate. **(d)** Cholesky-whitened associations and fitted generalized IVW line; green diamonds labeled C1–C5 denote linear combinations of SNP associations obtained in fixed source order. **(e)** Generalized IVW estimates after omitting each labeled SNP and its covariance row and column, with 95% CIs; the black diamond and dotted line mark the complete-panel estimate. **(f)** Cumulative information from normalized squared whitened exposure coefficients, accumulated from smallest to largest; the effective component count is 3.91, and the diagonal indicates equal information across components. Estimates in (b,c,e) are log ORs per one source SD lower HbA1c. Multi-variant fits use the appropriate signed, effect-allele-aligned LD submatrix from 503 European participants in the 1000 Genomes Project Phase 3. Generalized IVW CIs use multiplicative random-effects SEs with dispersion $max[1, \frac{Q}{n-1}]$, where Q is the LD-adjusted heterogeneity statistic and n is the number of variants in the fitted set. Individual-ratio SEs equal $\frac{SE_Y}{|\beta_X|}$, where SE_Y is the outcome association SE and β_X is the HbA1c association coefficient. Colors in (a–c,e) identify the displayed or omitted SNP.

Abbreviations: CH, COVID-19 hospitalization; CI, confidence interval; COVID-19, coronavirus disease 2019; gIVW, generalized inverse-variance weighted; HbA1c, glycated hemoglobin; IVW, inverse-variance weighted; LD, linkage disequilibrium; MR, Mendelian randomization; OR, odds ratio; SD, standard deviation; SE, standard error; *SLC5A2*, solute carrier family 5 member 2; SNP, single-nucleotide polymorphism.

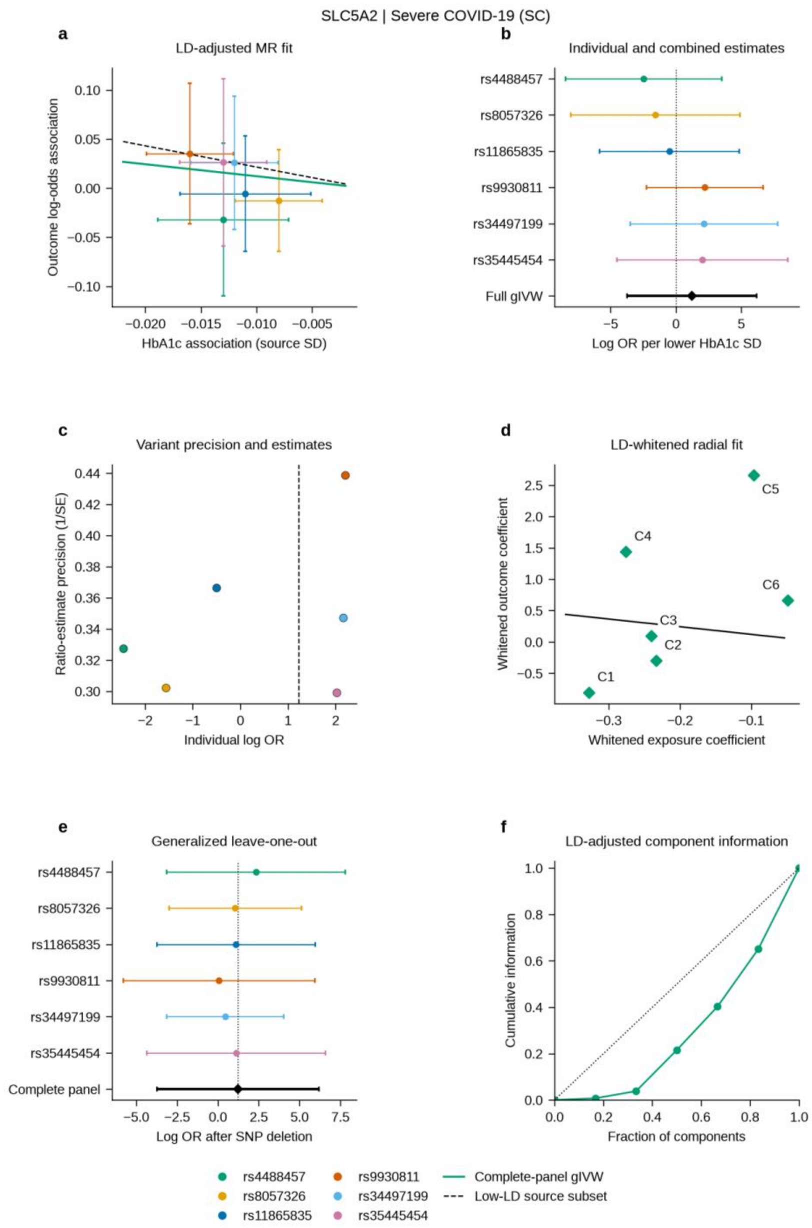

Figure S34. LD-adjusted analyses of *SLC5A2* genetic instruments for severe COVID-19. Analyses use six available *SLC5A2* SNPs and outcome accession ebi-a-GCST011075. **(a)** SNP–HbA1c and SNP–outcome associations aligned to the exposure effect allele, with horizontal and vertical 95% CIs. The green solid line shows the complete-panel generalized IVW fit, and the black dashed line shows the corresponding fit for the low-LD source subset rs9930811 and rs35445454. **(b)** Individual Wald-ratio estimates (colored circles) and the complete-panel generalized IVW estimate (black diamond), with 95% CIs; the dotted line marks zero. **(c)** Individual ratio estimates plotted against first-order precision (1/SE); the dashed line marks the complete-panel estimate. **(d)** Cholesky-whitened associations and fitted generalized IVW line; green diamonds labeled C1–C6 denote linear combinations of SNP associations obtained in fixed source order. **(e)** Generalized IVW estimates after omitting each labeled SNP and its covariance row and column, with 95% CIs; the black diamond and dotted line mark the complete-panel estimate. **(f)** Cumulative information from normalized squared whitened exposure coefficients, accumulated from smallest to largest; the effective component count is 3.98, and the diagonal indicates equal information across components. Estimates in (b,c,e) are log ORs per one source SD lower HbA1c. Multi-variant fits use the appropriate signed, effect-allele-aligned LD submatrix from 503 European participants in the 1000 Genomes Project Phase 3. Generalized IVW CIs use multiplicative random-effects SEs with dispersion $max[1, \frac{Q}{n-1}]$, where Q is the LD-adjusted heterogeneity statistic and n is the number of variants in the fitted set. Individual-ratio SEs equal $\frac{SE_Y}{|\beta_X|}$, where SE_Y is the outcome association SE and β_X is the HbA1c association coefficient. Colors in (a–c,e) identify the displayed or omitted SNP.

Abbreviations: CI, confidence interval; COVID-19, coronavirus disease 2019; gIVW, generalized inverse-variance weighted; HbA1c, glycated hemoglobin; IVW, inverse-variance weighted; LD, linkage disequilibrium; MR, Mendelian randomization; OR, odds ratio; SC, severe COVID-19; SD, standard deviation; SE, standard error; *SLC5A2*, solute carrier family 5 member 2; SNP, single-nucleotide polymorphism.

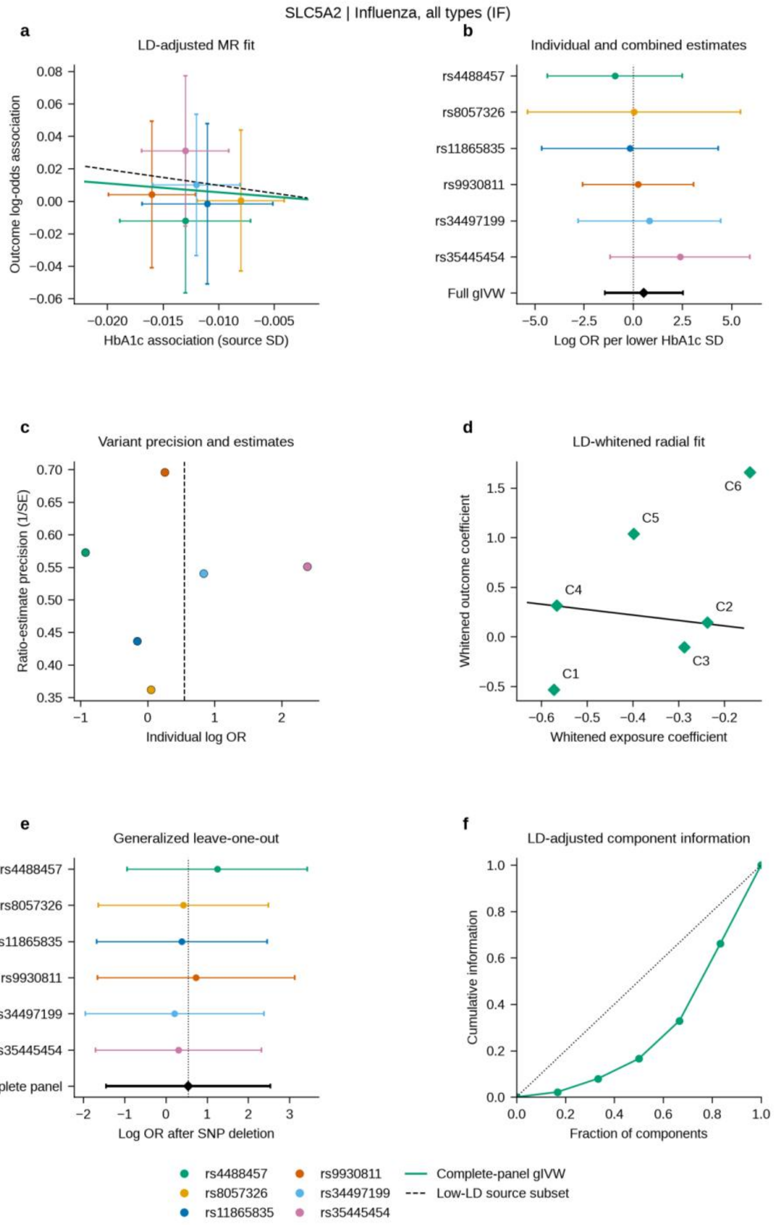

Figure S35. LD-adjusted analyses of *SLC5A2* genetic instruments for influenza of all types. Analyses use six available *SLC5A2* SNPs and outcome accession finn-b-J10_INFLUENZA. **(a)** SNP–HbA1c and SNP–outcome associations aligned to the exposure effect allele, with horizontal and vertical 95% CIs. The green solid line shows the complete-panel generalized IVW fit, and the black dashed line shows the corresponding fit for the low-LD source subset rs9930811 and rs35445454. **(b)** Individual Wald-ratio estimates (colored circles) and the complete-panel generalized IVW estimate (black diamond), with 95% CIs; the dotted line marks zero. **(c)** Individual ratio estimates plotted against first-order precision (1/SE); the dashed line marks the complete-panel estimate. **(d)** Cholesky-whitened associations and fitted generalized IVW line; green diamonds labeled C1–C6 denote linear combinations of SNP associations obtained in fixed source order. **(e)** Generalized IVW estimates after omitting each labeled SNP and its covariance row and column, with 95% CIs; the black diamond and dotted line mark the complete-panel estimate. **(f)** Cumulative information from normalized squared whitened exposure coefficients, accumulated from smallest to largest; the effective component count is 3.80, and the diagonal indicates equal information across components. Estimates in (b,c,e) are log ORs per one source SD lower HbA1c. Multi-variant fits use the appropriate signed, effect-allele-aligned LD submatrix from 503 European participants in the 1000 Genomes Project Phase 3. Generalized IVW CIs use multiplicative random-effects SEs with dispersion $max[1, \frac{Q}{n-1}]$, where Q is the LD-adjusted heterogeneity statistic and n is the number of variants in the fitted set. Individual-ratio SEs equal $\frac{SE_Y}{|\beta_X|}$, where SE_Y is the outcome association SE and β_X is the HbA1c association coefficient. Colors in (a–c,e) identify the displayed or omitted SNP.

Abbreviations: CI, confidence interval; gIVW, generalized inverse-variance weighted; HbA1c, glycated hemoglobin; IF, influenza of all types; IVW, inverse-variance weighted; LD, linkage disequilibrium; MR, Mendelian randomization; OR, odds ratio; SD, standard deviation; SE, standard error; *SLC5A2*, solute carrier family 5 member 2; SNP, single-nucleotide polymorphism.

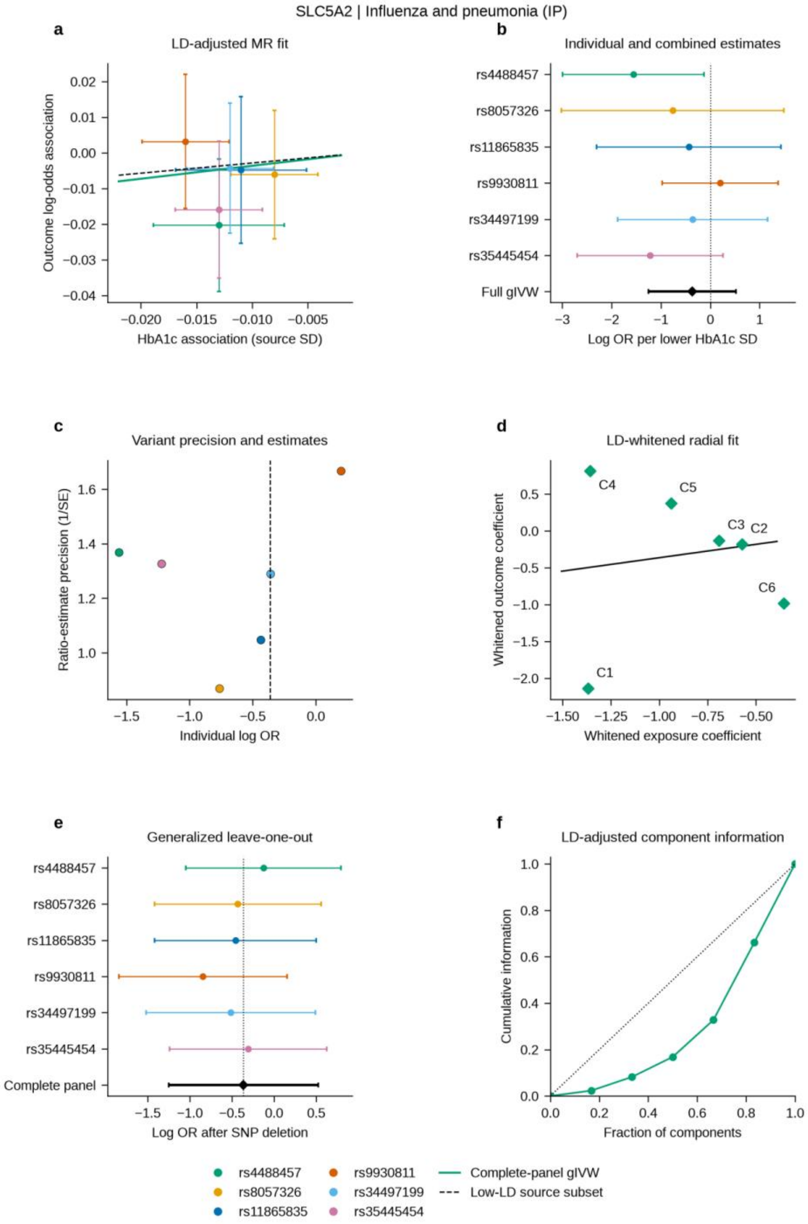

Figure S36. LD-adjusted analyses of *SLC5A2* genetic instruments for influenza and pneumonia composite. Analyses use six available *SLC5A2* SNPs and outcome accession finn-b-J10_INFLUPNEU. **(a)** SNP–HbA1c and SNP–outcome associations aligned to the exposure effect allele, with horizontal and vertical 95% CIs. The green solid line shows the complete-panel generalized IVW fit, and the black dashed line shows the corresponding fit for the low-LD source subset rs9930811 and rs35445454. **(b)** Individual Wald-ratio estimates (colored circles) and the complete-panel generalized IVW estimate (black diamond), with 95% CIs; the dotted line marks zero. **(c)** Individual ratio estimates plotted against first-order precision (1/SE); the dashed line marks the complete-panel estimate. **(d)** Cholesky-whitened associations and fitted generalized IVW line; green diamonds labeled C1–C6 denote linear combinations of SNP associations obtained in fixed source order. **(e)** Generalized IVW estimates after omitting each labeled SNP and its covariance row and column, with 95% CIs; the black diamond and dotted line mark the complete-panel estimate. **(f)** Cumulative information from normalized squared whitened exposure coefficients, accumulated from smallest to largest; the effective component count is 3.81, and the diagonal indicates equal information across components. Estimates in (b,c,e) are log ORs per one source SD lower HbA1c. Multi-variant fits use the appropriate signed, effect-allele-aligned LD submatrix from 503 European participants in the 1000 Genomes Project Phase 3. Generalized IVW CIs use multiplicative random-effects SEs with dispersion $max[1, \frac{Q}{n-1}]$, where Q is the LD-adjusted heterogeneity statistic and n is the number of variants in the fitted set. Individual-ratio SEs equal $\frac{SE_Y}{|\beta_X|}$, where SE_Y is the outcome association SE and β_X is the HbA1c association coefficient. Colors in (a–c,e) identify the displayed or omitted SNP.

Abbreviations: CI, confidence interval; gIVW, generalized inverse-variance weighted; HbA1c, glycated hemoglobin; IP, influenza and pneumonia composite; IVW, inverse-variance weighted; LD, linkage disequilibrium; MR, Mendelian randomization; OR, odds ratio; SD, standard deviation; SE, standard error; *SLC5A2*, solute carrier family 5 member 2; SNP, single-nucleotide polymorphism.

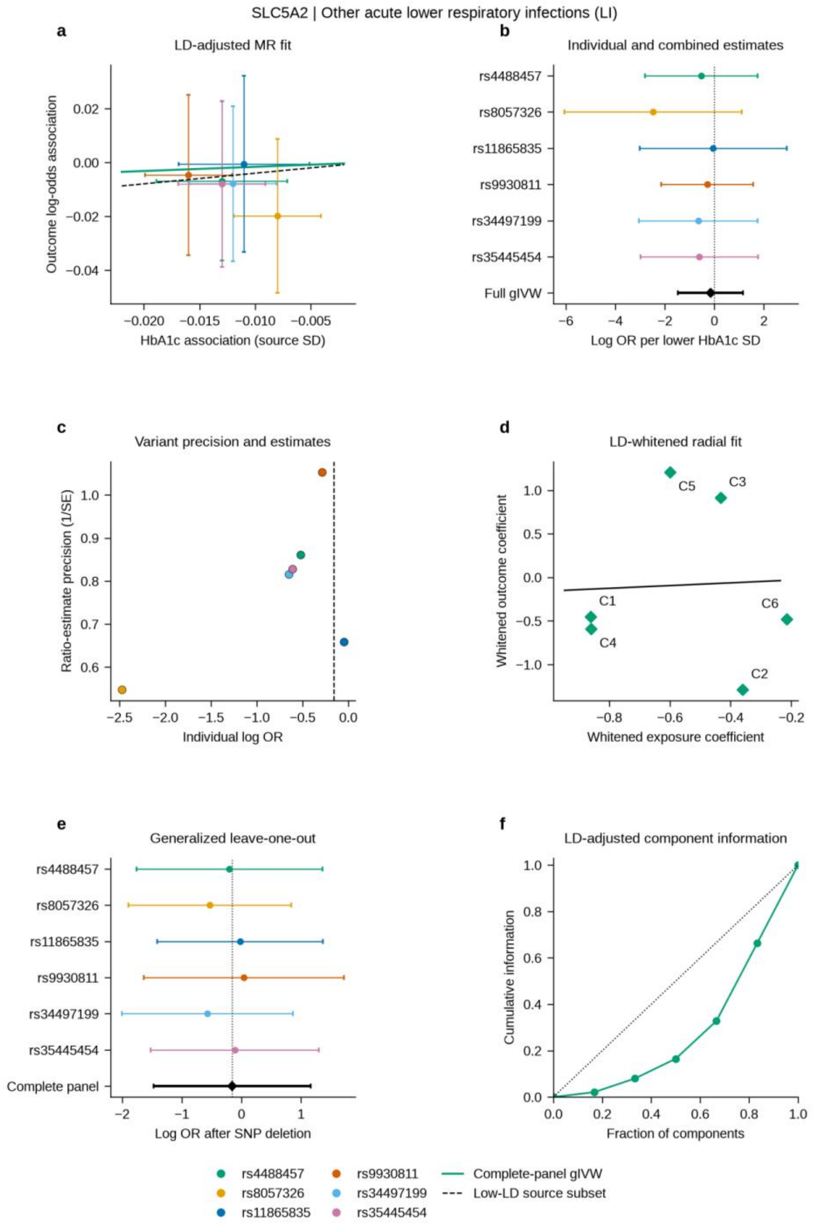

Figure S37. LD-adjusted analyses of *SLC5A2* genetic instruments for other acute lower respiratory infections. Analyses use six available *SLC5A2* SNPs and outcome accession finn-b-J10_LOWERINF. **(a)** SNP–HbA1c and SNP–outcome associations aligned to the exposure effect allele, with horizontal and vertical 95% CIs. The green solid line shows the complete-panel generalized IVW fit, and the black dashed line shows the corresponding fit for the low-LD source subset rs9930811 and rs35445454. **(b)** Individual Wald-ratio estimates (colored circles) and the complete-panel generalized IVW estimate (black diamond), with 95% CIs; the dotted line marks zero. **(c)** Individual ratio estimates plotted against first-order precision (1/SE); the dashed line marks the complete-panel estimate. **(d)** Cholesky-whitened associations and fitted generalized IVW line; green diamonds labeled C1–C6 denote linear combinations of SNP associations obtained in fixed source order. **(e)** Generalized IVW estimates after omitting each labeled SNP and its covariance row and column, with 95% CIs; the black diamond and dotted line mark the complete-panel estimate. **(f)** Cumulative information from normalized squared whitened exposure coefficients, accumulated from smallest to largest; the effective component count is 3.80, and the diagonal indicates equal information across components. Estimates in (b,c,e) are log ORs per one source SD lower HbA1c. Multi-variant fits use the appropriate signed, effect-allele-aligned LD submatrix from 503 European participants in the 1000 Genomes Project Phase 3. Generalized IVW CIs use multiplicative random-effects SEs with dispersion $max[1, \frac{Q}{n-1}]$, where Q is the LD-adjusted heterogeneity statistic and n is the number of variants in the fitted set. Individual-ratio SEs equal $\frac{SE_Y}{|\beta_X|}$, where SE_Y is the outcome association SE and β_X is the HbA1c association coefficient. Colors in (a–c,e) identify the displayed or omitted SNP.

Abbreviations: CI, confidence interval; gIVW, generalized inverse-variance weighted; HbA1c, glycated hemoglobin; IVW, inverse-variance weighted; LD, linkage disequilibrium; LI, other acute lower respiratory infections; MR, Mendelian randomization; OR, odds ratio; SD, standard deviation; SE, standard error; *SLC5A2*, solute carrier family 5 member 2; SNP, single-nucleotide polymorphism.

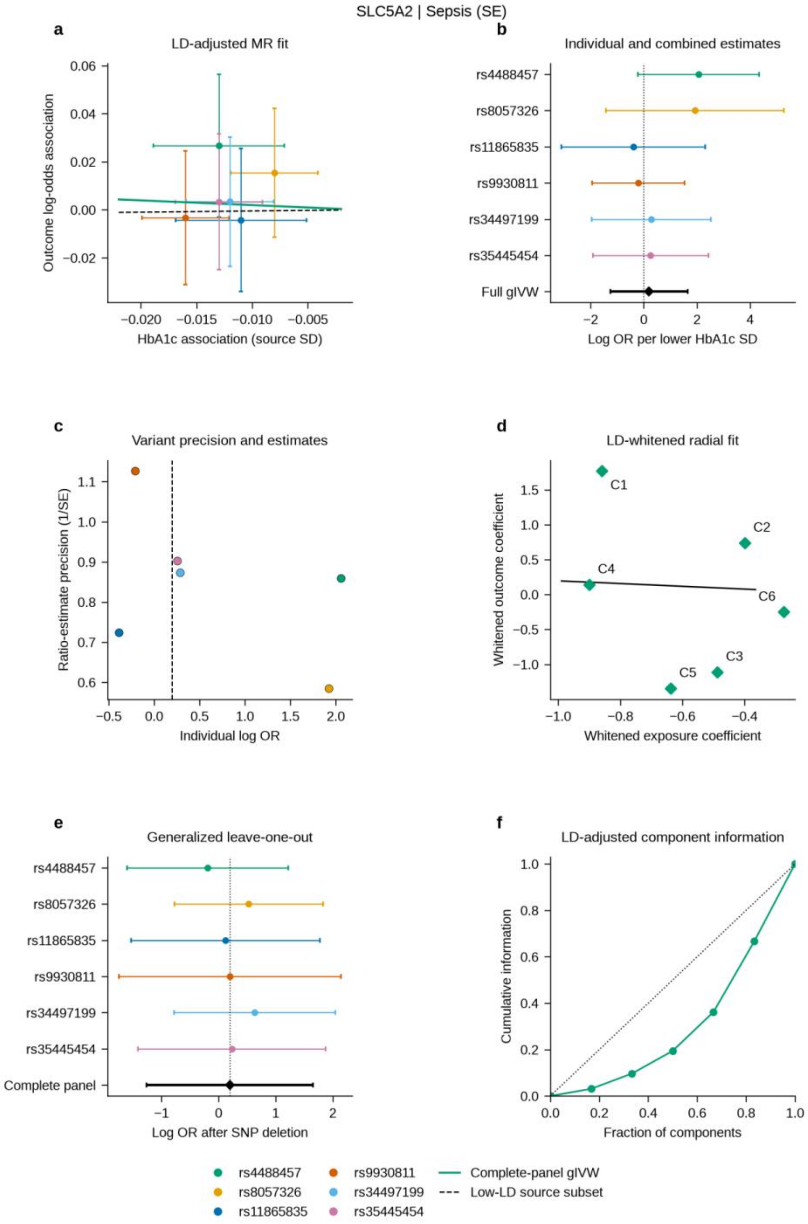

Figure S38. LD-adjusted analyses of *SLC5A2* genetic instruments for sepsis. Analyses use six available *SLC5A2* SNPs and outcome accession ieu-b-4980. **(a)** SNP–HbA1c and SNP–outcome associations aligned to the exposure effect allele, with horizontal and vertical 95% CIs. The green solid line shows the complete-panel generalized IVW fit, and the black dashed line shows the corresponding fit for the low-LD source subset rs9930811 and rs35445454. **(b)** Individual Wald-ratio estimates (colored circles) and the complete-panel generalized IVW estimate (black diamond), with 95% CIs; the dotted line marks zero. **(c)** Individual ratio estimates plotted against first-order precision (1/SE); the dashed line marks the complete-panel estimate. **(d)** Cholesky-whitened associations and fitted generalized IVW line; green diamonds labeled C1–C6 denote linear combinations of SNP associations obtained in fixed source order. **(e)** Generalized IVW estimates after omitting each labeled SNP and its covariance row and column, with 95% CIs; the black diamond and dotted line mark the complete-panel estimate. **(f)** Cumulative information from normalized squared whitened exposure coefficients, accumulated from smallest to largest; the effective component count is 4.05, and the diagonal indicates equal information across components. Estimates in (b,c,e) are log ORs per one source SD lower HbA1c. Multi-variant fits use the appropriate signed, effect-allele-aligned LD submatrix from 503 European participants in the 1000 Genomes Project Phase 3. Generalized IVW CIs use multiplicative random-effects SEs with dispersion $max[1, \frac{Q}{n-1}]$, where Q is the LD-adjusted heterogeneity statistic and n is the number of variants in the fitted set. Individual-ratio SEs equal $\frac{SE_Y}{|\beta_X|}$, where SE_Y is the outcome association SE and β_X is the HbA1c association coefficient. Colors in (a–c,e) identify the displayed or omitted SNP.

Abbreviations: CI, confidence interval; gIVW, generalized inverse-variance weighted; HbA1c, glycated hemoglobin; IVW, inverse-variance weighted; LD, linkage disequilibrium; MR, Mendelian randomization; OR, odds ratio; SD, standard deviation; SE, standard error (statistical quantity) or sepsis (phenotype code); *SLC5A2*, solute carrier family 5 member 2; SNP, single-nucleotide polymorphism.

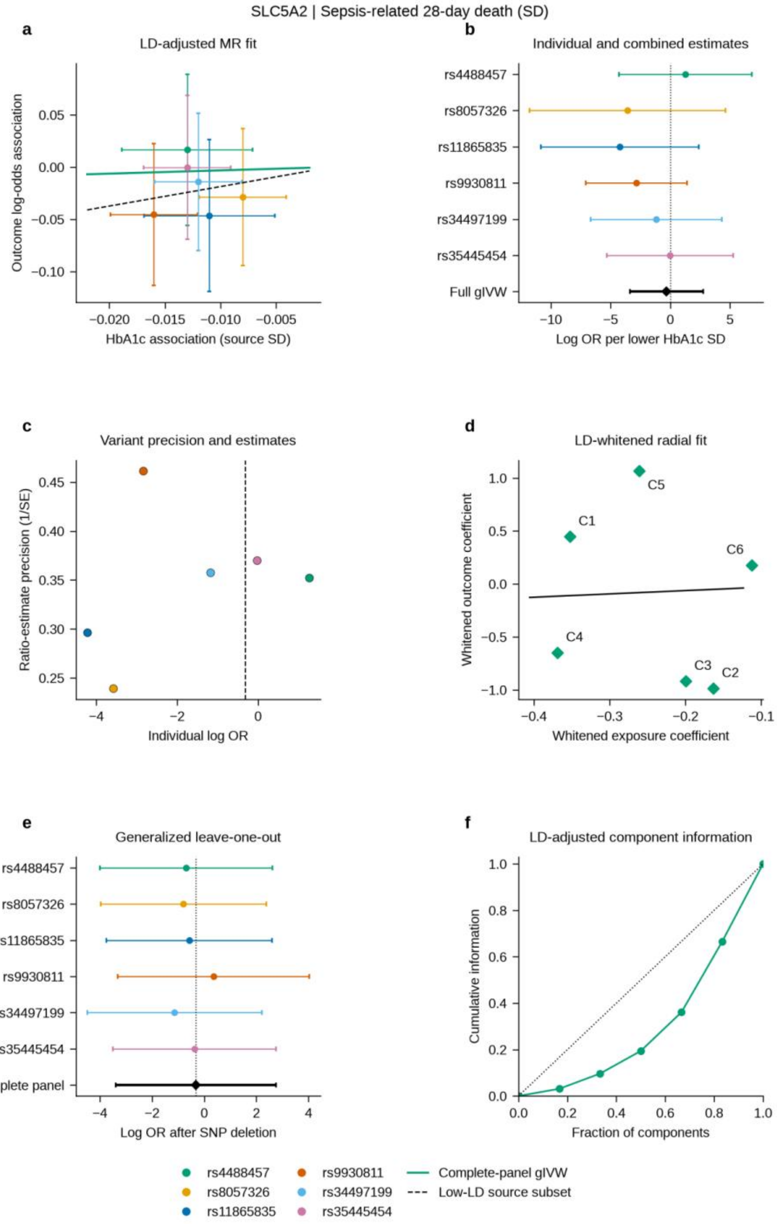

Figure S39. LD-adjusted analyses of *SLC5A2* genetic instruments for sepsis-related 28-day death. Analyses use six available *SLC5A2* SNPs and outcome accession ieu-b-5086. **(a)** SNP–HbA1c and SNP–outcome associations aligned to the exposure effect allele, with horizontal and vertical 95% CIs. The green solid line shows the complete-panel generalized IVW fit, and the black dashed line shows the corresponding fit for the low-LD source subset rs9930811 and rs35445454. **(b)** Individual Wald-ratio estimates (colored circles) and the complete-panel generalized IVW estimate (black diamond), with 95% CIs; the dotted line marks zero. **(c)** Individual ratio estimates plotted against first-order precision (1/SE); the dashed line marks the complete-panel estimate. **(d)** Cholesky-whitened associations and fitted generalized IVW line; green diamonds labeled C1–C6 denote linear combinations of SNP associations obtained in fixed source order. **(e)** Generalized IVW estimates after omitting each labeled SNP and its covariance row and column, with 95% CIs; the black diamond and dotted line mark the complete-panel estimate. **(f)** Cumulative information from normalized squared whitened exposure coefficients, accumulated from smallest to largest; the effective component count is 4.05, and the diagonal indicates equal information across components. Estimates in (b,c,e) are log ORs per one source SD lower HbA1c. Multi-variant fits use the appropriate signed, effect-allele-aligned LD submatrix from 503 European participants in the 1000 Genomes Project Phase 3. Generalized IVW CIs use multiplicative random-effects SEs with dispersion $max[1, \frac{Q}{n-1}]$, where Q is the LD-adjusted heterogeneity statistic and n is the number of variants in the fitted set. Individual-ratio SEs equal $\frac{SE_Y}{|\beta_X|}$, where SE_Y is the outcome association SE and β_X is the HbA1c association coefficient. Colors in (a–c,e) identify the displayed or omitted SNP. Sepsis-related 28-day death was compared with population controls.

Abbreviations: CI, confidence interval; gIVW, generalized inverse-variance weighted; HbA1c, glycated hemoglobin; IVW, inverse-variance weighted; LD, linkage disequilibrium; MR, Mendelian randomization; OR, odds ratio; SD, standard deviation (exposure unit) or sepsis-related 28-day death (phenotype code); SE, standard error; *SLC5A2*, solute carrier family 5 member 2; SNP, single-nucleotide polymorphism.

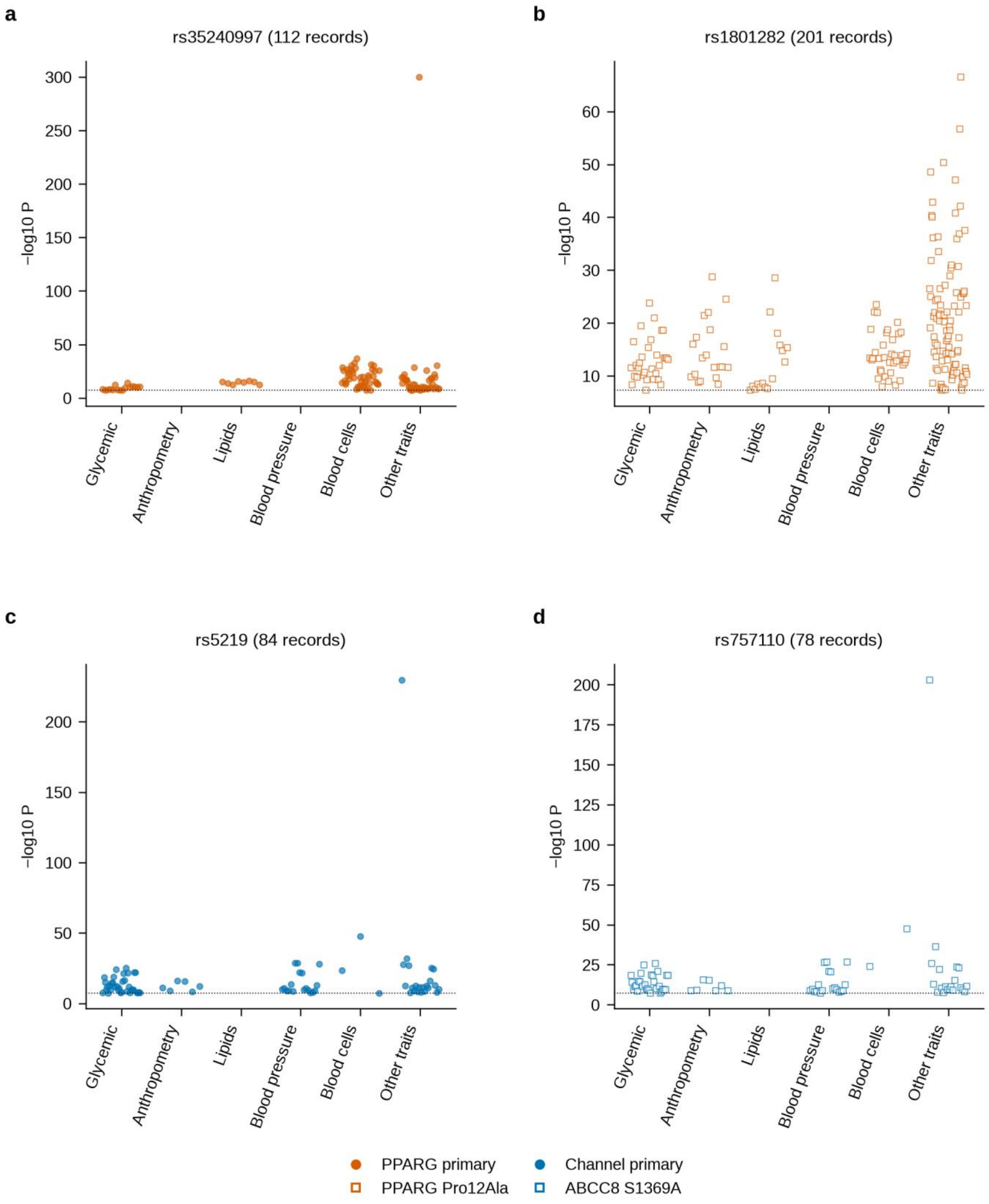

Figure S40. Phenome-wide associations of primary and functional *PPARG* and potassium-channel proxies. **(a)** *PPARG* primary proxy rs35240997 (112 records). **(b)** *PPARG* functional proxy rs1801282, encoding Pro12Ala (201 records). **(c)** Potassium-channel primary proxy rs5219 (84 records). **(d)** *ABCC8* functional proxy rs757110, encoding S1369A (78 records). All 475 OpenGWAS association records retrieved at $p \leq 5 \times 10^{-8}$ are shown as $-\log_{10}(p)$. Records are grouped into glycemic traits, anthropometry, lipids, blood pressure, blood-cell traits, and other traits, and ordered within each domain by full trait title, GWAS accession, and variant identifier. Associations from separate studies of the same trait remain separate records. Filled circles denote primary proxies, and open squares denote functional proxies; vermilion identifies *PPARG* and blue identifies potassium-channel proxies. The dotted horizontal line marks the retrieval threshold. Values returned as $p = 0$ are displayed using a floor of 10^{-300}. The panels retain separate vertical scales.

Abbreviations: *ABCC8*, ATP-binding cassette subfamily C member 8; GWAS, genome-wide association study; *PPARG*, peroxisome proliferator-activated receptor gamma; Pro12Ala, proline-to-alanine substitution at residue 12; S1369A, serine-to-alanine substitution at residue 1369.

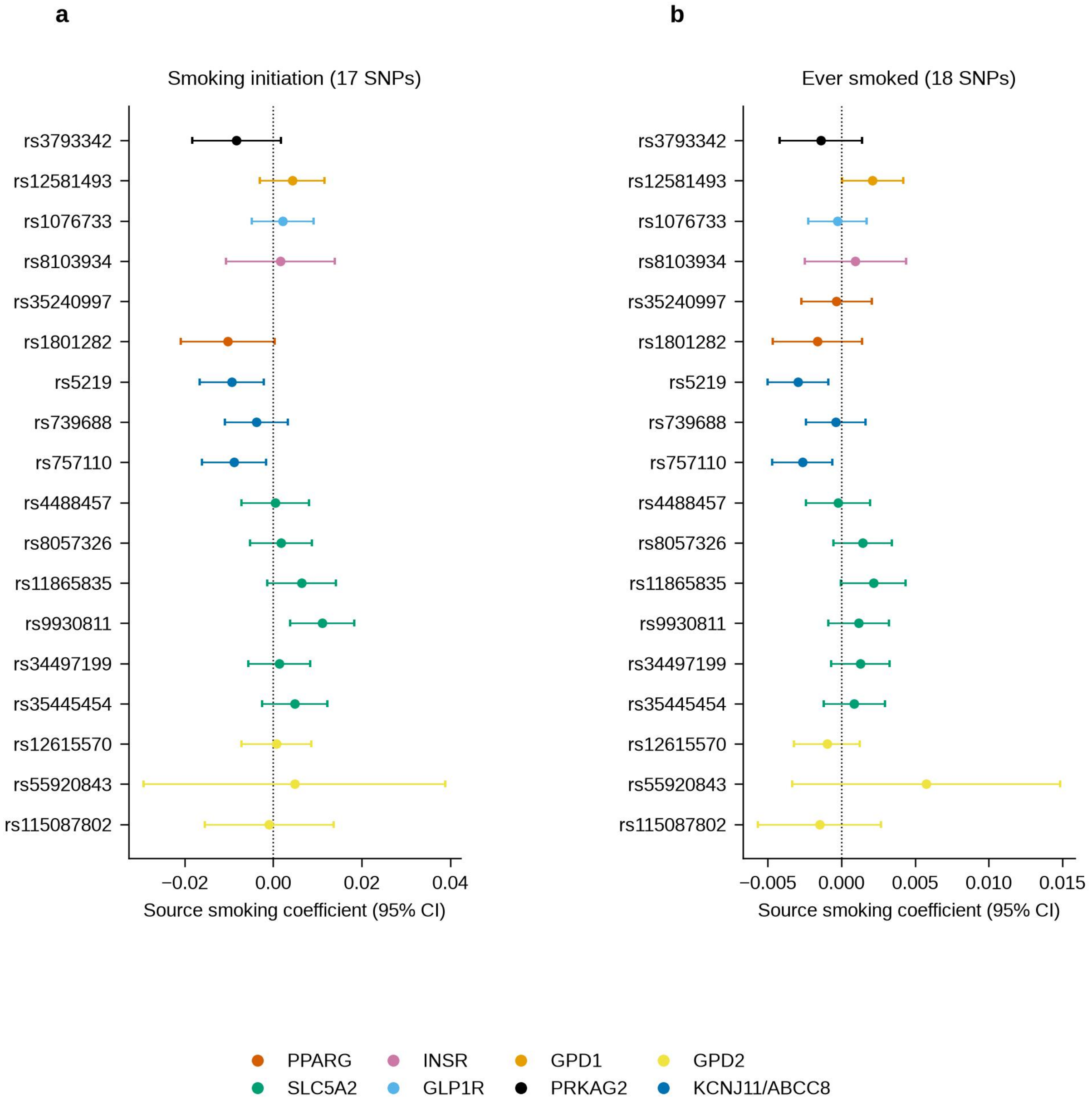

Figure S41. Associations of retained genetic instruments with smoking phenotypes. **(a)** Smoking initiation (GWAS accession ieu-b-4877), with 17 available SNP associations. **(b)** Ever smoking (ukb-b-20261), with 18 SNP associations. Circles show the source GWAS coefficients aligned to the glycemic exposure effect allele, and horizontal bars show 95% CIs. Each panel preserves its source trait scale. Colors identify target genes, as shown in the key, and dotted vertical lines mark zero. The smoking-initiation association for *PPARG* rs35240997 remained unresolved in the source response, leaving its row blank in **(a)**. The figure therefore contains 35 of the 36 planned variant–trait associations.

Abbreviations: *ABCC8*, ATP-binding cassette subfamily C member 8; CI, confidence interval; *GLP1R*, glucagon-like peptide 1 receptor; *GPD1*, glycerol-3-phosphate dehydrogenase 1; *GPD2*, glycerol-3-phosphate dehydrogenase 2; GWAS, genome-wide association study; *INSR*, insulin receptor; *KCNJ11*, potassium inwardly rectifying channel subfamily J member 11; *PPARG*, peroxisome proliferator-activated receptor gamma; *PRKAG2*, protein kinase AMP-activated non-catalytic subunit gamma 2; *SLC5A2*, solute carrier family 5 member 2; SNP, single-nucleotide polymorphism.

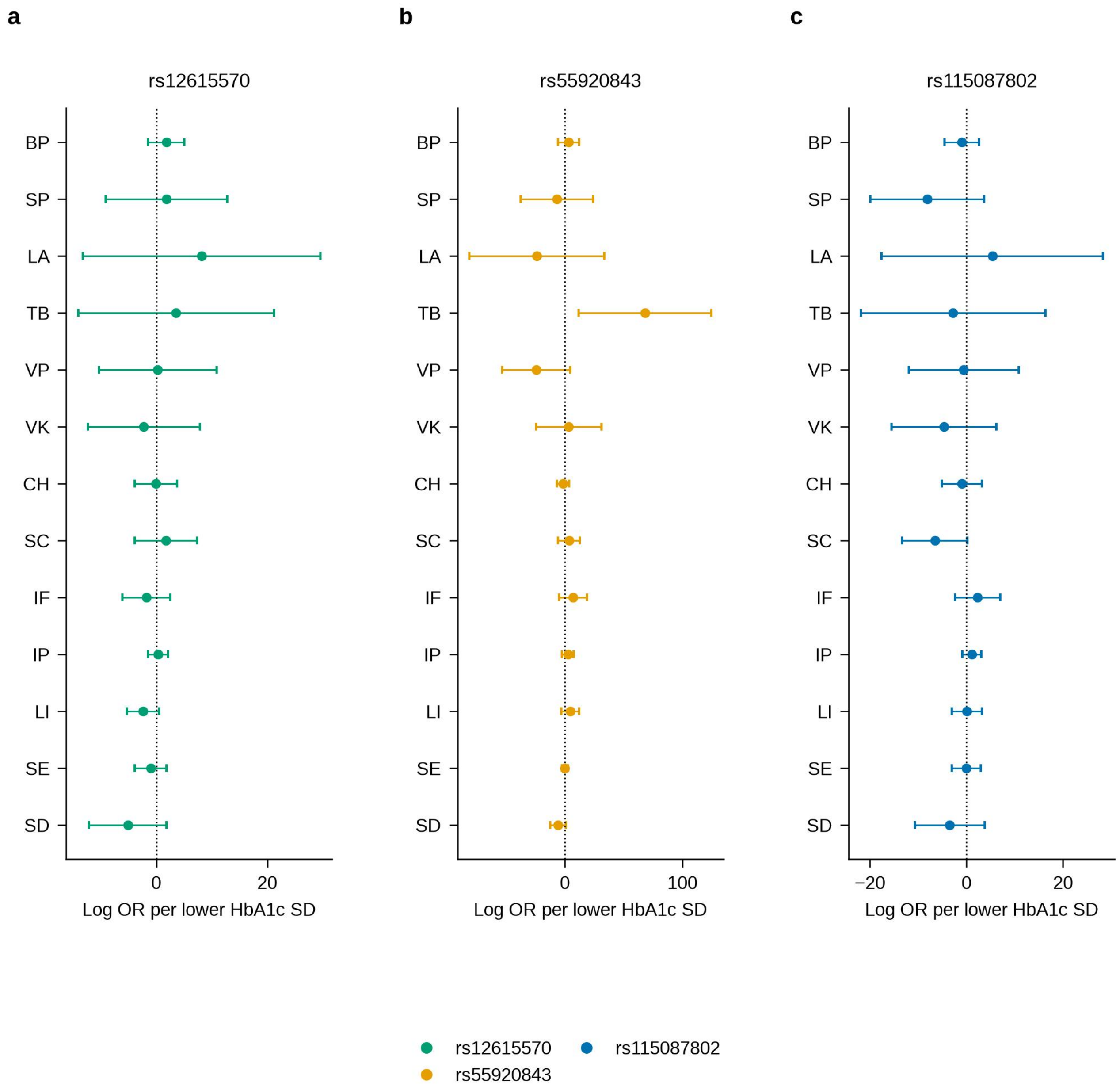

Figure S42. Individual *GPD2* variant associations with infection and sepsis phenotypes. **(a)** rs12615570 (green). **(b)** rs55920843 (orange). **(c)** rs115087802 (blue). Each variant was analyzed separately against the same 13 infection and sepsis outcome accessions using the Wald-ratio method. Circles show log ORs per one source SD lower HbA1c after alignment to the exposure effect allele; horizontal bars indicate 95% CIs. Panels share the same outcome order and use separate horizontal scales. Dotted vertical lines mark the null value of zero.

Abbreviations: BP, bacterial pneumonia; CH, COVID-19 hospitalization; CI, confidence interval; COVID-19, coronavirus disease 2019; *GPD2*, glycerol-3-phosphate dehydrogenase 2; HbA1c, glycated hemoglobin; IF, influenza of all types; IP, influenza and pneumonia composite; LA, lung abscess; LI, other acute lower respiratory infections; OR, odds ratio; SC, severe COVID-19; SD, standard deviation (exposure unit) or sepsis-related 28-day death (phenotype code); SE, sepsis (phenotype code); SP, pneumococcal pneumonia; TB, confirmed respiratory tuberculosis; VK, pneumonia due to a known virus excluding influenza; VP, viral pneumonia.

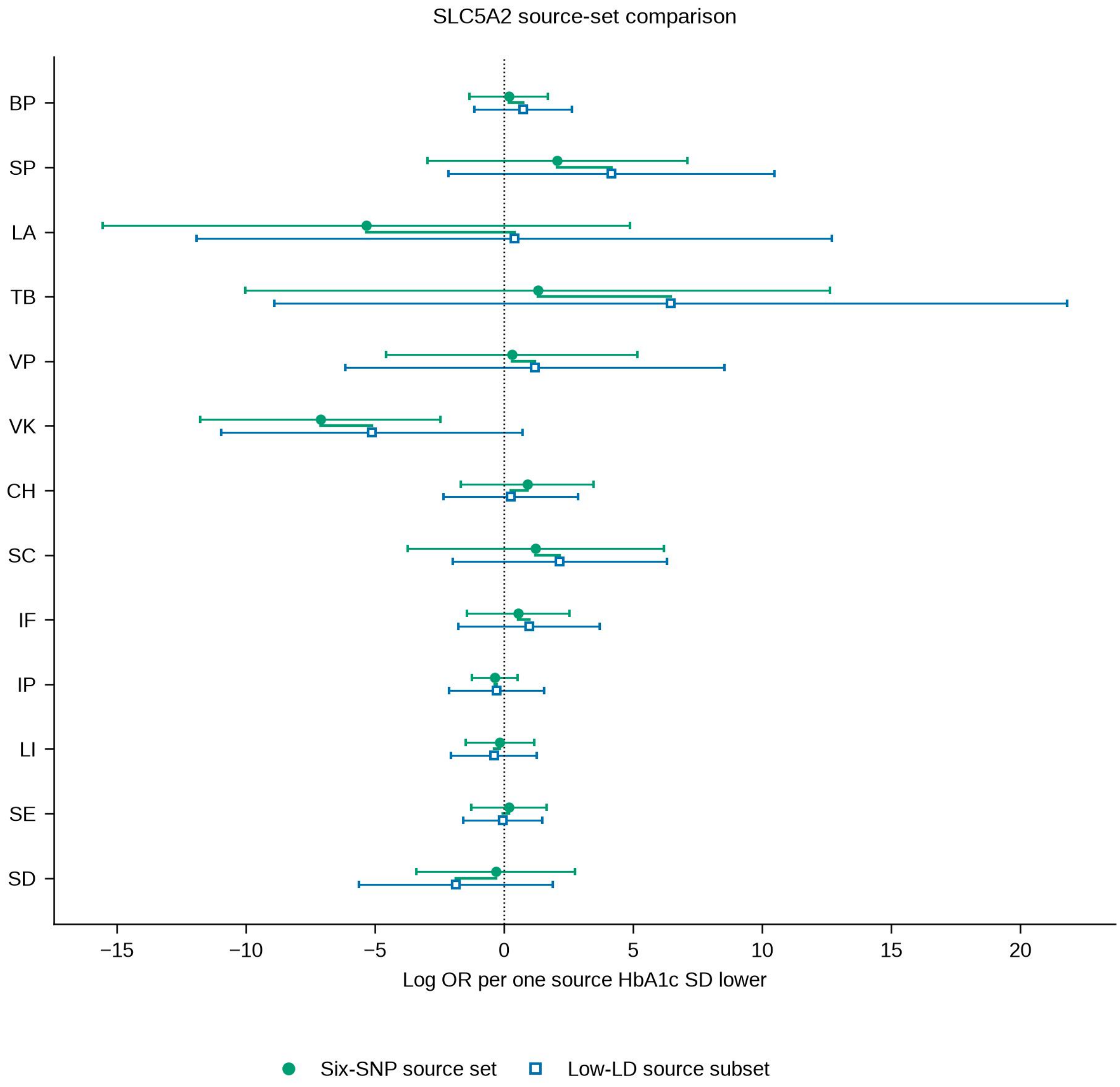

Figure S43. Comparison of *SLC5A2* estimates from the six-SNP source set and the low-LD source subset. Filled green circles show estimates from the complete available *SLC5A2* source set, and open blue squares show estimates from the source subset rs9930811 and rs35445454. The original sets were selected using source-study LD thresholds of $r^2 < 0.8$ and $r^2 < 0.1$, respectively. Estimates are log ORs per one source SD lower HbA1c for 13 matched infection and sepsis outcomes; horizontal bars indicate 95% CIs, and connectors join estimates for the same outcome. Each multi-variant estimate uses generalized IVW with the corresponding signed, exposure-effect-allele-aligned LD submatrix from 503 European participants in the 1000 Genomes Project Phase 3 and multiplicative random-effects CIs. COVID-19 hospitalization includes five variants in the larger set and only rs9930811 in the low-LD subset; the latter estimate is a single-variant Wald ratio. The dotted vertical line marks the null value of zero.

Abbreviations: BP, bacterial pneumonia; CH, COVID-19 hospitalization; CI, confidence interval; COVID-19, coronavirus disease 2019; HbA1c, glycated hemoglobin; IF, influenza of all types; IP, influenza and pneumonia composite; IVW, inverse-variance weighted; LA, lung abscess; LD, linkage disequilibrium; LI, other acute lower respiratory infections; OR, odds ratio; SC, severe COVID-19; SD, standard deviation (exposure unit) or sepsis-related 28-day death (phenotype code); SE, sepsis (phenotype code); *SLC5A2*, solute carrier family 5 member 2; SNP, single-nucleotide polymorphism; SP, pneumococcal pneumonia; TB, confirmed respiratory tuberculosis; VK, pneumonia due to a known virus excluding influenza; VP, viral pneumonia.

Table S1. Associations of primary and functional *PPARG* and *KCNJ11/ABCC8* proxies with 13 matched infection outcomes.

Target / proxy	Outcome	SNPs	OR (95% CI)	P	q (52)	q (156)
KCNJ11/ABCC8 Functional	BP	1	1.27 (0.95–1.69)	0.1042	0.6771	0.7766
KCNJ11/ABCC8 Primary	BP	2	0.83 (0.64–1.08)	0.157	0.6903	0.7766
PPARG Functional	BP	1	1.41 (0.82–2.44)	0.2142	0.7292	0.8712
PPARG Primary	BP	1	1.44 (0.87–2.39)	0.1593	0.6903	0.7766
KCNJ11/ABCC8 Functional	SP	1	0.95 (0.37–2.47)	0.9175	0.9451	0.9988
KCNJ11/ABCC8 Primary	SP	2	1.27 (0.31–5.19)	0.7437	0.8984	0.9745
PPARG Functional	SP	1	0.51 (0.08–3.18)	0.4724	0.8189	0.8813
PPARG Primary	SP	1	0.40 (0.07–2.16)	0.2854	0.8189	0.8813
KCNJ11/ABCC8 Functional	LA	1	1.48 (0.23–9.50)	0.6777	0.8984	0.9745
KCNJ11/ABCC8 Primary	LA	2	0.60 (0.11–3.10)	0.5395	0.8768	0.9435
PPARG Functional	LA	1	0.29 (0.01–9.91)	0.4885	0.8194	0.8813
PPARG Primary	LA	1	3.46 (0.13–93.27)	0.4603	0.8189	0.8813
KCNJ11/ABCC8 Functional	TB	1	2.66 (0.57–12.44)	0.2125	0.7292	0.8712
KCNJ11/ABCC8 Primary	TB	2	0.84 (0.10–7.26)	0.8764	0.9301	0.9979
PPARG Functional	TB	1	111.61 (5.79–2152.96)	0.001793	0.07244	0.1449
PPARG Primary	TB	1	49.95 (3.25–768.33)	0.005039	0.07276	0.1455
KCNJ11/ABCC8 Functional	VP	1	0.67 (0.26–1.68)	0.388	0.8189	0.8813
KCNJ11/ABCC8 Primary	VP	2	0.98 (0.11–8.81)	0.9861	0.9861	0.9988
PPARG Functional	VP	1	1.20 (0.21–6.99)	0.8391	0.9284	0.9745
PPARG Primary	VP	1	0.72 (0.14–3.67)	0.6887	0.8984	0.9745
KCNJ11/ABCC8 Functional	VK	1	1.50 (0.62–3.62)	0.3664	0.8189	0.8813
KCNJ11/ABCC8 Primary	VK	2	0.59 (0.27–1.29)	0.1867	0.7292	0.8568
PPARG Functional	VK	1	3.56 (0.66–19.12)	0.1386	0.6903	0.7766
PPARG Primary	VK	1	1.88 (0.39–8.92)	0.4295	0.8189	0.8813
KCNJ11/ABCC8 Functional	CH	1	0.94 (0.66–1.34)	0.7278	0.8984	0.9745
KCNJ11/ABCC8 Primary	CH	2	0.98 (0.63–1.53)	0.9269	0.9451	0.9988
PPARG Functional	CH	1	1.95 (0.98–3.88)	0.05743	0.4267	0.6478
PPARG Primary	CH	1	1.17 (0.65–2.11)	0.6078	0.8984	0.9745
KCNJ11/ABCC8 Functional	SC	1	0.63 (0.35–1.14)	0.1251	0.6903	0.7766
KCNJ11/ABCC8 Primary	SC	2	1.22 (0.79–1.88)	0.364	0.8189	0.8813
PPARG Functional	SC	1	1.47 (0.52–4.12)	0.4663	0.8189	0.8813
PPARG Primary	SC	1	0.69 (0.29–1.64)	0.3981	0.8189	0.8813

Target / proxy	Outcome	SNPs	OR (95% CI)	P	q (52)	q (156)
KCNJ11/ABCC8 Functional	IF	1	1.05 (0.72–1.54)	0.787	0.9094	0.9745
KCNJ11/ABCC8 Primary	IF	2	0.81 (0.58–1.14)	0.2244	0.7292	0.8712
PPARG Functional	IF	1	1.17 (0.57–2.41)	0.6742	0.8984	0.9745
PPARG Primary	IF	1	1.11 (0.57–2.17)	0.7602	0.8984	0.9745
KCNJ11/ABCC8 Functional	IP	1	1.01 (0.87–1.19)	0.8619	0.9301	0.9887
KCNJ11/ABCC8 Primary	IP	2	0.95 (0.82–1.09)	0.4339	0.8189	0.8813
PPARG Functional	IP	1	1.53 (1.13–2.06)	0.005597	0.07276	0.1455
PPARG Primary	IP	1	1.53 (1.16–2.02)	0.002786	0.07244	0.1449
KCNJ11/ABCC8 Functional	LI	1	1.06 (0.83–1.36)	0.6485	0.8984	0.9745
KCNJ11/ABCC8 Primary	LI	2	0.90 (0.68–1.19)	0.4549	0.8189	0.8813
PPARG Functional	LI	1	1.87 (1.16–3.01)	0.0105	0.09458	0.2128
PPARG Primary	LI	1	1.78 (1.14–2.77)	0.01091	0.09458	0.2128
KCNJ11/ABCC8 Functional	SE	1	0.97 (0.76–1.24)	0.8263	0.9284	0.9745
KCNJ11/ABCC8 Primary	SE	2	0.96 (0.78–1.19)	0.7231	0.8984	0.9745
PPARG Functional	SE	1	0.91 (0.54–1.52)	0.7227	0.8984	0.9745
PPARG Primary	SE	1	0.83 (0.54–1.28)	0.3942	0.8189	0.8813
KCNJ11/ABCC8 Functional	SD	1	1.13 (0.62–2.04)	0.6869	0.8984	0.9745
KCNJ11/ABCC8 Primary	SD	2	0.79 (0.47–1.34)	0.3872	0.8189	0.8813
PPARG Functional	SD	1	1.69 (0.48–5.96)	0.4127	0.8189	0.8813
PPARG Primary	SD	1	1.18 (0.41–3.38)	0.7518	0.8984	0.9745

The table reports odds ratios, 95% confidence intervals, and two-sided unadjusted *p* values for four proxy groups, each evaluated using the same 13 outcome GWAS accessions. *PPARG* is represented by the primary variant rs35240997 and the functional variant rs1801282 (Pro12Ala); the channel pathway is represented by the primary rs5219–rs739688 panel and the functional *ABCC8* variant rs757110 (S1369A). All estimates are calibrated to a genetically proxied 0.1 mmol/L reduction in random glucose. The two-variant channel panel was analyzed using generalized IVW with signed, allele-aligned LD from 503 European participants in 1000 Genomes Phase 3 and multiplicative random-effects standard errors; single-variant estimates were obtained using Wald ratios. The SNPs column gives the number of instruments contributing to each estimate. *q* (52) denotes BH adjustment across the four-proxy, 13-outcome comparison family, and *q* (156) denotes separate BH adjustment across the complete 12-group, 13-outcome target screen. Outcome accessions and case–control counts are provided in Table S2.

Abbreviations: *ABCC8*, ATP-binding cassette subfamily C member 8; BH, Benjamini–Hochberg; BP, bacterial pneumonia; CH, COVID-19 hospitalization; CI, confidence interval; COVID-19, coronavirus disease 2019; GWAS, genome-wide association study; IF, influenza of all types; IP, influenza and pneumonia composite; IVW, inverse-variance weighted; *KCNJ11*, potassium inwardly rectifying channel subfamily J member 11; LA, lung abscess; LD, linkage disequilibrium; LI, other acute lower respiratory infections; OR, odds ratio; *PPARG*, peroxisome proliferator-activated receptor gamma; Pro12Ala, proline-to-alanine substitution at residue 12; *q*, BH-adjusted *p* value; S1369A, serine-to-alanine substitution at residue 1369; SC, severe COVID-19; SD, sepsis-related 28-day death (phenotype code); SE, sepsis (phenotype code); SNP, single-nucleotide polymorphism; SP, pneumococcal pneumonia; TB, confirmed respiratory tuberculosis; VK, pneumonia due to a known virus excluding influenza; VP, viral pneumonia.

Table S2. GWAS resource inventory and metadata for the matched infection outcomes.

(A) Source dataset inventory and case–control metadata.

Population	GWAS ID	PMID	Outcome / trait	Year	Sample size	Number of SNPs	Verified cases	Verified controls	Verified total
European	ebi-a-GCST010783	32404885	COVID-19 (very severe respiratory confirmed vs population)	2020	626151	11678750	NR	NR	NR
European	ebi-a-GCST011075	32404885	COVID-19 (very severe respiratory confirmed vs population)	2020	1388342	9739225	5101	1383241	1388342
European	ebi-a-GCST011076	32404885	COVID-19 (very severe respiratory confirmed vs population)	2020	707407	7475770	NR	NR	NR
European	ebi-a-GCST011077	32404885	COVID-19 (very severe respiratory confirmed vs population)	2020	1059456	7496658	NR	NR	NR
European	ebi-a-GCST011078	32404885	COVID-19 (very severe respiratory confirmed vs population)	2020	1059456	9817241	NR	NR	NR
European	ebi-a-GCST004629	27863252	Neutrophil count	2016	170702	29164935	NR	NR	NR
European	ebi-a-GCST90002351	32888493	Neutrophil count	2020	519288	46379899	NR	NR	NR
European	ebi-a-GCST90002398	32888494	Neutrophil count	2020	408112	40311480	NR	NR	NR
European	ebi-a-GCST90013984	34017140	Neutrophil count	2021	395949	10783686	NR	NR	NR
European	ebi-a-GCST90018968	34594039	Neutrophil count	2021	349856	19054226	NR	NR	NR
European	ebi-a-GCST90025977	34226706	Neutrophil count	2021	443782	4234417	NR	NR	NR
European	ukb-d-30140_irnt	NR	Neutrophil count	2018	349856	13586292	NR	NR	NR
European	ieu-b-34	NR	Neutrophil count	2020	563946	NR	NR	NR	NR
European	finn-b-J10_PNEUMOB ACT	NR	Bacterial pneumoniae	2021	196855	16380385	7987	188868	196855
European	finn-b-J10_PNEUMOB ACTEROTH	NR	Bacterial pneumonia, not elsewhere classified	2021	196382	16380384	NR	NR	NR
European	finn-b-PNEUMOBACT KNOWN	NR	Bacterial pneumonia (organism specified)	2021	215268	16380460	NR	NR	NR
European	finn-b-J10_PNEUMOP NEUMO	NR	Pneumonia due to Streptococcus pneumoniae	2021	189516	16380365	648	188868	189516
European	finn-b-J10_VIRALPNE UMO	NR	Viral pneumonia	2021	189568	16380366	700	188868	189568
European	finn-b-VIRALPNEUM OKNOWN	NR	Viral pneumonia (known virus, not influenza)	2021	213470	16380456	763	212707	213470
European	finn-b-J10_INFLUENZ A	NR	All influenza	2021	193130	16380378	4262	188868	193130

Population	GWAS ID	PMID	Outcome / trait	Year	Sample size	Number of SNPs	Verified cases	Verified controls	Verified total
European	finn-b-J10_INFLUPNEU	NR	Influenza and pneumonia	2021	218792	16380466	29924	188868	218792
European	ieu-b-4980	NR	Sepsis	2021	486484	12243539	11643	474841	486484
European	ieu-b-69	NR	sepsis	2020	462918	12321875	NR	NR	NR
European	ieu-b-4981	NR	Sepsis (28 day death in critical care)	2021	431365	12243324	NR	NR	NR
European	ieu-b-5086	NR	Sepsis (28 day death)	2021	486484	12243487	1896	484588	486484
European	prot-a-1307	29875488	Hepcidin	2018	3301	10534735	NR	NR	NR
European	prot-a-1709	29875488	Neutrophil gelatinase-associated lipocalin	2018	3301	10534735	NR	NR	NR
European	ieu-b-31	NR	Monocyte count	2020	563946	NR	NR	NR	NR
European	ebi-a-GCST90002340	32888493	Monocyte count	2020	521594	46391183	NR	NR	NR
European	ebi-a-GCST90025950	34226706	Monocyte count	2021	443529	4234369	NR	NR	NR
European	ebi-a-GCST90013983	34017140	Monocyte count	2021	395949	10783686	NR	NR	NR
European	ebi-a-GCST90018967	34594039	Monocyte count	2021	349856	19052644	NR	NR	NR
European	ukb-d-30130_irnt	NR	Monocyte count	2018	349856	13586292	NR	NR	NR
European	ebi-a-GCST004625	27863252	Monocyte count	2016	170721	29166012	NR	NR	NR

Table S2 (continued). (B) Additional infection accessions.

GWAS accession	Source phenotype label	Definition relevant to interpretation
ebi-a-GCST90018892	Pulmonary tuberculosis	Dataset-specific pulmonary tuberculosis phenotype
finn-b-AB1_RESP_TUBERCU	Respiratory tuberculosis	Broad respiratory tuberculosis definition
finn-b-AB1_RESP_TUBERCU_CONF	Respiratory tuberculosis, bacteriologically and histologically confirmed	Confirmed tuberculosis category
finn-b-AB1_RESP_TUBERCU_NONCONF	Respiratory tuberculosis, not confirmed bacteriologically or histologically	Nonconfirmed tuberculosis category
finn-b-TBC_RESP	Respiratory tuberculosis	Separate accession; retain its own endpoint identity
finn-b-J10_ABSCLUNGPNEUMO	Abscess of lung	Distinct from broader pneumonia categories
finn-b-J10_ACUTELOWERNAS	Unspecified acute lower respiratory infection	Unspecified lower respiratory infection
finn-b-J10_LOWERINF	Other acute lower respiratory infections	Broader category distinct from unspecified J22 coding
ukb-a-540	Diagnoses – main ICD10: J22 Unspecified acute lower respiratory infection	Main diagnosis
ukb-b-8707	Diagnoses – main ICD10: J22 Unspecified acute lower respiratory infection	Separate GWAS accession for main diagnosis
ukb-b-9369	Diagnoses – secondary ICD10: J22 Unspecified acute lower respiratory infection	Secondary diagnosis

Table S2 (continued). (C) Matched infection outcome accessions.

Outcome	GWAS accession	Cases	Controls	Total
Bacterial pneumonia	finn-b-J10_PNEUMOBACT	7,987	188,868	196,855
Pneumococcal pneumonia	finn-b-J10_PNEUMOPNEUMO	648	188,868	189,516
Lung abscess	finn-b-J10_ABSCLUNGPNEUMO	170	218,125	218,295
Confirmed respiratory tuberculosis	finn-b-AB1_RESP_TUBERCU_CONF	247	217,599	217,846
Viral pneumonia	finn-b-J10_VIRALPNEUMO	700	188,868	189,568
Known-virus pneumonia, excluding influenza	finn-b-VIRALPNEUMOKNOWN	763	212,707	213,470
COVID-19 hospitalization	ebi-a-GCST011081	9,986	1,877,672	1,887,658
Severe COVID-19	ebi-a-GCST011075	5,101	1,383,241	1,388,342
Influenza	finn-b-J10_INFLUENZA	4,262	188,868	193,130
Influenza and pneumonia (composite)	finn-b-J10_INFLUPNEU	29,924	188,868	218,792
Other acute lower respiratory infection	finn-b-J10_LOWERINF	10,103	208,689	218,792
Sepsis	ieu-b-4980	11,643	474,841	486,484
Sepsis-related 28-day death	ieu-b-5086	1,896	484,588	486,484

The resource inventory is presented in three sections: (A) source datasets for infection outcomes, circulating blood-cell counts, and protein traits, with recorded ancestry, accessions, publication identifiers, sample sizes, variant counts, and available case–control metadata; (B) additional infection accessions represented in the analysis records, with their original phenotype labels and definitions; and (C) the 13 outcome accessions used in the matched analyses, with case, control, and total sample counts. Each matched phenotype retains one accession across the primary and functional proxy analyses. The sepsis-related 28-day death phenotype uses population controls, and influenza and pneumonia is a composite source phenotype. Sample sizes refer to individual GWAS resources; contributing populations may overlap, so counts should not be summed across accessions. NR indicates metadata unavailable in the source inventory.

Abbreviations: COVID-19, coronavirus disease 2019; GWAS, genome-wide association study; ICD-10, International Classification of Diseases, 10th Revision; ID, identifier; NR, not reported; PMID, PubMed identifier; SNP, single-nucleotide polymorphism.

Table S3. GWAS resources considered for the type 2 diabetes positive control.

GWAS ID	Year	Trait	Consortium / source	Sample size	Number of SNPs	Population	Cases	Controls	Author
ebi-a-GCST006867	2018	Type 2 diabetes	NR	655666	5030727	European	61714	NR*	Xue A
ebi-a-GCST90018926	2021	Type 2 diabetes	NR	490089	24167560	European	38841	451248	Sakaue S
ebi-a-GCST007515	2018	Type 2 diabetes	NR	298957	190486	European	48286	250671	Mahajan A
ieu-a-1090	2016	Type 2 diabetes	UK Biobank	120286	8404296	European	4040	116246	Wood
ieu-a-26	2012	Type 2 diabetes	DIAGRAM	69033	2473442	European	12171	56862	Morris
ebi-a-GCST90006934	2020	Type 2 diabetes	NR	22326	8884933	European	9978	12348	Cai L
ebi-a-GCST90026417	2021	Type 2 diabetes	NR	12230	5379078	European	9486	2744	Mansour Aly D

The table summarizes seven GWAS resources considered during positive-control screening, including their accessions, recorded years, source or consortium, sample and variant counts, population descriptors, case–control counts, and study authors. The current target-level positive-control analysis uses the common accession ebi-a-GCST90018926, comprising 38,841 cases and 451,248 controls of European ancestry; the corresponding estimates are reported in Table S7. The other entries document the resource inventory used during the original screening. NR denotes metadata not reported in the inventory.

Abbreviations: DIAGRAM, Diabetes Genetics Replication and Meta-analysis consortium; GWAS, genome-wide association study; ID, identifier; NR, not reported; SNP, single-nucleotide polymorphism; UK, United Kingdom.

Table S4. Genetic instrument metadata and approximate instrument strength.

SNP	Target	EA	OA	βX	SEX	PX	EAF	Approx. F	Source role
rs3793342	PRKAG2	A	G	-0.017	0.003	1.95e-07	0.143	32.111111	Retained metformin-related; HbA1c proxy
rs12581493	GPD1	G	C	-0.02	0.002	1.67e-16	0.647	100	Retained metformin-related; HbA1c proxy
rs1076733	GLP1R	A	G	-0.007	0.0016	1.12e-05	0.46	19.140625	Primary gene-region proxy
rs8103934	INSR	T	C	-0.0115	0.0027	3.07e-05	0.09	18.141289	Primary gene-region proxy
rs35240997	PPARG	G	A	-0.0073	0.0019	0.000163	0.21	14.761773	Primary gene-region proxy
rs1801282	PPARG	G	C	-0.008	0.0024	0.000932	0.12	11.111111	Functional sensitivity variant
rs5219	KCNJ11 / ABCC8	T	C	-0.0118	0.0016	9.34e-13	0.39	54.390625	Primary gene-region proxy
rs739688	KCNJ11 / ABCC8	T	C	-0.0056	0.0016	0.000684	0.35	12.25	Primary gene-region proxy
rs757110	ABCC8	C	A	0.0115	0.0017	3.37e-12	0.36	45.761246	Functional sensitivity variant
rs4488457	SLC5A2	G	T	-0.013	0.003	2.9e-07	0.712	18.777778	SGLT2-related; exploratory
rs8057326	SLC5A2	C	T	-0.008	0.002	0.00028	0.523	16	SGLT2-related; exploratory
rs11865835	SLC5A2	C	T	-0.011	0.003	1.34e-05	0.284	13.444444	SGLT2-related; exploratory
rs9930811	SLC5A2	G	A	-0.016	0.002	8.69e-12	0.365	64	SGLT2-related; exploratory
rs34497199	SLC5A2	T	C	-0.012	0.002	5.98e-07	0.475	36	SGLT2-related; exploratory
rs35445454	SLC5A2	T	C	-0.013	0.002	1.24e-07	0.344	42.25	SGLT2-related; exploratory
rs12615570	GPD2	G	A	0.0106183	0.0022852	3.37443e-06	NR	21.590443	GPD2(unit increase)
rs55920843	GPD2	G	T	-0.0456005	0.0092089	7.35258e-07	NR	24.520182	GPD2(unit decrease)
rs115087802	GPD2	A	T	-0.0191976	0.0043445	9.92247e-06	NR	19.526044	GPD2(unit decrease)

The table lists 18 retained primary, functional, and metformin-related variants, including their target assignments, published exposure effect and other alleles, glycemic association coefficients (β_X), standard errors (SE_X), association p values (p_X), effect-allele frequencies, and source-selection roles. Random-glucose and HbA1c associations retain their respective source scales and original exposure-allele coding. Approximate instrument strength was calculated as $F=(\frac{\beta_X}{SE_X})^2$ using the displayed coefficients. The functional *PPARG* and *ABCC8* variants were analyzed separately from their primary proxies, and the three *GPD2* variants retained individual estimates. Exposure p values for the three *GPD2* variants follow the corresponding public HbA1c GWAS records. The source-role column preserves the original selection labels; NR denotes a value not reported in the table.

Abbreviations: *ABCC8*, ATP-binding cassette subfamily C member 8; EA, effect allele; EAF, effect-allele frequency; *F*, instrument-strength *F* statistic; *GLP1R*, glucagon-like peptide 1 receptor; *GPD1*, glycerol-3-phosphate dehydrogenase 1; *GPD2*, glycerol-3-phosphate dehydrogenase 2; GWAS, genome-wide association study; HbA1c, glycated hemoglobin; *INSR*, insulin receptor; *KCNJ11*, potassium inwardly rectifying channel subfamily J member 11; NR, not reported; OA, other allele; *PPARG*, peroxisome proliferator-activated receptor gamma; *PRKAG2*, protein kinase AMP-activated non-catalytic subunit gamma 2; p_X, exposure-association p value; SE_X, standard error of β_X; SGLT2, sodium–glucose cotransporter 2; *SLC5A2*, solute carrier family 5 member 2; SNP, single-nucleotide polymorphism; β_X, exposure association coefficient.

Table S5. Screening and retention of genetic instruments for putative metformin-related targets.

Putative target	Protein-coding gene(s) screened	Screening evidence	Retained final instrument(s)	Selection result	Role in manuscript
AMPK complex	PRKAA1, PRKAA2, PRKAB1, PRKAB2, PRKAG1, PRKAG2, PRKAG3	cis-eQTL screening against HbA1c; approximate colocalization assessed where possible	rs3793342 (PRKAG2)	PRKAG2 retained; three PRKAA1 instruments were excluded because F statistics were <10; other AMPK subunits were not retained after screening	Final target-specific metformin analysis
GPD1	GPD1	cis-eQTL screening against HbA1c plus approximate colocalization	rs12581493	Retained for final Wald-ratio analysis	Final target-specific metformin analysis
GPD2	GPD2	cis-eQTL screening against HbA1c plus approximate colocalization	rs12615570, rs55920843, rs115087802	Retained after clumping and positive-control support for type 2 diabetes	Final target-specific metformin analysis
ETFDH	ETFDH	cis-eQTL screening against HbA1c	None retained in final respiratory-infection tables	Screened but not retained after the final selection pipeline	Screened only
PEN2 lysosomal pathway	PSENEN	cis-eQTL screening against HbA1c	None retained in final respiratory-infection tables	Screened but not retained after the final selection pipeline	Screened only
Overall metformin screening summary	11 genes across 5 putative targets	Independent cis-eQTLs were clumped after target screening	5 final SNPs retained from 70 clumped candidates	Final retained panel comprised GPD1, GPD2, and PRKAG2 instruments	Separate GPD1, PRKAG2, and individual GPD2 analyses

The table summarizes archived screening of 11 genes across five putative targets, using cis-eQTL evidence, HbA1c associations, approximate colocalization where recorded, and variant clumping. Five variants were retained from 70 clumped candidates: *GPD1* rs12581493, *PRKAG2* rs3793342, and *GPD2* rs12615570, rs55920843, and rs115087802. Three *PRKAA1* instruments were excluded because their F statistics were below 10. The selection-result column records the original screening decisions. In the current analyses, *GPD1*, *PRKAG2*, and each *GPD2* variant were evaluated separately using their source-specific HbA1c calibration. The current common-accession type 2 diabetes controls are reported in Table S7, where nominal concordance requires a negative coefficient and $p < 0.05$; *GPD2* rs115087802 has a negative coefficient but does not meet this criterion ($p = 0.1923$).

Abbreviations: AMPK, AMP-activated protein kinase; cis-eQTL, cis-expression quantitative trait locus; *ETFDH*, electron transfer flavoprotein dehydrogenase; *F*, instrument-strength *F* statistic; *GPD1*, glycerol-3-phosphate dehydrogenase 1; *GPD2*, glycerol-3-phosphate dehydrogenase 2; HbA1c, glycated hemoglobin; PEN2, presenilin enhancer 2; *PRKAA1*, protein kinase AMP-activated catalytic subunit alpha 1; *PRKAA2*, protein kinase AMP-activated catalytic subunit alpha 2; *PRKAB1*, protein kinase AMP-activated non-catalytic subunit beta 1; *PRKAB2*, protein kinase AMP-activated non-catalytic subunit beta 2; *PRKAG1*, protein kinase AMP-activated non-catalytic subunit gamma 1; *PRKAG2*, protein kinase AMP-activated non-catalytic subunit gamma 2; *PRKAG3*, protein kinase AMP-activated non-catalytic subunit gamma 3; *PSENEN*, presenilin enhancer, gamma-secretase subunit; SNP, single-nucleotide polymorphism.

Table S6. Estimators and sensitivity analyses by instrument panel.

Analysis panel	Estimator	Sensitivity comparisons	Reporting contrast
Single-SNP targets	Wald ratio	Source-allele and unit verification; T2D control; cross-trait screen	Single-variant glycemic decrement
PPARG proxies	Separate Wald ratios	13 accession-matched functional comparisons; two BH families	Per 0.1 mmol/L glucose decrease
Channel primary panel	Generalized IVW	503-EUR signed LD; Q; 26 leave-one-out fits	Two source-aligned variants
SLC5A2	Generalized IVW	503-EUR signed LD; Q; 77 leave-one-out fits; two-set comparison	Six variants except CH: five
GPD1 / PRKAG2 / GPD2	Separate single-variant ratios	Retained-variant control and source-specific calibration	Distinct target/variant estimates
Smoking screen	Variant–trait associations	35 resolved records; separate BH correction	Six nominal; zero q < 0.05

The table summarizes the estimators, sensitivity comparisons, and exposure contrasts used for each analysis panel. Single-variant proxies were evaluated using Wald ratios, and the primary *KCNJ11*/*ABCC8* and *SLC5A2* panels used generalized IVW with signed, allele-aligned LD from 503 European participants in 1000 Genomes Phase 3 and multiplicative random-effects standard errors. LD-aware leave-one-out refitting yielded 26 channel and 77 *SLC5A2* deletion estimates. The larger *SLC5A2* panel included six variants for 12 outcomes and five for COVID-19 hospitalization, with a separate comparison against the published low-LD source subset. *GPD1*, *PRKAG2*, and individual *GPD2* variants retained separate source-calibrated estimates. BH adjustments were applied separately to the 52 matched proxy comparisons, the 156-estimate target screen, and the 35 resolved smoking variant–trait associations. Nominal significance denotes $p < 0.05$, and the BH-adjusted threshold is $q < 0.05$.

Abbreviations: *ABCC8*, ATP-binding cassette subfamily C member 8; BH, Benjamini–Hochberg; CH, COVID-19 hospitalization; COVID-19, coronavirus disease 2019; EUR, European-ancestry reference population; *GPD1*, glycerol-3-phosphate dehydrogenase 1; *GPD2*, glycerol-3-phosphate dehydrogenase 2; IVW, inverse-variance weighted; *KCNJ11*, potassium inwardly rectifying channel subfamily J member 11; LD, linkage disequilibrium; *PPARG*, peroxisome proliferator-activated receptor gamma; *PRKAG2*, protein kinase AMP-activated non-catalytic subunit gamma 2; *Q*, LD-aware heterogeneity statistic; *q*, BH-adjusted *p* value; *SLC5A2*, solute carrier family 5 member 2; SNP, single-nucleotide polymorphism; T2D, type 2 diabetes.

Table S7. Type 2 diabetes positive-control estimates for retained target proxies in a common GWAS.

Target / variant	SNPs	β	SE	P	Exposure decrement	Direction
GLP1R Primary	1	-0.35	0.09	0.0001147	0.1 mmol/L glucose lower	Negative
INSR Primary	1	-0.29	0.11	0.006034	0.1 mmol/L glucose lower	Negative
PPARG Primary	1	-1.00	0.13	8.103e-15	0.1 mmol/L glucose lower	Negative
PPARG Functional	1	-1.33	0.14	1.003e-21	0.1 mmol/L glucose lower	Negative
KCNJ11/ABCC8 Primary	2	0.39	0.31	0.2057	0.1 mmol/L glucose lower	Positive
KCNJ11/ABCC8 Functional	1	-0.53	0.06	2.832e-21	0.1 mmol/L glucose lower	Negative
SLC5A2 Six-SNP source set	6	-0.64	0.29	0.02811	1 source HbA1c SD lower	Negative
GPD1 Retained	1	-1.11	0.34	0.001096	1 source HbA1c SD lower	Negative
PRKAG2 Retained	1	-1.62	0.54	0.0027	1 source HbA1c SD lower	Negative
GPD2 rs12615570	1	-1.44	0.70	0.03868	1 source HbA1c SD lower	Negative
GPD2 rs55920843	1	-2.71	1.08	0.01189	1 source HbA1c SD lower	Negative
GPD2 rs115087802	1	-0.69	0.53	0.1923	1 source HbA1c SD lower	Negative

Results are shown for 12 target–proxy groups using the type 2 diabetes outcome ebi-a-GCST90018926, comprising 38,841 cases and 451,248 controls of European ancestry. β denotes the calibrated log odds ratio for a genetically proxied reduction of 0.1 mmol/L in random glucose or one source SD in HbA1c, as specified for each row. Exposure and outcome coefficients were aligned to the published exposure effect allele. Single-variant estimates were obtained using Wald ratios; the two-variant primary *KCNJ11*/*ABCC8* panel and six-variant *SLC5A2* panel were analyzed using generalized IVW with signed, allele-aligned European reference LD and multiplicative random-effects standard errors. The three *GPD2* variants were analyzed separately. The table reports the instrument count, coefficient, standard error, two-sided unadjusted *p* value, exposure decrement, and coefficient direction. A negative coefficient with $p < 0.05$ defines an expected-direction nominal positive control.

Abbreviations: *ABCC8*, ATP-binding cassette subfamily C member 8; *GLP1R*, glucagon-like peptide 1 receptor; *GPD1*, glycerol-3-phosphate dehydrogenase 1; *GPD2*, glycerol-3-phosphate dehydrogenase 2; GWAS, genome-wide association study; HbA1c, glycated hemoglobin; *INSR*, insulin receptor; IVW, inverse-variance weighted; *KCNJ11*, potassium inwardly rectifying channel subfamily J member 11; LD, linkage disequilibrium; *PPARG*, peroxisome proliferator-activated receptor gamma; *PRKAG2*, protein kinase AMP-activated non-catalytic subunit gamma 2; SD, standard deviation; SE, standard error; *SLC5A2*, solute carrier family 5 member 2; SNP, single-nucleotide polymorphism; β, calibrated log odds ratio.
